# Efficient Synthesis of 1*H*-Benzo[4,5]imidazo[1,2-*c*][1,3]oxazin-1-one Derivatives Using Ag_2_CO_3_/TFA-Catalyzed 6-*endo-dig* Cyclization: Reaction Scope and Mechanistic Study

**DOI:** 10.3390/molecules28052403

**Published:** 2023-03-06

**Authors:** Abdelkarim El Qami, Badr Jismy, Mohamed Akssira, Johan Jacquemin, Abdellatif Tikad, Mohamed Abarbri

**Affiliations:** 1 Laboratoire de Physico-Chimie des Matériaux et des Electrolytes pour l’Energie (PCM2E), EA 6299, Faculté des Sciences et Techniques, Avenue Monge, Faculté des Sciences, Université de Tours, Parc de Grandmont, 37200 Tours, France; 2 Laboratoire de Chimie Physique et Biotechnologies des Biomolécules et des Matériaux (LCP2BM), FSTM, Université Hassan II de Casablanca, B.P. 146, Mohammedia 28800, Morocco; 3 Materials Science and Nano-Engineering (MSN) Department, Mohammed VI Polytechnic University (UM6P), Lot 660–Hay Moulay Rachid, Benguerir 43150, Morocco; 4 Laboratoire de Chimie Moléculaire et Substances Naturelles, Faculté des Sciences, Université Moulay Ismail, B.P. 11201, Zitoune, Meknès 50050, Morocco

**Keywords:** 6-*endo-dig* cyclization, silver, oxacyclization, catalytic process, 1H-benzo[4,5]imidazo[1,2-*c*][1,3]oxazin-1-ones, computational study, DFT

## Abstract

A small library of 1*H*-benzo[4,5]imidazo[1,2-*c*][1,3]oxazin-1-one derivatives was prepared in good to excellent yields, involving a Ag_2_CO_3_/TFA-catalyzed intramolecular oxacyclization of *N-Boc*-2-alkynylbenzimidazole substrates. In all experiments, the 6-*endo*-*dig* cyclization was exclusively achieved since the possible *5-exo-dig* heterocycle was not observed, indicating the high regioselectivity of this process. The scope and limitations of the silver catalyzed *6-endo-dig* cyclization of *N*-*Boc*-2-alkynylbenzimidazoles as substrates, bearing various substituents, were investigated. While ZnCl_2_ has shown limits for alkynes with an aromatic substituent, Ag_2_CO_3_/TFA demonstrated its effectiveness and compatibility regardless of the nature of the starting alkyne (aliphatic, aromatic or heteroaromatic), providing a practical regioselective access to structurally diverse 1*H*-benzo[4,5]imidazo[1,2-*c*][1,3]oxazin-1-ones in good yields. Moreover, the rationalization of oxacyclization selectivity in favor of *6-endo-dig* over *5-exo-dig* was explained by a complementary computational study.

## 1. Introduction

**Figure 1:**
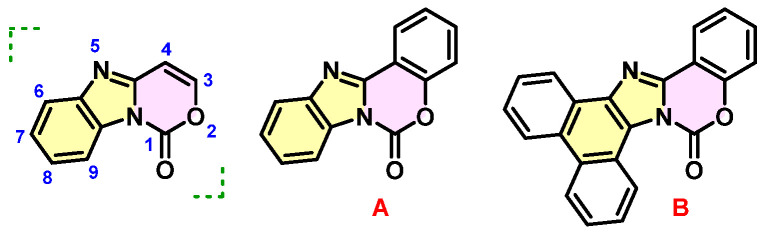
Several fused active heterocycles containing the 1*H*-benzo[4,5]imidazo[1,2-*c*][1,3]oxazin-1-one core.

Fused heteropolycycles containing nitrogen and oxygen in the skeleton constitute an important class of heterocyclic compounds that can be found in numerous biologically active compounds [1,2,3] and natural products [4,5]. Among them, the structural motif 1*H*-benzo[4,5]imidazo[1,2-*c*][1,3]oxazin-1-one is present in pharmacologically interesting molecules, which display fungicidal activity (compound **A**, Figure 1) [6]. In addition, compound **B** (Figure 1) has been known as a potent recognition site for the detection of the highly toxic phosgene gas [7].

Functionalized alkynes at the *ortho* position of the carboalkoxy group are among the most common substrates used by organic chemists to generate new polycyclic heterocycles [8,9,10,11,12,13,14,15,16,17,18,19,20]. Thus, the electrophilic activation of a triple bond under acidic conditions or in the presence of a transition metal triggers heterocyclization by intramolecular nucleophilic attack. In most cases, these processes involve the regioselective 6-*endo*-*dig* cyclization, as opposed to 5-*exo*-*dig*, providing, for example, the total synthesis of several natural products such as Scoparine A and B [21], Thunberginol A [22], (−)-Citreoisocoumarinol, (−)-Citreoisocoumarin, (−)-12-*epi*-Citreoisocoumarinol and (−)-Mucorisocoumarins A and B [23].

Although the activation of 2-alkynylbenzoates has been extensively studied using a series of metal species including Au [23,24,25,26], Ag [17,27,28], Pt [29,30], In [31], B [15,32], Cu [33,34,35] and Fe [20,36], a limited number of heterocyclizations starting from alkynyl O-alkylcarbamates have been reported in the literature, which have been promoted with only three transition metal catalysts: gold [19,37,38,39], silver [40] and zinc [41,42].

**Figure 2:**
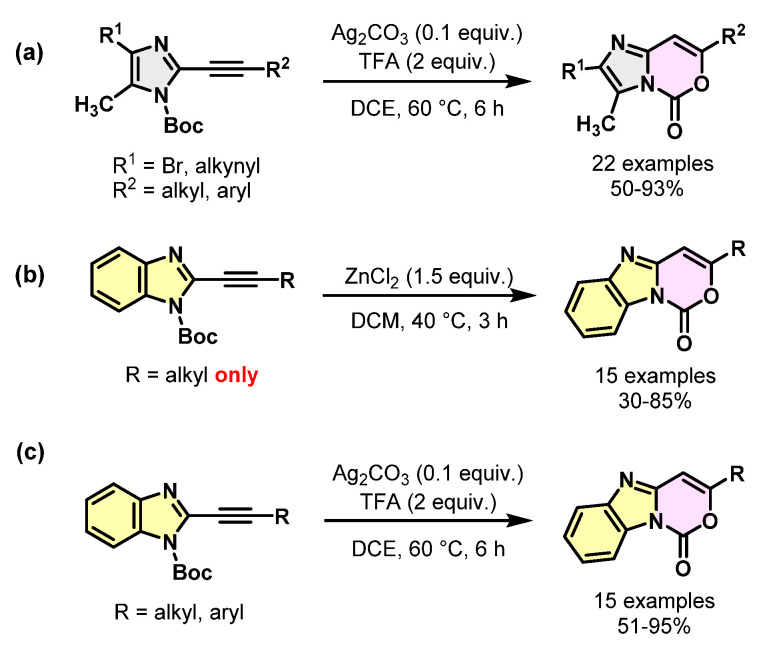
Known procedures for the cyclization of *N*-Boc-2-alkynylimidazoles and *N*-Boc-2-alkynylbenzimidazoles. (**a**) [40]; (**b**) [41]; (**c**) This work.

Recently, we have developed Ag_2_CO_3_/TFA as a new tandem catalyst for intramolecular oxacyclization of *N*-Boc-2-alkynyl-4-bromo(alkynyl)-5-methylimidazole producing 3-methylimidazo[1,2-*c*][1,3]oxazin-5-one derivatives (Figure 2a) [40]. In order to study the efficiency of Ag_2_CO_3_/TFA as a catalytic system to promote heterocyclization from other substrates, herein we report the extension of our approach to *N*-Boc-2-alkynylbenzimidazoles, giving access to 1*H*-benzo[4,5]imidazo[1,2-*c*][1,3]oxazin-1-one derivatives (Figure 2c). It is noteworthy that the synthesis of benzimidazoxazinone derivatives has been already described in the literature using ZnCl_2_-mediated deprotective annulation (Figure 2b) [41]. However, this methodology was limited to aliphatic alkyne since alkynes with an aromatic substituent were not cyclized in this study. 

During the preparation of the desired 1*H*-benzo[4,5]imidazo[1,2-*c*][1,3]oxazin-1-one 5, the byproduct AgTFA may be recycled from the Ag_2_CO_3_/TFA system catalyst for other applications. Considering the environmental impact, the Ag_2_CO_3_/TFA is an environmentally benign system catalyst.

## 2. Results and Discussion

**Scheme 1:**
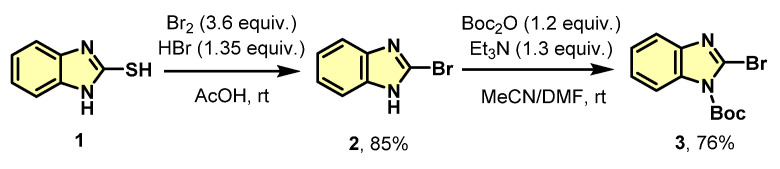
Synthesis of *N*-Boc-2-bromobenzimidazole **3**.

Our synthesis begins with the generation of 2-brominated benzimidazole **2**, starting from 2-mercaptobenzimidazole **1**, following the known reported procedure [18]. Selective bromination of **1** was performed with bromine and hydrogen bromide in acetic acid at room temperature according to a literature procedure [19], providing 85% yield. Compound **2** was subsequently protected by a *tert*-butoxycarbonyl group using (Boc)_2_O as the reagent in the presence of triethylamine in a mixture of MeCN/DMF (1:1) at room temperature leading to compound **3** in 76% yield (Scheme 1). The *N*-Boc-2-bromobenzimidazole **3** served as a building block to introduce substituted alkynes at the C-2 position via the Sonogashira cross-coupling reaction.

In order to prepare a series of *N*-Boc-2-alkynylbenzimidazoles **4** as substrates which could undergo intramolecular cyclization, compound **3** was engaged in Sonogashira cross-coupling with several alkynes.

**Scheme 2:**
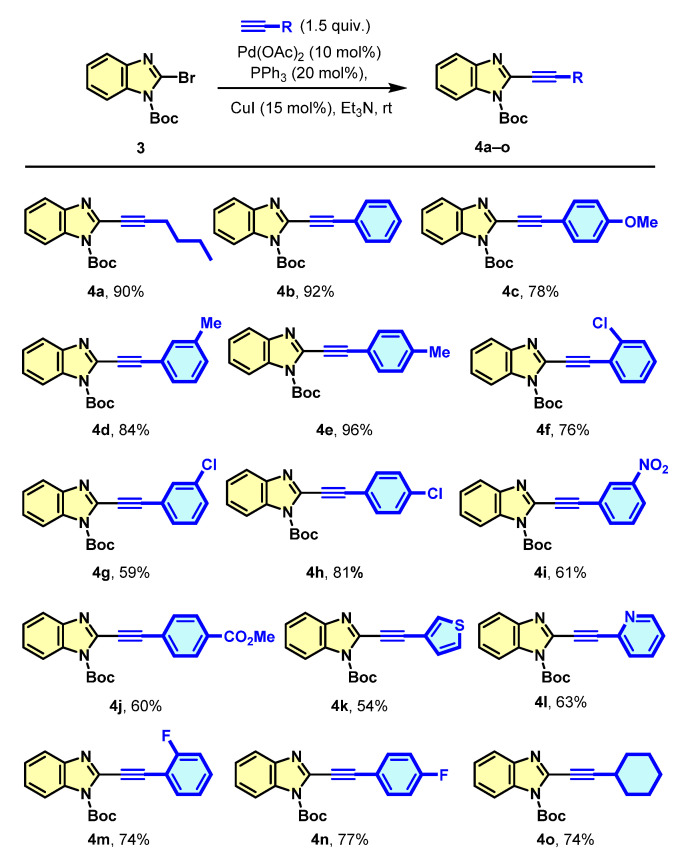
Scope of the Sonogashira coupling of *N*-Boc-2-bromobenzimidazole **3** with various alkynes.

After screening the various conditions, the use of phenylacetylene (1.5 equiv.), Pd(OAc)_2_ (10 mol%), PPh_3_ (20 mol%) and CuI (15 mol%) in triethylamine at room temperature proved to be the most appropriate choice conditions for obtaining alkynylated product **4b** in excellent yield (Scheme 2).

The scope and limitation of the Sonogashira cross-coupling were investigated starting from *N*-Boc-2-bromobenzimidazole **3** with various terminal alkynes (Scheme 2). As illustrated in Scheme 2, it was found that the nature of terminal alkynes (aliphatic, aromatic or heteroaromatic) did not dramatically affect the efficiency of this cross-coupling, since, in all cases, the expected compounds were obtained in moderate to good yields. Thus, this procedure is compatible with several substituents (electron-donating or electron-withdrawing groups) on the aryl rings (methoxy, methyl, chlorine, nitro, ester and fluorine groups). Moreover, under the same conditions, alkynes bearing an heteroaryl groups, such as 2-thienyl or 2-pyridyl, were also able to be introduced in satisfactory yields [**4k** (54%) and **4l** (63%)].

Initially, the treatment of *N*-Boc-2-hexynylbenzimidazole **4a** with ZnCl_2_ (1.5 equiv.) in dichloromethane at 40 °C gave successfully and exclusively the tricyclic core **5a** in 80% isolated yield. However, the reaction starting from *N*-Boc-2-phenylethynylbenzimidazole **4b** required a longer time and gave a mixture of the expected product **5b** along with the starting material **4b** in a 60/40 ratio, respectively (Table 1, entry 2). Increasing the temperature to 60 °C slightly improved the **4b**/**5b** ratio from (60:40) to (50:50) (Table 1, entry 3). These results clearly indicate the inefficiency of ZnCl_2_ to promote intramolecular cyclization from *N*-Boc-2-alkynylbenzimidazole derivatives when the substituent of alkyne is an aromatic ring, such as phenyl.

To our delight, the combination of a catalytic amount of Ag_2_CO_3_ (0.1 equiv.) and TFA (2 equiv.) significantly improved the conversion of the starting material to 85% while the reaction time decreased to 24 h (Table 1, entry 4). Performing the reaction with dichloroethane as a solvent, instead of dichloromethane, resulted in a complete conversion of the starting material **4b** to the target heterocycle **5b**, which was isolated in an excellent yield of 90% after purification by silica-gel column chromatography (Table 1, entry 5). Otherwise, dichloroethane has a greater impact on the conversion rate of the reaction, which could be related to its higher boiling point compared to dichloromethane. A suitable solvent is crucial to this reaction. Under the same conditions, decreasing the temperature to 40 °C significantly affected the formation of the desired product **5b**, since the conversion of substrate **4b** remained incomplete, despite a longer reaction time of 24 h, proving the importance of heating at 60 °C to obtain a full conversion (Table 1, entry 6).

**Scheme 3:**
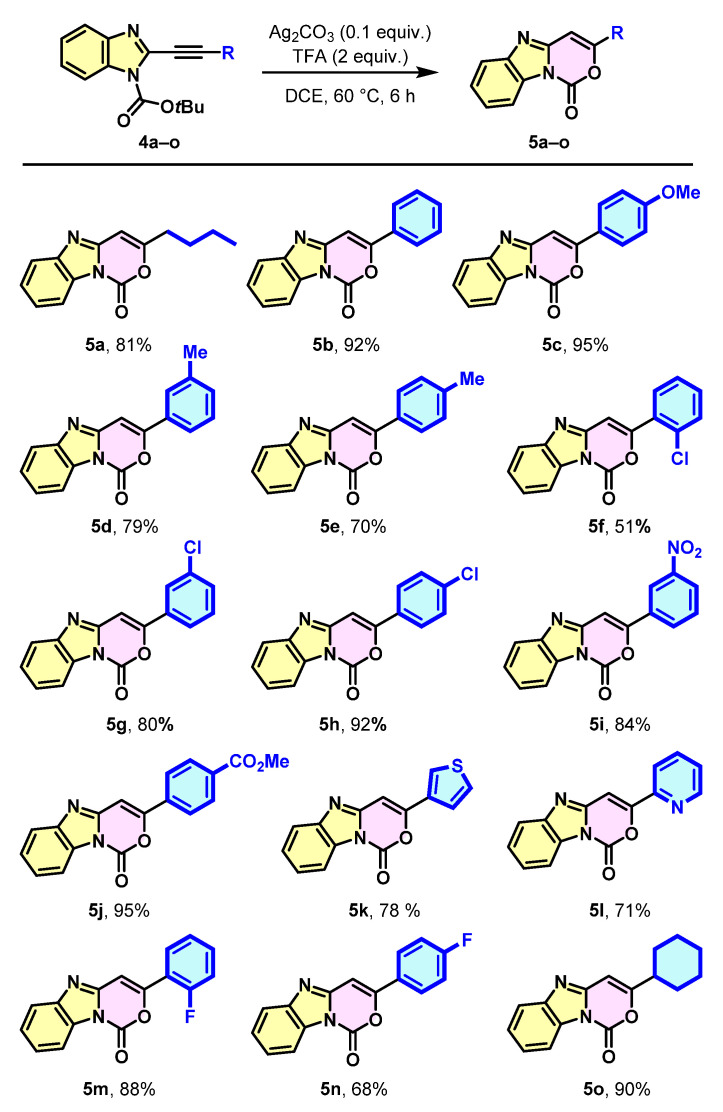
Ag_2_CO_3_/TFA-mediated annulation of *N*-Boc-2-alkynyl-benzimidazoles **4a**–**o**.

The best results in terms of the time and yields were obtained with Ag_2_CO_3_/TFA as a catalytic system. Having established the required conditions for efficient annulation, various *N*-Boc-2-alkynyl(arylethynyl)benzimidazoles **4a**–**o**, which are suitable substrates to undergo intramolecular cyclization, were subjected to these optimized reaction conditions in order to study the scope and limitations of our process (Scheme 3). All reaction mixtures were stirred at 60 °C until the starting material was completely consumed, monitored by thin-layer chromatography (TLC) using a mixture of petroleum ether/ethyl acetate (*v*/*v* = 8/2) as the eluent. The oxacyclization conditions were found to be compatible with a variety of R groups in starting materials **4a**–**o**, such as alkyl, cycloalkyl, aryl and heteroaryl, bearing electron-withdrawing or -donating substituents.

The nature of the substituents on the phenyl ring slightly affected the outcome of the cyclization. As given in Scheme 3, the reactions with the substrates having electron-donating groups, such as methoxy and methyl, were performed efficiently, affording the expected compounds **5c**–**e** in good yields. Benzimidazoles containing chlorine atom as an electron-withdrawing group at the *ortho*, *meta* or *para* position **4f**–**h** were successfully cyclized, giving access to the desired products **5f** (51%), **5g** (80%) and **5h** (92%). As observed, the steric hindrance of the *ortho*-position seems to substantially influence the cyclization efficiency. 

The presence of a strong electron-withdrawing group, such as nitro or carbomethoxy function on the phenylethynyl group, promote the 6-*endo*-*dig* cyclization, leading to new fused benzimidazoles in excellent yields [**5i** (84%) and **5j** (95%)]. Encouraged by the good results obtained with different aryl R groups, benzimidazoles bearing ethynylheteroaryl were exposed under the same silver-catalyzed oxacyclization conditions. Interestingly, substrates having an heteroaromatic ring on the triple bond, such as 3-thienyl and 2-pyridyl, were found to also be compatible and exclusively provided the corresponding heterocycles **5k** and **5l** in 78% and 71% yields, respectively. The intramolecular cyclization of substrates bearing a fluorine atom on the phenyl ring, R = 2-fluorophenyl and R = 4-fluorophenyl, works well, giving the desired compounds in good yields [**5m** (88%) and **5n** (68%)]. When the alkyne substituent is a bulky cyclohexyl group, the cyclization proceeded smoothly and provided the desired heterocycle **5o** in excellent 90% yield. It is noteworthy that among the two possible oxacyclization products **5** and **6**, only the 6-*endo*-*dig* cyclization heterocycle **5** was formed in all experiments, with variations mainly in the isolated yields. 

### Theoretical Calculation (Computational Studies)

**Scheme 4:**
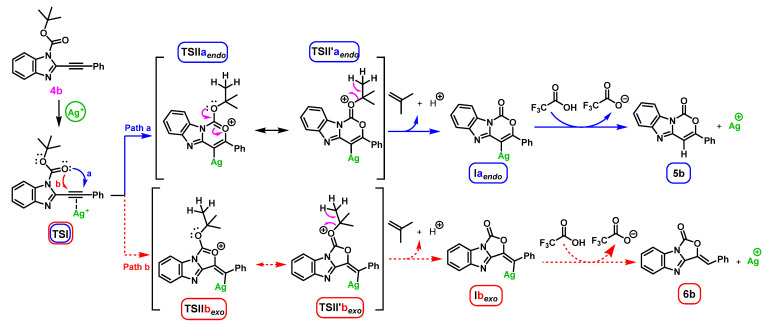
Projected computational reaction mechanism.

To investigate the reaction pathway, and in particular, to understand the mechanism and stereoselectivity of the annulation reaction catalyzed by Ag_2_CO_3_ and TFA, a series of computational experiments were performed by density functional theory (DFT) calculations. The proposed mechanism for the competing intramolecular cyclization pathways of the target benzimidazoxazinone **5b** is outlined in Scheme 4.

**Figure 3:**
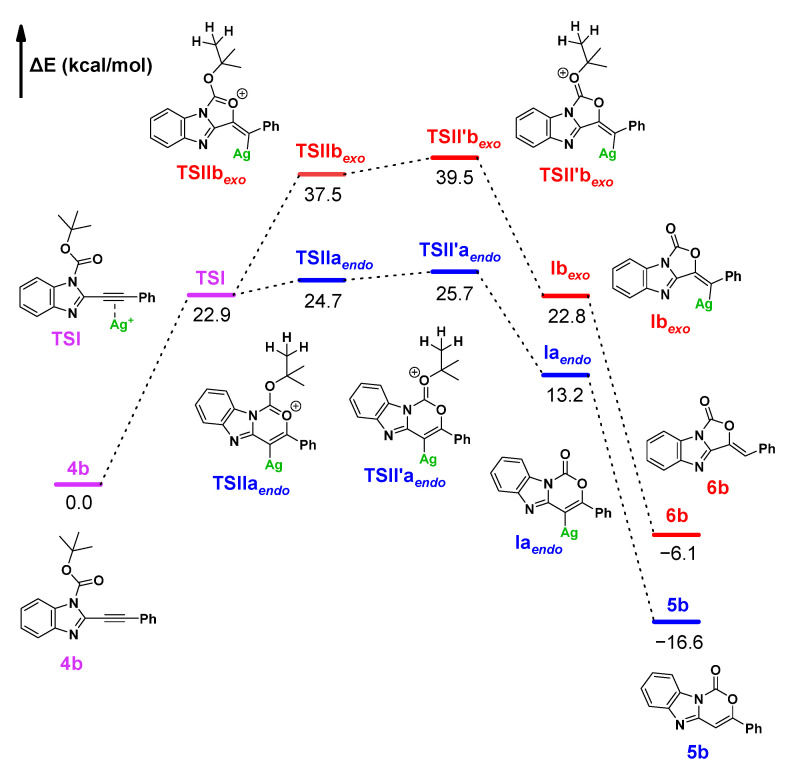
DFT-computed relative free energy of each intermediate and transition state.

In order to justify the expected 6-*endo* annulation, the intermediates and the transition states (TS) of both cyclization pathways [6-*endo-dig* (path a, blue) or 5-*exo-dig* (path b, red)] were computed. All the structures were optimized at the B3LYP level in the gas phase and then in DCE (see the Supplementary Materials for details). The energy profiles of different reaction pathways are depicted in Figure 3.

As shown in Figure 3, the energy barriers of the transition states **TSIIa*_endo_***–**TSII’a*_endo_***, the intermediate **Ia***_endo_* and the product **5b** for 6-*endo-dig* oxacyclization (path a, blue) are much lower than those of 5-*exo-dig* (path b, red). This suggests that the preferential 6-*endo-dig* lactonization of compound **4b** is favored both kinetically and thermodynamically.

To confirm our mechanistic hypothesis, we extended the earlier studies to the calculated natural population analysis (NPA) of each carbon in the alkyne group for all starting materials **4a**–**o** (Table 2).

The calculated NPA revealed that the positive charge is located on the carbon atom denoted β leading to the 6-*endo-dig* cyclization, while the Cα leading to the undesired annulation (5-*exo-dig*) product **6b** is negatively charged, which is in excellent agreement with the experiment, regardless of the nature of the alkyne substituent.

## 3. Materials and Methods

### 3.1. General Information

All reactions were performed under an inert atmosphere of argon in oven-dried glassware equipped with a magnetic stir bar. Solvents for reactions were obtained from Thermo Fisher Scientific in extra dry quality and stored under argon over activated 3 Å sieves. All reagents were purchased from Fluorochem and used as received without additional purification. Reactions were monitored by thin-layer chromatography (TLC) analysis using silica gel 60 F254 plates. All products were visualized by exposure to UV light (longwave at 365 nm or shortwave at 254 nm). Column chromatography was performed using silica gel 60 (230–400.13 mesh, 0.040–0.063 mm). Eluents were distilled by the standard methods before each use. All new compounds were characterized by NMR spectroscopy (1H, 19F and 13C), high-resolution mass spectroscopy (HRMS) and melting point (if solids). NMR spectra were recorded at 300 MHz for ^1^H, 282 MHz for ^19^F, and 75 MHz for ^13^C with a Bruker^®^ 300 MHz NMR spectrometer. Proton and carbon magnetic resonance spectra (^1^H NMR and ^13^C NMR) were recorded using tetramethylsilane (TMS) as an external standard and CDCl_3_ (7.28 ppm for ^1^H NMR and 77.04 ppm for ^13^C NMR) or DMSO-*d6* (2.50 ppm for ^1^H NMR and 40.0 ppm for ^13^C NMR) as internal standards. ^19^F spectra were unreferenced. Data for NMR are reported as follows: chemical shift (δ ppm), multiplicity (s = singlet, d = doublet, t = triplet, q = quartet, sep = septet, m = multiplet and br = broad resonance) and coupling constants *J* are reported in Hertz (Hz). All NMR spectra were processed in MestReNova. HRMS experiments were performed on a hybrid tandem quadrupole/time-of-flight (Q-TOF) instrument, equipped with a pneumatically assisted electrospray (Z-spray) ion source (Micromass, Manchester, UK) operated in the positive mode. The melting points (Mp [°C]) of samples were measured using open capillary tubes and recorded on a Stuart^TM^ melting point apparatus SMP3.

*2-Bromobenzimidazole* (**2**).

Compound **2** was prepared from 2-mercaptobenzimidazole according to a reported procedure [43]. Mp 195−196 °C (*lit*. [44] 191−193 °C, *lit*. [45] 190–192 °C). ^1^H NMR (300 MHz, Methanol-*d_4_*): δ = 7.52 (dd, *J =* 6.0, 3.3 Hz, 2H), 7.29−7.23 (m, 2H).

The experimental data are in accordance with the previously reported data [44,45].

*2-Bromo-1-tertbutoxycarbonylbenzimidazole* (**3**).

Compound **3** was prepared according to a literature procedure [46]. Mp 64−65 °C. ^1^H NMR (300 MHz, CDCl_3_): δ = 7.96−7.92 (m, 1H), 7.72−7.69 (m, 1H), 7.39−7.34 (m, 2H), 1.75 (s, 9H). ^13^C NMR (75 MHz, CDCl_3_): δ = 147.3, 142.7, 133.8, 126.9, 125.1, 124.5, 119.4, 114.7, 86.8, 28.0 (3C). 

Spectroscopic data are in accordance with the previously reported data [46].

### 3.2. General Procedure for the Synthesis of Tert-butyl 2-alkynyl-1H-benzimidazole-1-carboxylate (**4a–o**)

An oven dried 25 mL Schlenk tube equipped with a magnetic stir bar was charged with *tert*-butyl 2-bromobenzimidazole-1-carboxylate **3** (300 mg, 1.01 mmol), PPh_3_ (53 mg, 0.2 mmol), Pd(OAc)_2_ (23 mg, 0.1 mmol), CuI (29 mg, 0.15 mmol) and triethylamine (8 mL). The vial was sealed with a septum-lined pierceable cap, evacuated and backfilled with argon (×3). Then, a solution of alkyne (1.5 mmol, 1.5 equiv.) in 2 mL of Et_3_N was added dropwise to the reaction mixture via a syringe. The reaction mixture was stirred at room temperature for 20 h under an argon atmosphere. After completion of the reaction (monitored by TLC), the solution was filtered through a plug of celite eluting with ethyl acetate (25 mL) and the combined filtrate was dried with MgSO_4_ and concentrated under vacuum. The crude product was directly purified by column chromatography using a mixture petroleum ether/EtOAc as an eluent to give the pure desired products **4a**–**o**.

*2-Hexynyl-1-tertbutoxycarbonylbenzimidazole* (**4a**). 

The crude product was purified by column chromatography on silica gel (PE/EtOAC, 9.8:0.2), followed by recrystallization from Et_2_O to afford compound **4a** as a grey solid (270.7 mg, 90%). Mp 58−59 °C. ^1^H NMR (300 MHz, CDCl_3_): δ = 7.99 (dd, *J* = 6.0, 2.1 Hz, 1H), 7.75 (dd, *J* = 6.0, 2.1 Hz, 1H), 7.40 (td, *J* = 7.2, 1.8 Hz, 1H), 7.37 (td, *J* = 7.2, 1.8 Hz, 1H), 2.55 (t, *J* = 7.2, 3H), 1.73 (s, 9H), 1.73−1.65 (m, 2H), 1.54 (sext, *J* = 7.5 Hz, 2H), 0.97 (t, *J* = 7.2 Hz, 3H). ^13^C NMR (75 MHz, CDCl_3_): δ = 147.9, 142.5, 136.3, 132.0, 125.6, 124.6, 120.0, 114.8, 98.0, 85.6, 72.3, 30.0, 28.1 (3C), 22.1, 19.5, 13.6. HRMS (ESI): *m*/*z* [M+H]^+^ calcd for C_18_H_23_N_2_O_2_: 299.1760; found: 299.1755. 

*2-Phenylethynyl-1-tertbutoxycarbonylbenzimidazole* (**4b**).

The crude product was purified by column chromatography on silica gel (PE/EtOAC, 9.8:0.2), followed by recrystallization from Et_2_O to afford compound **4b** as a beige solid (296 mg, 92%). Mp 108−109 °C. ^1^H NMR (300 MHz, CDCl_3_): δ = 8.04 (dd, *J* = 7.2, 1.8 Hz, 1H), 7.78 (dd, *J* = 7.2, 1.8 Hz, 1H), 7.70−7.67 (m, 2H), 7.57−7.51 (m, 5H), 1.75(s, 9H). ^13^C NMR (75 MHz, CDCl_3_): δ = 147.8, 142.9, 136.1, 132.3, 132.2 (2C), 129.7, 128.5 (2C), 125.9, 124.8, 121.6, 120.3, 114.9, 95.0, 85.9, 80.7, 28.2 (3C). HRMS (ESI): *m*/*z* [M+H]^+^ calcd for C_20_H_19_N_2_O_2_,: 319.1447; found: 319.1442. 

Spectroscopic data are in accordance with the previously reported data [47].

*2-(4-Methoxyphenylethynyl)-1-tertbutoxycarbonylbenzimidazole* (**4c**).

The crude product was purified by column chromatography on silica gel (PE/EtOAC, 9:1) followed by recrystallization from Et2O afforded compound **4c** as a yellow solid (275.08 mg, 78%). Mp 93−94 °C. ^1^H NMR (300 MHz, CDCl_3_): δ = 8.01 (dd, *J* = 6.9, 2.1 Hz, 1H), 7.79 (dd, *J* = 6.9, 2.1 Hz, 1H), 7.64−7.60 (m, 2H), 7.41 (td, *J* = 7.5, 1.8 Hz, 1H), 7.38 (td, *J* = 7.5, 1.8 Hz, 1H), 7.47 (dt, *J* = 9.0, 2.1 Hz, 2H), 3.87 (s, 3H), 1.75 (s, 9H). ^13^C NMR (75 MHz, CDCl_3_): δ = 160.7, 147.9, 142.9, 136.4, 133.9 (2C), 132.2, 125.7, 124.7, 120.1, 114.9, 114.2 (2C), 113.5, 95.6, 85.8, 79.8, 55.4, 28.2 (3C). HRMS (ESI): *m*/*z* [M+H]^+^ calcd for C_21_H_21_N_2_O_3_: 349.1552; found: 349.1547.

*2-(3-Methylphenylethynyl)-1-tertbutoxycarbonylbenzimidazole* (**4d**).

The crude product was purified by column chromatography on silica gel (PE/EtOAC, 9.8:0.2), followed by recrystallization from Et_2_O to afford compound **4d** as a yellow oil (282.63 mg, 84%). ^1^H NMR (300 MHz, CDCl_3_): δ = 8.02 (dd, *J* = 7.8, 1.8 Hz, 1H), 7.78 (dd, *J* = 7.8, 1.8 Hz, 1H), 7.50−7.47 (m, 2H), 7.43 (td, *J* = 7.2, 1.5 Hz, 1H), 7.33−7.24 (m, 2H), 2.40 (s, 3H), 1.75 (s, 9H). ^13^C NMR (75 MHz, CDCl_3_): δ = 147.8, 142.9, 138.2, 136.1, 132.7, 132.2, 130.6, 129.3, 128.4, 125.9, 124.8, 121.3, 120.2, 114.9, 95.3, 85.9, 80.4, 28.2 (3C), 21.3. HRMS (ESI): *m*/*z* [M+H]^+^ calcd for C_21_H_21_N_2_O_2_: 333.1603; found: 333.1597.

*2-(4-Methylphenylethynyl)-1-tertbutoxycarbonylbenzimidazoe* (**4e**). 

The crude product was purified by column chromatography on silica gel (PE/EtOAC, 9.6:0.4), followed by recrystallization from Et_2_O to afford compound **4e** as a white solid (283 mg, 84%). Mp 61−62 °C. ^1^H NMR (300 MHz, CDCl_3_): δ = 8.02 (dd, *J* = 6.9, 2.1 Hz, 1H), 7.76 (dd, *J* = 6.9, 2.1 Hz, 1H), 7.57 (d, *J* = 8.1 Hz, 2H), 7.43−7.37 (m, 2H), 7.22 (d, *J* = 8.1 Hz, 1H), 2.42 (s, 3H), 1.74 (s, 9H). ^13^C NMR (75 MHz, CDCl_3_): δ = 147.9, 142.9, 140.1, 136.2, 132.3, 132.1 (2C), 129.3 (2C), 125.8, 124.3, 102.2, 118.5, 114.9, 95.4, 85.8, 80.2, 28.2 (3C), 21.7. HRMS (ESI): *m*/*z* [M+H]^+^ calcd for C_21_H_21_N_2_O_2_: 333.1603; found: 333.1602. 

*2-(2-Chlorophenylethynyl)-1-tertbutoxycarbonylbenzimidazole* (**4f**).

The crude product was purified by column chromatography on silica gel (PE/EtOAC, 9.8:0.2) followed by recrystallization from Et_2_O afforded compound **4f** as a white solid (270 mg, 76%). Mp 110−111 °C. ^1^H NMR (300 MHz, CDCl_3_): δ = 8.02 (dd, *J* = 7.2, 1.8 Hz, 1H), 7.80 (dd, *J* = 7.2, 1.8 Hz, 1H), 7.70 (dd, *J* = 7.5, 1.8 Hz, 1H), 7.50−7.29 (m, 5H), 1.73 (s, 9H). ^13^C NMR (75 MHz, CDCl_3_): δ = 147.1, 142.9, 136.6, 135.6, 133.9, 132.2, 130.6, 129.6, 126.6, 126.1, 124.9, 121.7, 120.4, 114.9, 91.4, 86.1, 85.0, 28.1 (3C). HRMS (ESI): *m*/*z* [M+H]^+^ calcd for C_20_H_18_ClN_2_O_2_: 353.1057; found: 353.1053. 

*2-(3-Chlorophenylethynyl)-1-tertbutoxycarbonylbenzimidazole* (**4g**). 

The crude product was purified by column chromatography on silica gel (PE/EtOAC, 9.8:0.2), followed by recrystallization from Et_2_O to afford compound **4g** as a white solid (211 mg, 59%). Mp 102−103 °C. ^1^H NMR (300 MHz, CDCl_3_): δ = 8.01 (dd, *J* = 7.2, 1.5 Hz, 1H), 7.47−7.33 (m, 4H), 7.56 (dt, *J* = 7.5, 1.5 Hz, 1H), 1.75 (s, 9H). ^13^C NMR (75 MHz, CDCl_3_): δ = 147.7, 142.9, 135.6, 134.4, 132.2, 131.9, 130.3, 130.0, 129.8, 126.3, 124.9, 123.3, 120.4, 114.9, 93.2, 86.2, 81.7, 28.2 (3C). HRMS (ESI): *m*/*z* [M+H]^+^ calcd for C_20_H_18_ClN_2_O_2_: 353.1057; found: 353.1053.

*2-(4-Chlorophenylethynyl)-1-tertbutoxycarbonylbenzimidazole* (**4h**).

The crude product was purified by column chromatography on silica gel (PE/EtOAC, 9.8:0.2), followed by recrystallization from Et_2_O to afford compound **4h** as a white solid (340 mg, 95%). Mp 130−131 °C. ^1^H NMR (300 MHz, CDCl_3_): δ = 8.02 (dd, *J* = 7.2, 1.8 Hz, 1H), 7.78 (dd, *J* = 7.2, 1.8 Hz, 1H), 7.61 (d, *J* = 8.4 Hz, 2H), 7.49−7.39 (m, 2H), 7.34 (d, *J* = 8.4 Hz, 2H), 1.74 (s, 9H). ^13^C NMR (75 MHz, CDCl_3_): δ = 147.8, 142.9, 135.9, 133.7, 133.4 (2C), 132.2, 129.0 (2C), 126.0, 124.9, 120.3, 120.1, 114.9, 93.7, 86.0, 81.6, 28.2 (3C). HRMS (ESI): *m*/*z* [M+H]^+^ calcd for C_20_H_18_ClN_2_O_2_: 353.1057; found: 353.1053.

*2-(3-Nitrophenylethynyl)-1-tertbutoxycarbonylbenzimidazole* (**4i**).

The crude product was purified by column chromatography on silica gel (PE/EtOAC, 9.6:0.4), followed by recrystallization from Et_2_O to afford compound **4i** as a yellow solid (221 mg, 60%). Mp 152−153 °C. ^1^H NMR (300 MHz, CDCl_3_): δ = 8.53 (t, *J* = 1.8 Hz, 1H), 8.30 (ddd, *J* = 8.1, 2.4, 1.2 Hz, 1H), 8.03−7.95 (m, 2H), 7.81 (d, *J* = 7.2 Hz, 1H), 7.63 (t, *J* = 8.1 Hz, 1H), 7.49−7.41 (m, 2H), 1.77 (s, 9H). ^13^C NMR (75 MHz, CDCl_3_): δ = 148.2, 147.7, 142.9, 137.7, 135.2, 132.2, 129.7, 126.9, 126.4, 125.1, 124.3, 123.4, 120.6, 115.0, 91.8, 86.3, 82.8, 28.2 (3C). HRMS (ESI): *m*/*z* [M+H]^+^ calcd for C_20_H_18_N_3_O_4_: 364.1297; found: 364.1298.

*2-(4-Methoxycarbonyl-phenylethynyl)-1-tertbutoxycarbonylbenzimidazole* (**4j**).

The crude product was purified by column chromatography on silica gel (PE/EtOAC, 9.6:0.4), followed by recrystallization from Et_2_O to afford compound **4j** as a white solid (190.6 mg, 50%). Mp 157−158 °C. ^1^H NMR (300 MHz, CDCl_3_): δ = 8.09 (d, *J* = 8.7, 2H), 8.04−8.01 (m, 1H), 7.80−7.77 (m, 1H), 7.74 (d, *J* = 8.7 Hz, 2H), 7.48−7.38 (m, 2H), 3.96 (s, 3H), 1.75 (s, 9H). ^13^C NMR (75 MHz, CDCl_3_): δ = 166.3, 147.7, 142.9, 135.5, 132.2, 132.1 (2C), 130.8, 129.6 (2C), 126.2, 126.1, 124.9, 120.4, 114.9, 93.7, 86.1, 83.2, 52.3, 28.1 (3C). HRMS (ESI): *m*/*z* [M+H]^+^ calcd for C_22_H_21_N_2_O_4_: 377.1501; found: 377.1503. 

*2-Thiophen-3-ylethynyl-1-tertbutoxycarbonylbenzimidazole* (**4k**).

The crude product was purified by column chromatography on silica gel (PE/EtOAC, 9.8:0.2), followed by recrystallization from Et_2_O to afford compound **4k** as a white solid (178 mg, 54%). Mp 137−138 °C. ^1^H NMR (300 MHz, CDCl_3_): δ = 8.05−8.02 (m, 1H), 7.78−7.74 (m, 2H), 7.43−7.31 (m, 4H), 1.75 (s, 9H). ^13^C NMR (75 MHz, CDCl_3_): δ = 147.8, 142.9, 136.0, 132.2, 131.4, 129.9, 125.9, 125.8, 124.8, 120.7, 120.2, 114.9, 90.4, 85.9, 80.5, 28.2 (3C). HRMS (ESI): *m*/*z* [M+H]^+^ calcd for C_18_H_17_N_2_O_2_S: 325.1011; found: 325.1005. 

*2-(Pyridin-2-ylethynyl)-1-tertbutoxycarbonylbenzimidazole* (**4l**).

The crude product was purified by column chromatography on silica gel (PE/EtOAC, 8:2), followed by recrystallization from Et_2_O to afford compound **4l** as a white solid (204 mg, 63%). Mp 142−143 °C. ^1^H NMR (300 MHz, CDCl_3_): δ = 8.69 (d, *J* = 4.5 Hz, 1H), 8.05 (dd, *J* = 6.9, 1.5 Hz, 1H), 7.81−7.68 (m, 3H), 7.48−7.31 (m, 3H), 1.75 (s, 9H). ^13^C NMR (75 MHz, CDCl_3_): δ = 150.4, 147.7, 142.9, 142.1, 136.1, 135.3, 132.3, 128.2, 126.3, 124.9, 123.8, 120.5, 115.0, 93.2, 86.4, 79.7, 28.1 (3C). HRMS (ESI): *m*/*z* [M+H]^+^ calcd for C_19_H_18_N_3_O_2_: 320.1399; found: 320.1398.

*2-(2-Fluorophenylethynyl)-1-tertbutoxycarbonylbenzimidazole* (**4m**).

The crude product was purified by column chromatography on silica gel (PE/EtOAC, 9.8:0.2), followed by recrystallization from Et_2_O to afford compound **4m** as a yellow solid (283 mg, 84%). Mp 107−108 °C. ^1^H NMR (300 MHz, CDCl_3_): δ = 8.06−8.02 (m, 1H), 7.79 (dd, *J* = 6.9, 1.8 Hz, 1H), 7.67 (td, *J* = 7.5, 1.8 Hz, 1H), 7.47−7.38 (m, 3H), 7.23−7.14 (m, 2H), 1.73 (s, 9H). ^13^C NMR (75 MHz, CDCl_3_): δ = 163.0 (d, *J* = 252.0 Hz), 147.8, 142.9, 135.6, 134.0, 132.3, 131.5 (d, *J* = 7.5 Hz), 126.1, 124.9, 124.2 (d, *J* = 3.5 Hz), 120.4, 115.8 (d, *J* = 20.2 Hz), 114.9, 110.4 (d, *J* = 15.7 Hz), 88.4, 86.2, 85.2 (d, *J* = 3 Hz), 28.0 (3C). ^19^F NMR (282 MHz, CDCl_3_): δ = −107.57. HRMS (ESI): *m*/*z* [M+H]^+^ calcd for C_20_H_18_FN_2_O_2_: 337.1352; found: 337.1346.

*2-(4-Fluorophenylethynyl)-1-tertbutoxycarbonylbenzimidazole* (**4n**).

The crude product was purified by column chromatography on silica gel (PE/EtOAC, 9.8:0.2), followed by recrystallization from Et_2_O to afford compound **4n** as a beige solid (262 mg, 77%). Mp 99−100 °C. ^1^H NMR (300 MHz, CDCl_3_): δ = 8.01 (dd, *J* = 6.9, 2.1 Hz, 1H), 7.78 (dd, *J* = 6.9, 2.1 Hz, 1H), 7.70−7.65 (m, 2H), 7.46−7.37 (m, 2H), 7.15−7.09 (m, 2H), 1.75 (s, 9H). ^13^C NMR (75 MHz, CDCl_3_): δ = 163.4 (d, *J* = 250.5 Hz), 147.8, 142.9, 135.9, 134.3 (d, *J =* 8.2 Hz, 2C), 132.2, 126.0, 124.9, 120.3, 117.7 (d, *J =* 3 Hz), 116.0 (d, *J* = 22.5 Hz, 2C), 114.9, 93.9, 85.9, 80.5 (d, *J* = 1.5 Hz), 28.15 (3C). ^19^F NMR (282 MHz, CDCl_3_): δ = −108.15. HRMS (ESI): *m*/*z* [M+H]^+^ calcd for C_20_H_18_FN_2_O_2_: 337.1352; found: 337.1346. 

*2-Cyclohexylethynyl-1-tertbutoxycarbonylbenzimidazole* (**4o**).

The crude product was purified by column chromatography on silica gel (PE/EtOAC, 9.8:0.2), followed by recrystallization from Et_2_O to afford compound **4o** as a beige solid (243 mg, 74%). Mp 79−80 °C. ^1^H NMR (300 MHz, CDCl_3_): δ = 7.96 (dd, *J* = 6.9, 3.3 Hz, 1H), 7.71 (dd, *J* = 6.9, 2.4 Hz, 1H), 7.41−7.32 (m, 2H), 2.70 (quint, *J* = 5.7 Hz, 1H), 1.99−1.94 (m, 2H), 1.83−1.77 (m, 2H), 1.73 (s, 9H), 1.71−1.59 (m, 3H), 1.45−1.34 (m, 3H). ^13^C NMR (75 MHz, CDCl_3_): δ = 147.9, 142.6, 136.4, 132.1, 125.5, 124.6, 120.0, 114.8, 101.5, 85.6, 72.3, 31.9 (2C), 30.0, 28.1 (3C), 25.7, 24.9 (2C). HRMS (ESI): *m*/*z* [M+H]^+^ calcd for C_20_H_25_N_2_O_2_: 325.1916; found: 325.1911. 

### 3.3. General Procedure for the Synthesis of Benzo[1′,2′:4,5]imidazo[1,2-c][1,3]oxazin-1-one (**5a**–**o**)

To an oven-dried Schlenk tube containing the appropriate *N*-Boc-2-alkynyl benzimidazole (**4a**–**o**) (100 mg, 1 equiv.) in dichloroethane DCE (6 mL), silver carbonate Ag_2_CO_3_ (0.1 equiv.) and trifluoroacetic acid TFA (2 equiv.) were added. The reaction mixture was stirred at 60 °C for 6 h under an argon atmosphere. The progress of the reaction was monitored by TLC. After cooling to room temperature, the mixture was concentrated under vacuum. Then, the residue was dissolved in ethyl acetate and washed with water (2 × 30 mL). The combined organic layers were dried with MgSO_4_ and concentrated under reduced pressure. The crude was purified by column chromatography to give the pure desired benzo[1′,2′: 4,5]imidazo[1,2-*c*][1,3]oxazin-1-one (**5a**–**o**).

*3-Butylbenzo[1′,2′:4,5]imidazo[1,2-c][1,3]oxazin-1-one* (**5a**).

Compound **5a** was prepared according to the general procedure using **4a** (100 mg, 0.34 mmol), Ag_2_CO_3_ (9.2 mg, 0.034 mmol) and TFA (76.50 mg, 0.67 mmol). The crude reaction mixture was purified by column chromatography silica gel (PE/EtOAC, 9:1) to provide the product as a white solid (65.77 mg, 81%). Mp 97−98 °C. (*lit*.^17a^ 92–94 °C). IR (ATR): υ 3056, 2932, 2863, 1755, 1663, 1550, 1447, 1369, 1176, 1094 cm^–1^. ^1^H NMR (300 MHz, CDCl_3_): δ = 8.25 (dd, *J* = 7.2, 1.5 Hz, 1H), 7.81 (dd, *J* = 7.2, 1.8 Hz, 1H), 7.51 (td, *J* = 7.5 Hz, 1.8 Hz, 1H), 7.46 (td, *J* = 7.5, 1.5 Hz, 1H), 6.54 (s, 1H), 2.65 (t, *J* = 7.5 Hz, 2H), 1.76 (quint, *J* = 7.5 Hz, 2H), 1.47 (sext, *J* = 7.2 Hz, 2H), 1 (t, *J* = 7.2 Hz, 3H). ^13^C NMR (75 MHz, CDCl_3_): δ = 162.9, 147.4, 144.1, 129.4, 126.3, 124.9 (2C), 119.7, 114.6, 96.6, 32.8, 28.5, 22.0, 13.7. HRMS (ESI): *m*/*z* [M+H]^+^ calcd for C_14_H_15_N_2_O_2_: 243.1134; found: 243.1128. The experimental data are in accordance with the previously reported data [41].

*3-Phenylbenzo[1′,2′:4,5]imidazo[1,2-c][1,3]oxazin-1-one* (**5b**).

Compound **5b** was prepared according to the general procedure using **4b** (100 mg, 0.31 mmol), Ag_2_CO_3_ (8.67 mg, 0.03 mmol) and TFA (71.83 mg, 0.63 mmol). The crude reaction mixture was purified by column chromatography silica gel (PE/EtOAC, 9:1) to provide the product as a white solid (75.78 mg, 92%). Mp 244−245 °C. IR (ATR): υ 3079, 1760, 1635, 1606, 1543, 1447, 1368, 1171, 1101 cm^–1^. ^1^H NMR (300 MHz, CDCl_3_): δ = 8.30 (dd, *J* = 6.3, 2.1 Hz, 1H), 7.96−7.94 (m, 2H), 7.86 (dd, *J* = 6.3, 2.1 Hz, 1H), 7.57−7.51 (m, 5H), 7.21 (s, 1H). ^13^C NMR (75 MHz, CDCl_3_): δ = 157.5, 147.6, 144.4, 143.5, 131.7, 129.7, 129.5, 129.2 (2C), 126.5, 125.8 (2C), 125.3, 119.8, 114.7, 94.5. HRMS (ESI): *m*/*z* [M+H]^+^ calcd for C_16_H_11_N_2_O_2_: 263.0821; found: 263.0816.

*3-(4-Methoxyphenyl)benzo[1′,2′:4,5]imidazo[1,2-c][1,3]oxazin-1-one* (**5c**).

Compound **5c** was prepared according to the general procedure using **4c** (100 mg, 0.29 mmol), Ag_2_CO_3_ (7.92 mg, 0.029 mmol) and TFA (65.50 mg, 0.57 mmol). The crude reaction mixture was purified by column chromatography silica gel (PE/EtOAC, 8:2) to provide the product as a yellow solid (80 mg, 95%). Mp 225−226 °C. IR (ATR): υ 3041, 2960, 2839, 1751, 1634, 1602, 1506, 1370, 1241, 1181, 1107 cm^–1^. ^1^H NMR (300 MHz, CDCl_3_): δ = 8.29 (dd, *J* = 7.2, 1.5 Hz, 1H), 7.91−7.87 (m, 2H), 7.84 (d, *J* = 9.0 Hz, 1H), 7.54 (td, *J* = 7.2, 1.5 Hz, 1H), 7.49 (t, *J* = 7.2 Hz, 1H), 7.08 (s, 1H), 7.07−7.03 (m, 2H), 3.91 (s, 3H). ^13^C NMR (75 MHz, CDCl_3_): δ = 162.4, 157.4, 148.0, 144.6, 143.7, 134.1, 127.5 (2C), 126.4, 125.0, 122.0, 119.7, 114.6 (3C), 92.6, 55.5. HRMS (ESI): *m*/*z* [M+H]^+^ calcd for C_17_H_13_N_2_O_3_: 293.0926; found: 293.0921.

*3-(3-Methylphenyl)benzo[1′,2′:4,5]imidazo[1,2-c][1,3]oxazin-1-one* (**5d**).

Compound **5d** was prepared according to the general procedure using **4d** (100 mg, 0.30 mmol), Ag_2_CO_3_ (8.3 mg, 0.03 mmol) and TFA (68.7 mg, 0.60 mmol). The crude reaction mixture was purified by column chromatography silica gel (PE/EtOAC, 9:1) to provide the product as a white solid (57.07 mg, 79%). Mp 222−223 °C. IR (ATR): υ 3056, 2922, 1755, 1644, 1549, 1447, 1372, 1278, 1174, 1101 cm^–1^. ^1^H NMR (300 MHz, CDCl_3_): δ = 8.32 (dd, *J* = 6.9, 1.8 Hz, 1H), 7.88 (dd, *J* = 6.9, 1.8 Hz, 1H), 7.75 (d, *J* = 9.3 Hz, 2H), 7.57 (td, *J* = 7.5, 1.5 Hz, 1H), 7.53 (td, *J* = 7.5, 1.5 Hz, 1H), 7.47−7.37 (m, 2H), 7.25 (s, 1H), 2.49 (s, 3H). ^13^C NMR (75 MHz, CDCl_3_): δ = 157.7, 147.7, 144.2, 143.6, 139.1, 132.5, 129.6, 129.4, 129.1, 126.5, 126.3, 125.3, 123.0, 119.8, 114.7, 94.3, 21.5. HRMS (ESI): *m*/*z* [M+H]^+^ calcd for C_17_H_13_N_2_O_2_: 277.0977; found: 277.0971. 

*3-(4-Methylphenyl)benzo[1′,2′:4,5]imidazo[1,2-c][1,3]oxazin-1-one* (**5e**).

Compound **5e** was prepared according to the general procedure using **4e** (100 mg, 0.30 mmol), Ag_2_CO_3_ (8.30 mg, 0.03 mmol) and TFA (68.65 mg, 0.60 mmol). The crude reaction mixture was purified by column chromatography silica gel (PE/EtOAC, 9:1) to provide the product as a white solid (58 mg, 70%). Mp 246−247 °C. IR (ATR): υ 3075, 3023, 2969, 2921, 1766, 1639, 1606, 1547, 1371, 1100 cm^–1^. ^1^H NMR (300 MHz, CDCl_3_): δ = 8.30 (dd, *J* = 6.9, 1.8 Hz, 1H), 7.86−7.85 (m, 1H), 7.82 (d, *J* = 8.1 Hz, 2H), 7.57−7.47 (m, 2H), 7.35 (d, *J* = 8.1 Hz, 2H), 7.14 (s, 1H), 2.46 (s, 3H). ^13^C NMR (75 MHz, CDCl_3_): δ = 157.7, 147.8, 144.4, 143.6, 142.4, 129.9 (2C), 129.4, 126.9, 126.4, 125.7 (2C), 125.1, 119.7, 114.6, 96.6, 21.5. HRMS (ESI): *m*/*z* [M+H]^+^ calcd for C_17_H_13_N_2_O_2_: 277.0977; found: 277.0976.

*3-(2-Chlorophenyl)benzo[1′,2′:4,5]imidazo[1,2-c][1,3]oxazin-1-one* (**5f**).

Compound **5f** was prepared according to the general procedure using **4f** (100 mg, 0.28 mmol), Ag_2_CO_3_ (7.8 mg, 0.028 mmol) and TFA (64.80 mg, 0.57 mmol). The crude reaction mixture was purified by column chromatography silica gel (PE/EtOAC, 9:1) to provide the product as a white solid (43 mg, 51%). Mp 179−180 °C. IR (ATR): υ 3054, 1770, 1642, 1548, 1435, 1367, 1173, 1097 cm^–1^. ^1^H NMR (300 MHz, CDCl_3_): δ = 8.32 (dd, *J* = 6.6, 2.4 Hz, 1H), 7.88 (dd, *J* = 6.9, 1.8 Hz, 1H), 7.82−7.79 (m, 1H), 7.60−7.45 (m, 5H), 7.33 (s, 1H). ^13^C NMR (75 MHz, CDCl_3_): δ = 155.1, 143.7 (2C), 132.8, 132.0, 131.9, 131.2, 130.5, 129.4, 127.4, 127.3, 126.6, 125.6, 120.1, 114.9, 100.8. HRMS (ESI): *m*/*z* [M+H]^+^ calcd for C_16_H_10_ClN_2_O_2_: 297.0431; found: 297.0427. 

*3-(3-Chlorophenyl)benzo[1′,2′:4,5]imidazo[1,2-c][1,3]oxazin-1-one* (**5g**).

Compound **5g** was prepared according to the general procedure using **4g** (100 mg, 0.28 mmol), Ag_2_CO_3_ (7.8 mg, 0.028 mmol) and TFA (64.8 mg, 0.57 mmol). The crude reaction mixture was purified by column chromatography silica gel (PE/EtOAC, 9:1) to provide the product as a white solid (66 mg, 80%). Mp 201−202 °C. IR (ATR): υ 3070, 1768, 1640, 1569, 1367, 1098 cm^–1^. ^1^H NMR (300 MHz, DMSO-*d_6_*): δ = 8.18−8.15 (m, 1H), 8.11 (s, 1H), 8.01 (d, *J* = 6.9 Hz, 1H), 7.91 (s, 1H), 7.86−7.82 (m, 1H), 7.64−7.51 (m, 4H). ^13^C NMR (75 MHz, CDCl_3_): δ = 166.6, 155.8, 135.5, 133.3, 131.6, 131.5, 130.5, 129.4, 126.7, 125.8, 125.6, 123.8, 120.1, 120.0, 114.7, 95.5. HRMS (ESI): *m*/*z* [M+H]^+^ calcd for C_16_H_10_ClN_2_O_2_: 297.0431; found: 297.0428.

*3-(4-Chlorophenyl)benzo[1′,2′:4,5]imidazo[1,2-c][1,3]oxazin-1-one* (**5h**).

Compound **5h** was prepared according to the general procedure using **4h** (100 mg, 0.28 mmol), Ag_2_CO_3_ (7.8 mg, 0.028 mmol) and TFA (64.8 mg, 0.57 mmol). The crude reaction mixture was purified by column chromatography silica gel (PE/EtOAC, 9:1) to provide the product as a white solid (77.34 mg, 92%). Mp 256−257 °C. IR (ATR): υ 3080, 1766, 1640, 1540, 1408, 1113 cm^–1^. ^1^H NMR (300 MHz, CDCl_3_): δ = 8.29 (d, *J* = 7.5 Hz, 1H), 7.87 (d, *J* = 8.7 Hz, 3H), 7.53 (d, *J* = 8.7 Hz, 4H), 7.17 (s, 1H). ^13^C NMR (75 MHz, CDCl_3_): δ = 163.3, 156.2, 147.3, 144.6, 143.3, 137.9, 129.6 (2C), 128.2, 127.0 (2C), 126.6, 125.4, 120.0, 114.6, 94.9. HRMS (ESI): *m*/*z* [M+H]^+^ calcd for C_16_H_10_ClN_2_O_2_: 297.0431; found: 297.0427.

*3-(3-Nitrophenyl)benzo[1′,2′:4,5]imidazo[1,2-c][1,3]oxazin-1-one* (**5i**).

Compound **5i** was prepared according to the general procedure using **4i** (100 mg, 0.28 mmol), Ag_2_CO_3_ (7.6 mg, 0.028 mmol) and TFA (62.8 mg, 0.55 mmol). The crude reaction mixture was purified by column chromatography silica gel (PE/EtOAC, 6:4) to provide the product as a yellow solid (71 mg, 84%). Mp 284−285 °C. IR (ATR): υ 3090, 1749, 1644, 1525, 1367, 1173, 1101 cm^–1^. ^1^H NMR (300 MHz, CDCl_3_): δ = 8.8 (s, 1H), 8.42 (d, *J* = 8.1 Hz, 1H), 8.35−8.33 (m, 1H), 8.26 (d, *J* = 8.1 Hz, 1H), 7.92−7.90 (m, 1H), 7.78 (d, *J* = 7.8 Hz, 1H), 7.58−7.56 (m, 2H), 7.36 (s, 1H). ^13^C NMR (75 MHz, CDCl_3_/TFA-*d_1_*): δ = 162.0, 149.0, 147.0, 139.9, 132.6, 131.7, 131.2, 130.2, 129.2, 129.1, 128.5, 126.8, 122.0, 115.8, 115.7, 91.0. HRMS (ESI): *m*/*z* [M+H]^+^ calcd for C_16_H_9_N_3_O_4_: 308.0666; found: 308.0663.

*3-(4-Methoxycarbonylphenyl)benzo[1′,2′:4,5]imidazo[1,2-c][1,3]oxazin-1-one* (**5j**).

Compound **5j** was prepared according to the general procedure using **4j** (100 mg, 0.27 mmol), Ag_2_CO_3_ (7.3 mg, 0.027 mmol) and TFA (60.6 mg, 0.53 mmol). The crude reaction mixture was purified by column chromatography silica gel (PE/EtOAC, 7:3) to provide the product as a white solid (81 mg, 95%). Mp 262−263 °C.IR (ATR): υ 3080, 2949, 2846, 1763, 1718, 1639, 1542, 1413, 1376, 1270, 1106 cm^–1^. ^1^H NMR (300 MHz, CDCl_3_): δ = 8.31 (d, *J* = 6.6 Hz, 1H), 8.21(d, *J* = 8.1 Hz, 2H), 8.01 (d, *J* = 8.1 Hz, 2H), 7.88 (d, *J* = 6.6 Hz, 1H), 7.59−7.52 (m, 2H), 7.31 (s, 1H), 3.99 (s, 3H). ^13^C NMR (75 MHz, CDCl_3_/ TFA-*d_1_*): δ = 166.6, 163.2, 147.2, 140.3, 134.7, 131.4 (2C), 130.8, 130.0, 128.8, 127.2 (2C), 127.0, 126.8, 115.9, 115.7, 90.7, 53.2. HRMS (ESI): *m*/*z* [M+H]^+^ calcd for C_16_H_10_ClN_2_O_2_: 321.0875; found: 321.0875. 

*3-(2-Thiophenyl)benzo[1′,2′:4,5]imidazo[1,2-c][1,3]oxazin-1-one* (**5k**).

Compound **5k** was prepared according to the general procedure using **4k** (100 mg, 0.31 mmol), Ag_2_CO_3_ (8.5 mg, 0.031 mmol) and TFA (70.4 mg, 0.62 mmol). The crude reaction mixture was purified by column chromatography silica gel (PE/EtOAC, 9:1) to provide the product as a white solid (64.5 mg, 78%). Mp 239−240 °C. IR (ATR): υ 3096, 1760, 1637, 1548, 1368, 1246, 1099 cm^–1^. ^1^H NMR (300 MHz, CDCl_3_): δ = 8.28 (d, *J* = 7.2 Hz, 1H), 8.03 (s, 1H), 7.83 (d, *J* = 7.8 Hz, 1H), 7.56−7.47 (m, 4H), 7.0 (s, 1H). ^13^C NMR (75 MHz, CDCl_3_): δ = 153.7, 147.8, 144.6, 132.0, 129.6, 127.8, 126.6, 126.4, 125.2, 124.0, 124.4, 119.8, 114.6, 94.0. HRMS (ESI): *m*/*z* [M+H]^+^ calcd for C_14_H_9_N_2_O_2_S: 269.0385; found: 269.0379. 

*3-Pyridin-2-ylbenzo[1′,2′:4,5]imidazo[1,2-c][1,3]oxazin-1-one* (**5l**).

Compound **5l** was prepared according to the general procedure using **4l** (100 mg, 0.31 mmol), Ag_2_CO_3_ (8.6 mg, 0.031 mmol) and TFA (71.50 mg, 0.63 mmol). The crude reaction mixture was purified by column chromatography silica gel (PE/EtOAC, 6:4) to provide the product as a white solid (59 mg, 71%). Mp 240−241 °C. IR (ATR): υ 3060, 1762, 1644, 1575, 1464, 1364, 1171, 1101 cm^–1^. ^1^H NMR (300 MHz, CDCl_3_): δ = 8.76 (d, *J* = 3.9 Hz, 1H), 8.31 (dd, *J* = 6.6, 1.2 Hz, 1H), 8.07 (d, *J* = 7.8 Hz, 1H), 7.94−7.88 (m, 3H), 7.58−7.51 (m, 1H), 7.54 (s, 1H), 7.44 (ddd, *J* = 7.8, 4.8, 1.2 Hz). ^13^C NMR (75 MHz, CDCl_3_): δ = 155.8, 150.3, 147.6, 147.3, 144.7, 143.4, 137.2, 129.5, 126.5, 125.5, 125.4, 120.5, 120.2, 114.7, 96.7. HRMS (ESI): *m*/*z* [M+H]^+^ calcd for C_15_H_10_N_3_O_2_: 264.0773; found: 264.0771. 

*3-(2-Fluorophenyl)benzo[1′,2′:4,5]imidazo[1,2-c][1,3]oxazin-1-one* (**5m**).

Compound **5m** was prepared according to the general procedure using **4m** (100 mg, 0.30 mmol), Ag_2_CO_3_ (8.2 mg, 0.030 mmol) and TFA (67.8 mg, 0.6 mmol). The crude reaction mixture was purified by column chromatography silica gel (PE/EtOAC, 9:1) to provide the product as a yellow solid (73.32 mg, 88%). Mp 180−181 °C. IR (ATR): υ 3090, 1767, 1631, 1539, 1445, 1369, 1217, 1036 cm^–1^. ^1^H NMR (300 MHz, CDCl_3_): δ = 8.30 (dd, *J* = 6.0, 2.1 Hz, 1H), 8.05 (td, *J* = 7.8, 1.5 Hz, 1H), 7.87 (dd, *J* = 6.0, 1.8 Hz, 1H), 7.58−7.52 (m, 3H), 7.49 (s, 1H), 7.36 (td, *J* = 7.8, 0.9 Hz, 1H), 7.31−7.24 (m, 1H). ^13^C NMR (75 MHz, CDCl_3_): δ = 160.5 (d, *J* = 253.5 Hz), 151.8, 147.4, 144.6, 143.3, 132.8 (d, *J* = 9.0 Hz), 129.4, 128.3, 126.5, 125.42, 124.9 (d, *J* = 3.7 Hz), 120.0, 118.2 (d, *J* = 9.7 Hz), 116.8 (d, *J* = 22.5 Hz), 114.7, 99.8 (d, *J* = 17.2 Hz). ^19^F NMR (282 MHz, CDCl_3_): δ = −110.17. HRMS (ESI): *m*/*z* [M+H]^+^ calcd for C_16_H_10_FN_2_O_2_: 281.0726; found: 281.0722. 

*3-(4-Fluorophenyl)benzo[1′,2′:4,5]imidazo[1,2-c][1,3]oxazin-1-one* (**5n**).

Compound **5n** was prepared according to the general procedure using **4n** (100 mg, 0.30 mmol), Ag_2_CO_3_ (8.2 mg, 0.03 mmol) and TFA (67.80 mg, 0.6 mmol). The crude reaction mixture was purified by column chromatography silica gel (PE/EtOAC, 9:1) to provide the product as a white solid (57 mg, 68%). Mp 239−240 °C. IR (ATR): υ 3089, 3022, 1770, 1635, 1601, 1508, 1449, 1238, 1101 cm^–1^. ^1^H NMR (300 MHz, CDCl_3_): δ = 8.30 (dd, *J* = 6.0, 1.8 Hz, 1H), 7.97−7.93 (m, 2H), 7.86 (dd, *J* = 6.0, 1.8 Hz, 1H), 7.56 (td, *J* = 7.2, 1.5 Hz, 1H), 7.52 (td, *J* = 7.5, 1.5 Hz, 1H), 7.29−7.23 (m, 2H), 7.17 (s, 1H). ^13^C NMR (75 MHz, CDCl_3_): δ = 164.2 (d, *J* = 249.0 Hz), 155.9, 148.8, 144.7, 144.0, 129.8, 128.7 (d, *J* = 8.2 Hz, 2C), 127.1 (d, *J* = 3 Hz), 126.4, 125.1, 119.9, 116.8 (d, *J* = 21.7 Hz, 2C), 114.5, 95.4. ^19^F NMR (282 MHz, CDCl_3_): δ = −106.68. HRMS (ESI): *m*/*z* [M+H]^+^ calcd for C_16_H_10_FN_2_O_2_: 281.0726; found: 281.0722.

*3-Cyclohexylbenzo[1′,2′:4,5]imidazo[1,2-c][1,3]oxazin-1-one* (**5o**).

Compound **5o** was prepared according to the general procedure using **4o** (100 mg, 0.31 mmol), Ag_2_CO_3_ (8.5 mg, 0.031 mmol) and TFA (70.3 mg, 0.62 mmol). The crude reaction mixture was purified by column chromatography silica gel (PE/EtOAC, 9:1) to provide the product as a white solid (74.43 mg, 90%). Mp 160−161 °C. IR (ATR): υ 3090, 2925, 2855, 1765, 1665, 1554, 1449, 1366, 1155, 1088 cm^–1^. ^1^H NMR (300 MHz, CDCl_3_): δ = 8.26 (d, *J* = 7.5 Hz, 1H), 7.82 (d, *J* = 7.2 Hz, 1H), 7.51 (td, *J* = 7.5, 1.5 Hz, 1H), 7.46 (t, *J* = 7.2 Hz, 1H), 6.54 (s, 1H), 2.60−2.51 (m, 1H), 2.12−2.08 (m, 2H), 1.94−1.90 (m, 2H), 1.82−1.78 (m, 1H), 1.57−1.27 (m, 5H). ^13^C NMR (75 MHz, CDCl_3_): δ = 166.7, 147.7, 144.2, 129.5, 126.2, 124.9 (2C), 119.7, 114.6, 94.8, 41.5, 30.1 (2C), 25.7 (2C), 25.6. HRMS (ESI): *m*/*z* [M+H]^+^ calcd for C_16_H_17_N_2_O_2_: 269.1290; found: 269.1286.

### 3.4. Details of DFT Calculations

The structure of each studied species was optimized by using the Turbomole 7.4 program package [48]. Before their visualization using TmoleX (version 4.5.3), the structure of each individual species was optimized in the gas phase, with a convergence criterion of 10^−8^ Hartree, using the hybrid functional B3LYP and the triplet-ζ basis set 6-311 + G* [49] to collect its more stable 3D conformer. The stability of each structure was then investigated during further DFT calculations. During this step, the energy of each species was then minimized again using DFT calculations combining the Resolution of Identity (RI) approximation [50,51], within the Turbomole 7.4 program package using the B3LYP function with the def2-TZVP basis set [52,53,54]. All minimum energy structures were obtained with full optimization, without constraints. Corrections for long-range non-bonding interactions were given using the Grimme D3 dispersion model [54,55]. An implicit solvent model was additionally undertaken, using the COSMO Model implemented in Turbomole to determine thermodynamic and charge population properties in DCE. Analytical frequencies were then run on each structure at 1 atm and 298.15 K to finally calculate each energy.

## 4. Conclusions

In conclusions, we have reported an efficient and general access to 1*H*-benzo[4,5]imidazo[1,2-*c*][1,3]oxazin-1-ones, involving an intramolecular deprotective heterocyclization sequence catalyzed by a combination between Ag_2_CO_3_ and TFA in dichloroethane at 60 °C. While this procedure is compatible with a wide range of aliphatic, aromatic and heteroaromatic alkynes, ZnCl_2_-mediated heterocyclization showed a limitation when the starting alkyne was aromatic. In all experiments, no trace of 5-*exo*-*dig* heterocycles was observed, since only the 6-*endo*-*dig* products were obtained exclusively in good to excellent yields, proving the high selectivity of this silver-catalyzed oxacyclization. In addition, a computational study was performed in order to rationalize the mechanism of 6-*endo*-*dig* oxacyclization and it was found that experimental results are in a good agreement with the theoretical calculation. The synthetic potential of our catalytic system (Ag_2_CO_3_/TFA) to promote intramolecular 6-*endo*-*dig* cyclization showed that *N*-Boc-2-alkynyl-imidazole and *N*-Boc-2-alkynyl-benzimidazole substrates are interesting for the synthesis of other new polycyclic heterocycles.

## Figures and Tables

**Table 1:** Optimization of oxacyclization reaction conditions.

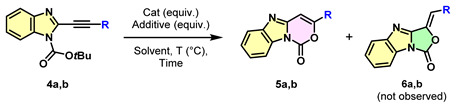							
Entry	R	Solvent	Catalyst (Equiv.)	Additive (Equiv.)	T (°C)	Time (h)	Ratio (%) ^a^ 4/5
1	*n*-Bu	DCM	ZnCl_2_ (1.5) [41]	-	40	3	0/100 **^b^**
2	Ph	DCM	ZnCl_2_ (1.5)	-	40	48	60/40
3	Ph	DCM	ZnCl_2_ (1.5)	-	60	48	50/50
4	Ph	DCM	Ag_2_CO_3_ (0.1)	TFA (2)	60	24	15/85
**5**	**Ph**	**DCE**	**Ag_2_CO_3_ (0.1)** [40]	**TFA (2)**	**60**	**6**	**0/100 ^c^**
6	Ph	DCE	Ag_2_CO_3_ (0.1)	TFA (2)	40	24	5/95

^a^ The ratio of mixture (**4**/**5**) was calculated from the crude 1H NMR spectrum. ^b^ Compound **5a** was isolated in 80% yield after purification by silica-gel column chromatography. ^c^ Compound **5b** was isolated in 90% yield after purification by silica-gel column chromatography.

**Table 2:** NPA charges for the alkyne carbons C_α_ and C_β_ of compounds **4a**–**o**.

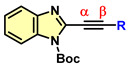					
Entry	Comp.	Charge	Entry	Comp.	Charge
1	4a	α = −0.100 β = +0.125	9	4i	α = −0.012 β = +0.064
2	4b	α = −0.041 β = +0.078	10	4j	α = −0.020 β = +0.069
3	4c	α = −0.057 β = +0.082	11	4k	α = −0.018 β = +0.038
4	4d	α = −0.045 β = +0.081	12	4l	α = −0.017 β = +0.059
5	4e	α = −0.049 β = +0.080	13	4m	α = −0.023 β = +0.070
6	4f	α = −0.018 β = +0.071	14	4n	α = −0.038 β = +0.075
7	4g	α = −0.026 β = +0.070	15	4o	α = −0.095 β = +0.130
8	4h	α = −0.032 β = +0.073	--	--	--

## Data Availability

The data set presented in this study is available in this article.

## Supplementary Materials

The following supporting information can be downloaded at: https://www.mdpi.com/article/10.3390/molecules28052403/s1, Section S1. Copies of ^1^H, ^13^C and ^19^F NMR spectra, Section S2. Details of DFT calculations, Section S3. References.

## Sample Availability

Samples of the compounds **1**–**3**, **4a**–**o** and **6a**–**o** are available from the authors.
