# Supplementary material for: Efficient Synthesis of 1H-Benzo[4,5]imidazo[1,2-c][1,3]oxazin-1-one Derivatives Using Ag2CO3/TFA-Catalyzed 6-endo-dig Cyclization: Reaction Scope and Mechanistic Study
Source: Molecules. 2023 Mar 6;28(5):2403. doi: 10.3390/molecules28052403 (PMC10005794; doi:10.3390/molecules28052403)
Supplement: Supplementary file 1 [file molecules-28-02403-s001.zip › molecules-2241820-supplementary.pdf]

Supporting information for

Efficient Synthesis of 1*H*-Benzo[4,5]imidazo[1,2-*c*][1,3]oxazin-1-one Derivatives Using Ag_2CO_3/TFA-Catalyzed 6-*endo-dig* Cyclization: Reaction Scope and Mechanistic study.

Abdelkarim El Qami [1,2], Badr Jismy [1,*], Mohamed Akssira [2], Johan Jacquemin [3], Abdellatif Tikad [4,*] and Mohamed Abarbri 2,*

1 Laboratoire de Physico-Chimie des Matériaux et des Electrolytes pour l'Energie (PCM2E), EA 6299, Faculté des Sciences et Techniques, Université de Tours. Avenue Monge Faculté des Sciences, Parc de Grandmont, 37200 Tours, France; abdelkarimelqami@gmail.com

2 Laboratoire de Chimie Physique et Biotechnologies des Biomolécules et des Matériaux (LCP2BM), FSTM, Université Hassan II de Casablanca, BP 146, Mohammedia 28800, Morocco; akssira.m@gmail.com

3 Materials Science and Nano-Engineering (MSN) Department, Mohammed VI Polytechnic University (UM6P), Lot 660–Hay Moulay Rachid, Benguerir 43150, Morocco; johan.jacquemin@um6p.ma

4 Laboratoire de Chimie Moléculaire et Substances Naturelles. Université Moulay Ismail, Faculté des Sciences. B.P. 11201, Zitoune, Meknès 50050, Morocco

* Correspondence: badr.jismy@hotmail.com (B.J.); a.tikad@umi.ac.ma or abdel.tikad@gmail.com(A.T.);

mohamed.abarbri@univ-tours.fr (M.A.)

Table of Contents

1. Copies of ^{1}H, ^{13}C and ^{19}F NMR spectra S2-S33

2. Details of DFT calculations S34-S58

3. References S59

^{1}H NMR (300 MHz, $CDCl_3$) of **4a**

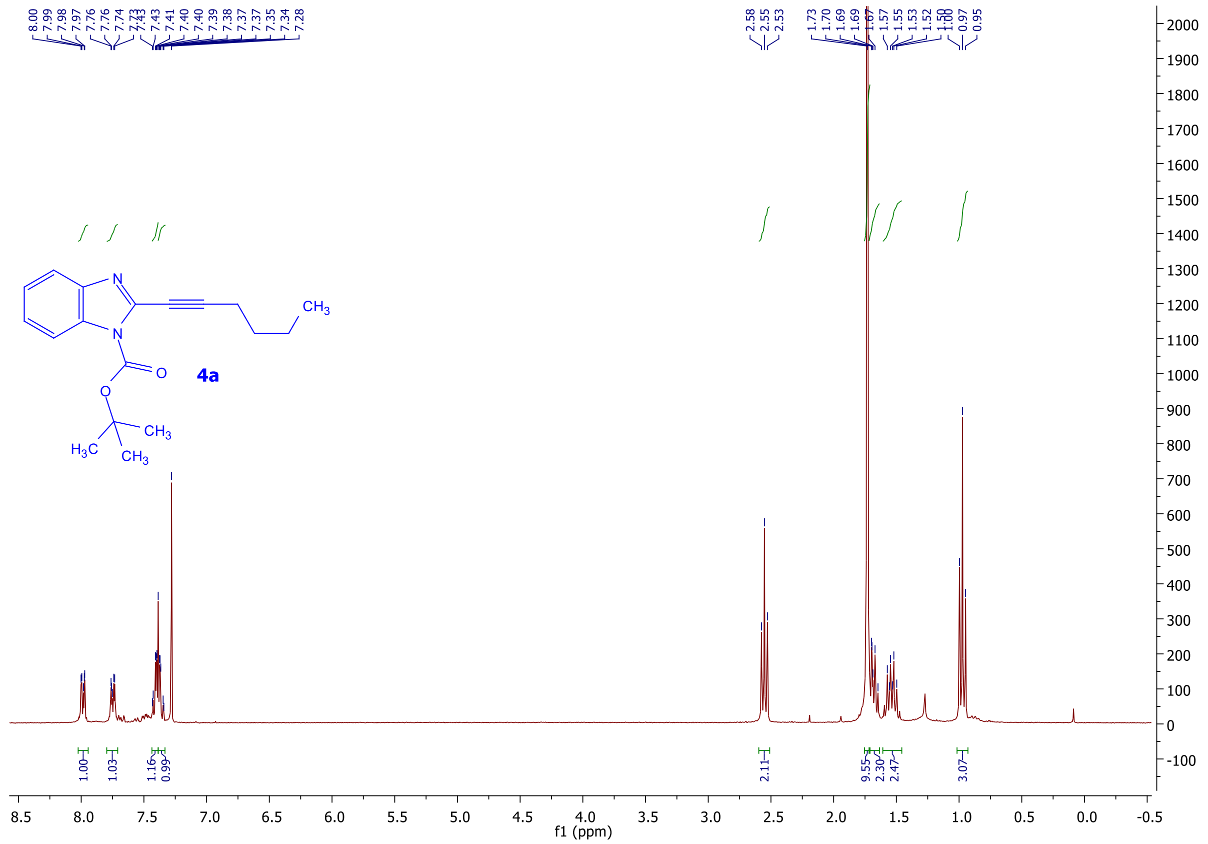

^{13}C NMR (75 MHz, $CDCl_3$) of **4a**

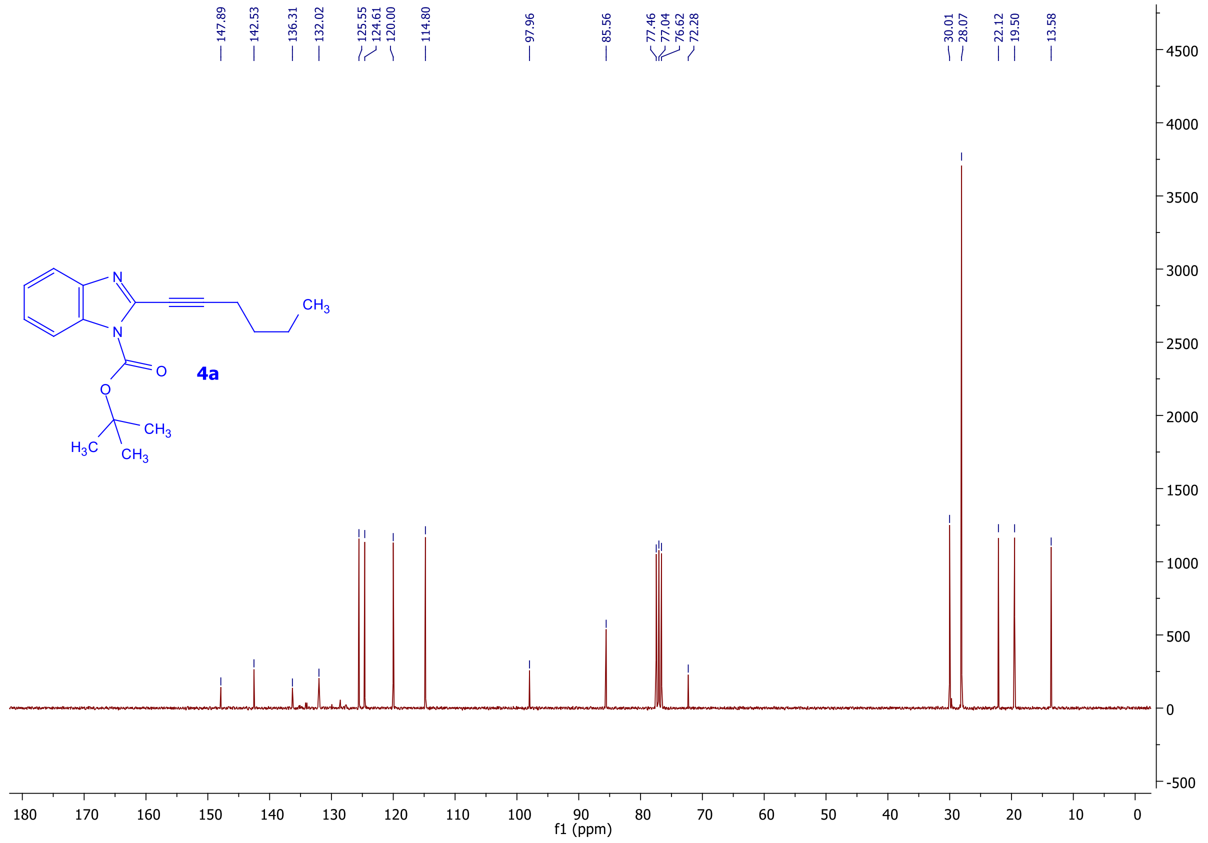

^{1}H NMR (300 MHz, $CDCl_3$) of **4b**

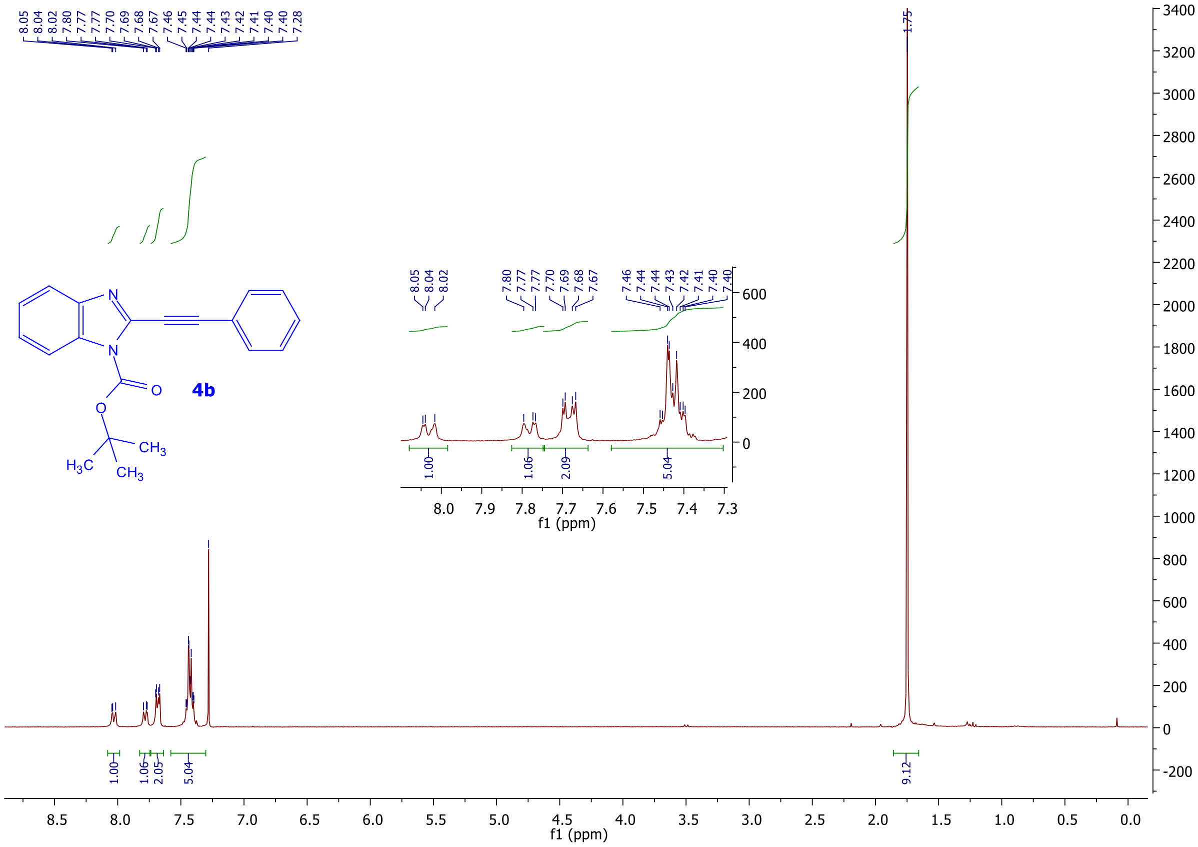

^{13}C NMR (75 MHz, $CDCl_3$) of **4b**

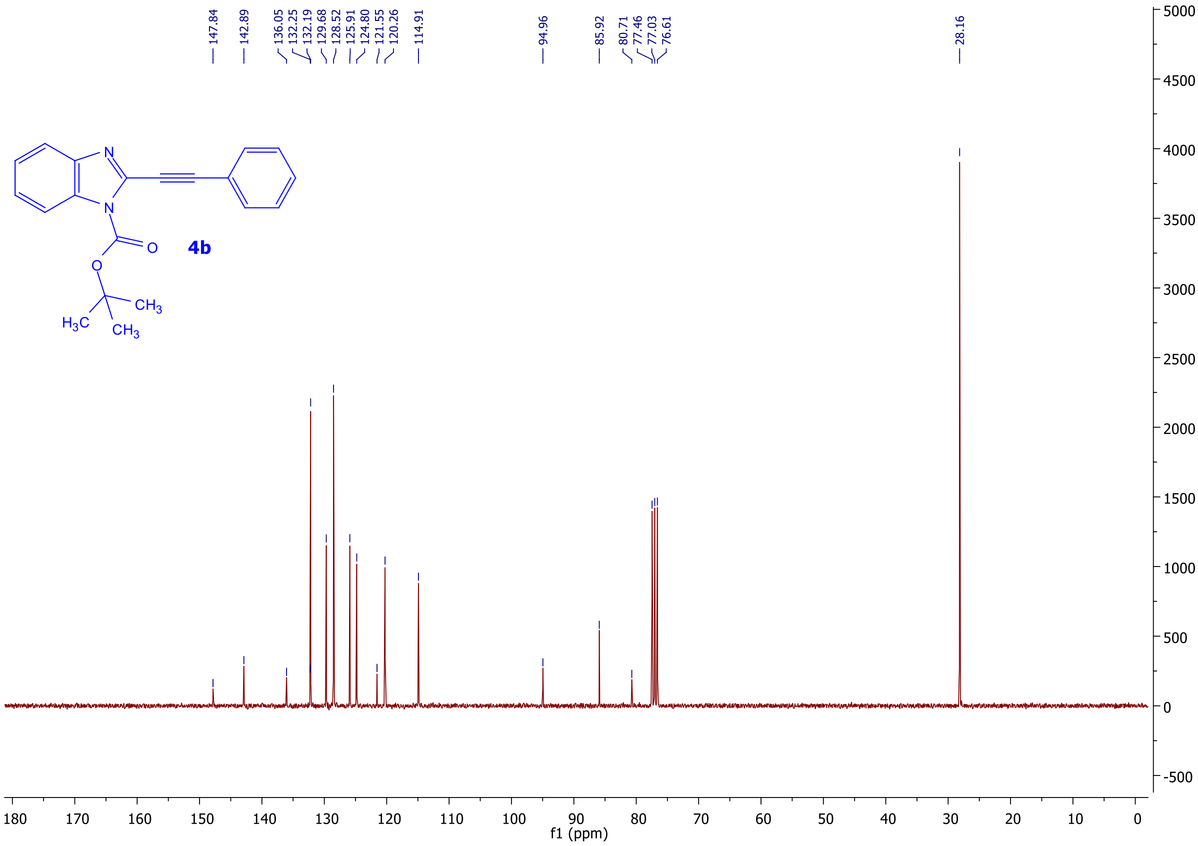

^{1}H NMR (300 MHz, $CDCl_3$) of **4c**

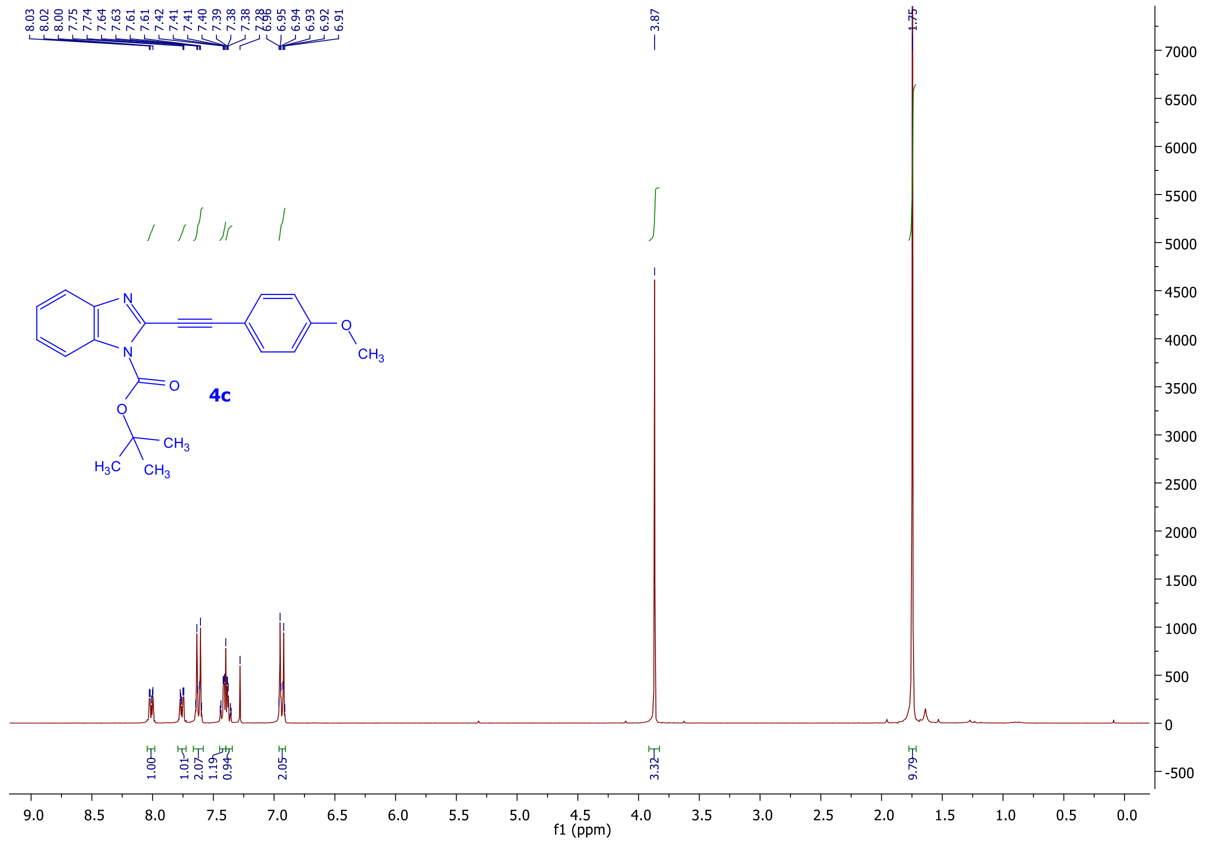

^{13}C NMR (75 MHz, $CDCl_3$) of **4c**

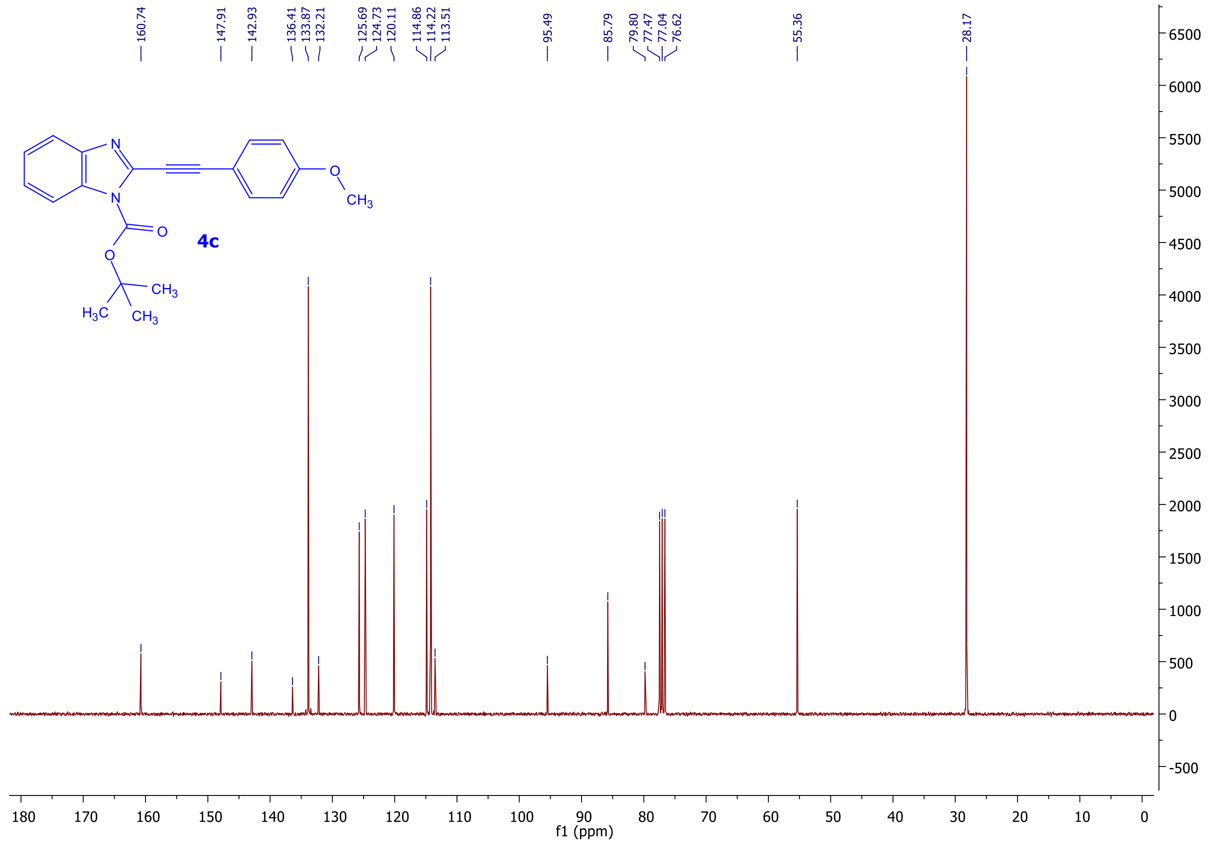

^{1}H NMR (300 MHz, $CDCl_3$) of **4d**

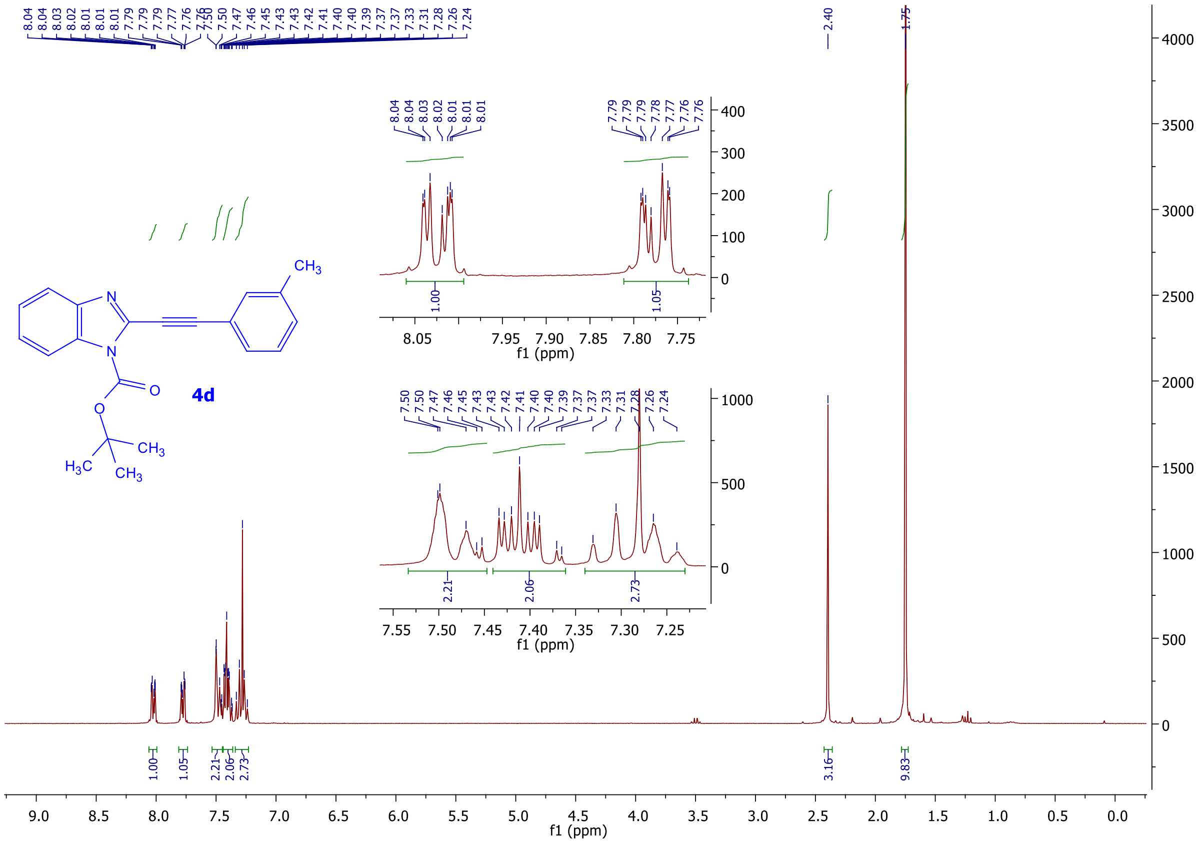

^{13}C NMR (75 MHz, $CDCl_3$) of **4d**

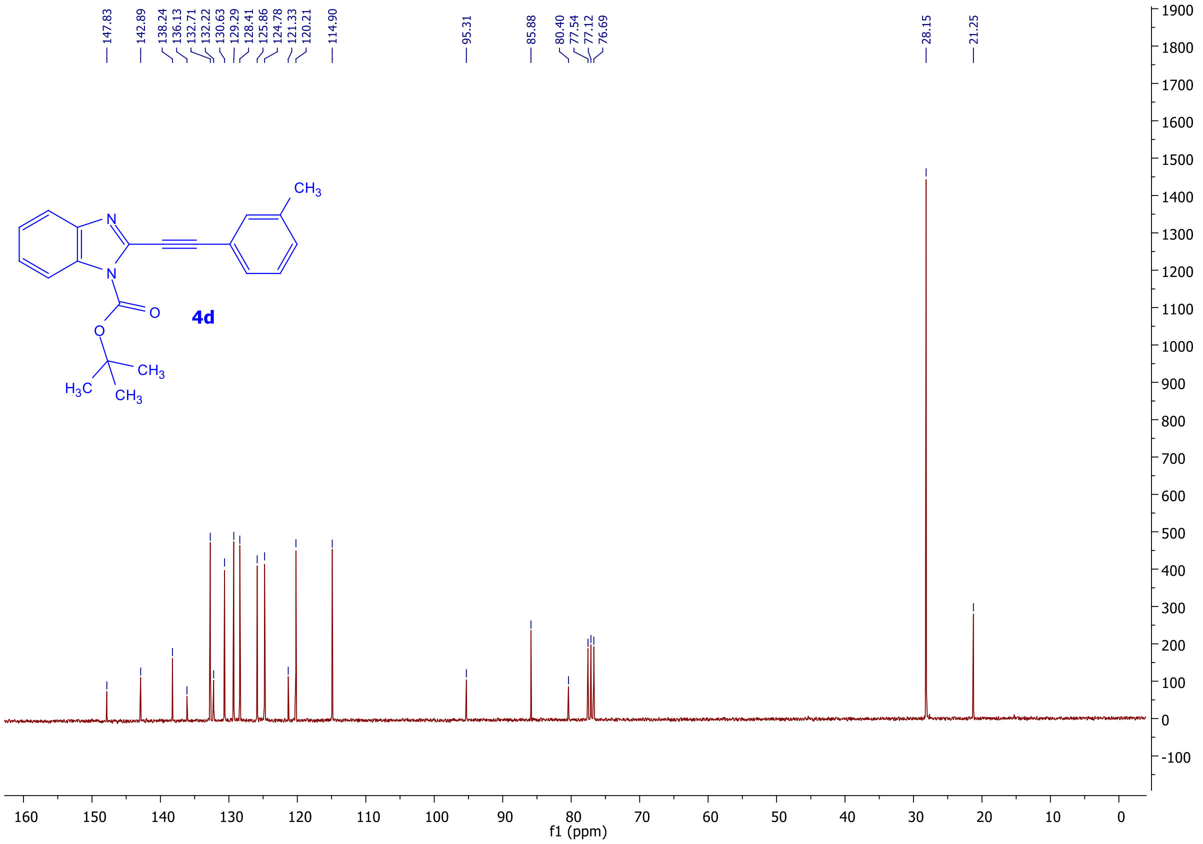

^{1}H NMR (300 MHz, $CDCl_3$) of **4e**

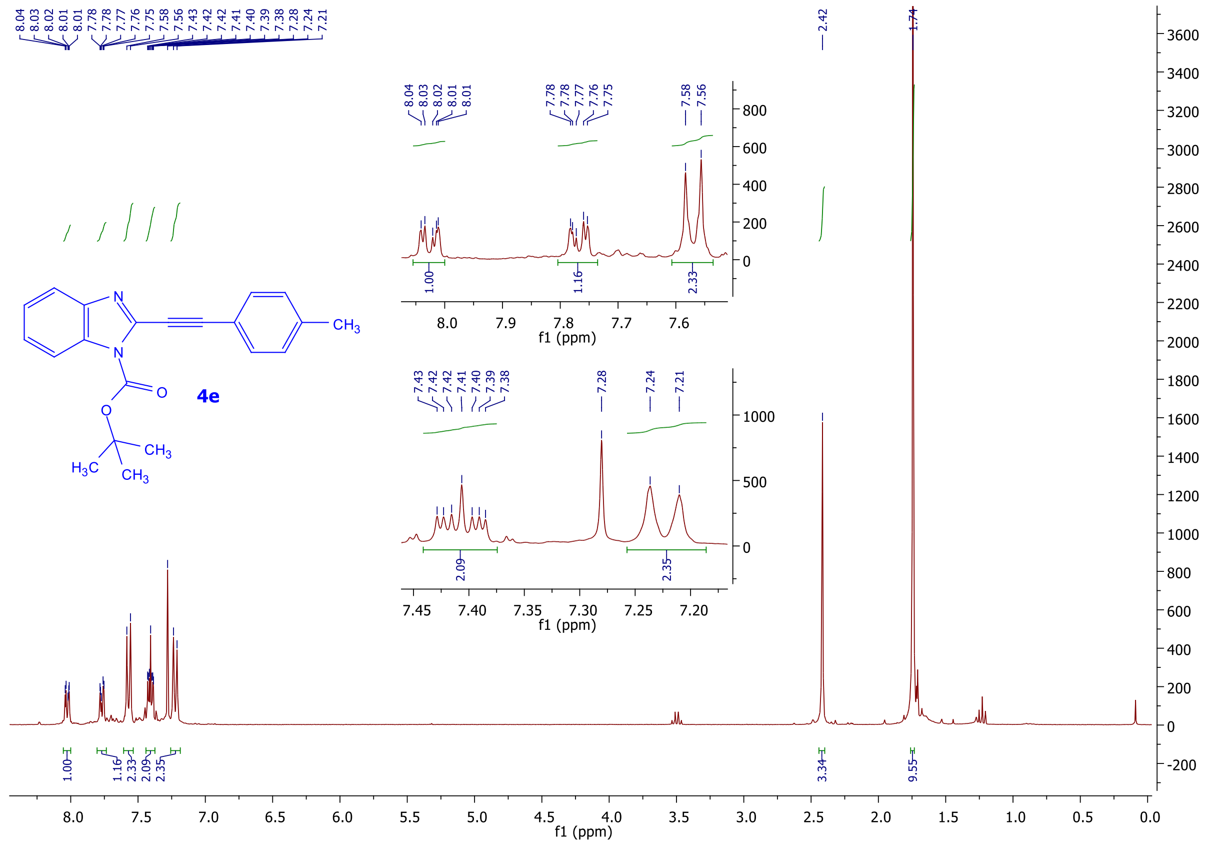

^{13}C NMR (75 MHz, $CDCl_3$) of **4e**

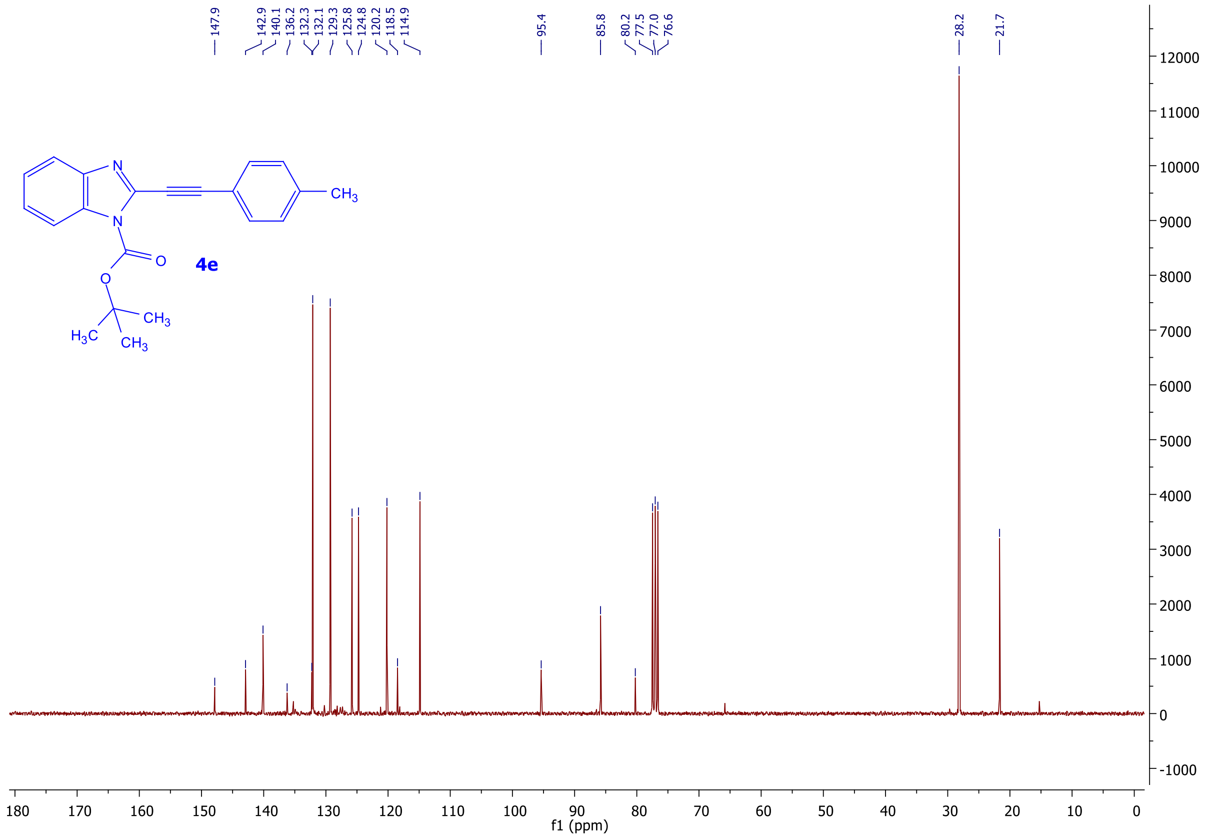

^{1}H NMR (300 MHz, $CDCl_3$) of **4f**

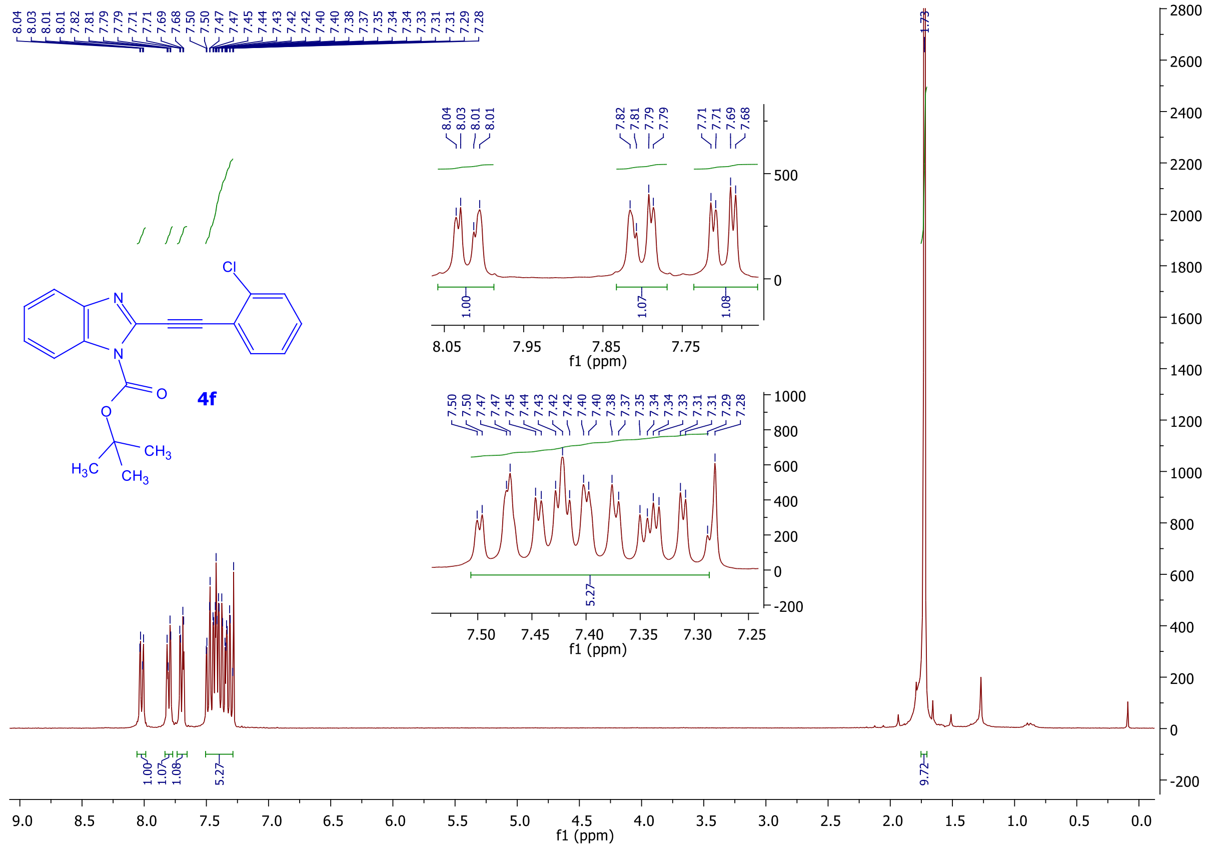

^{13}C NMR (75 MHz, $CDCl_3$) of **4f**

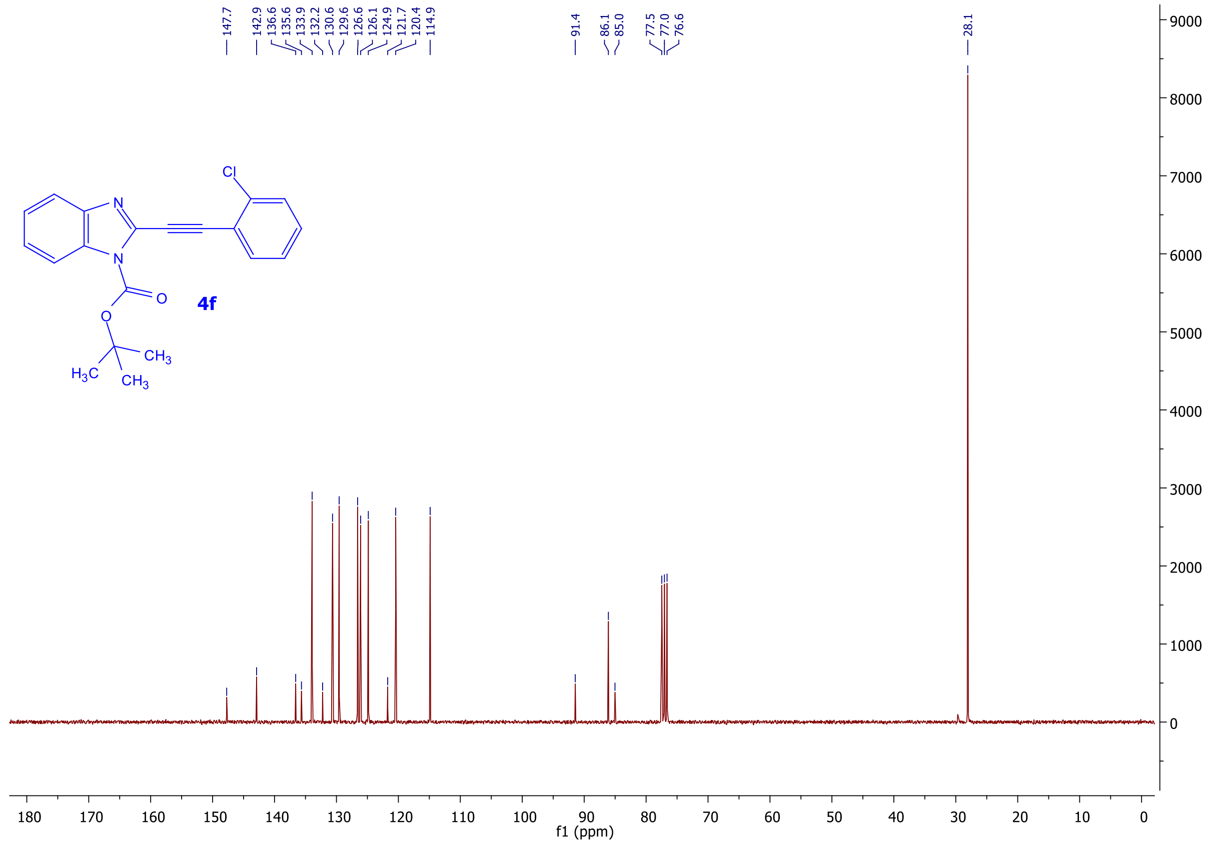

^{1}H NMR (300 MHz, $CDCl_3$) of **4g**

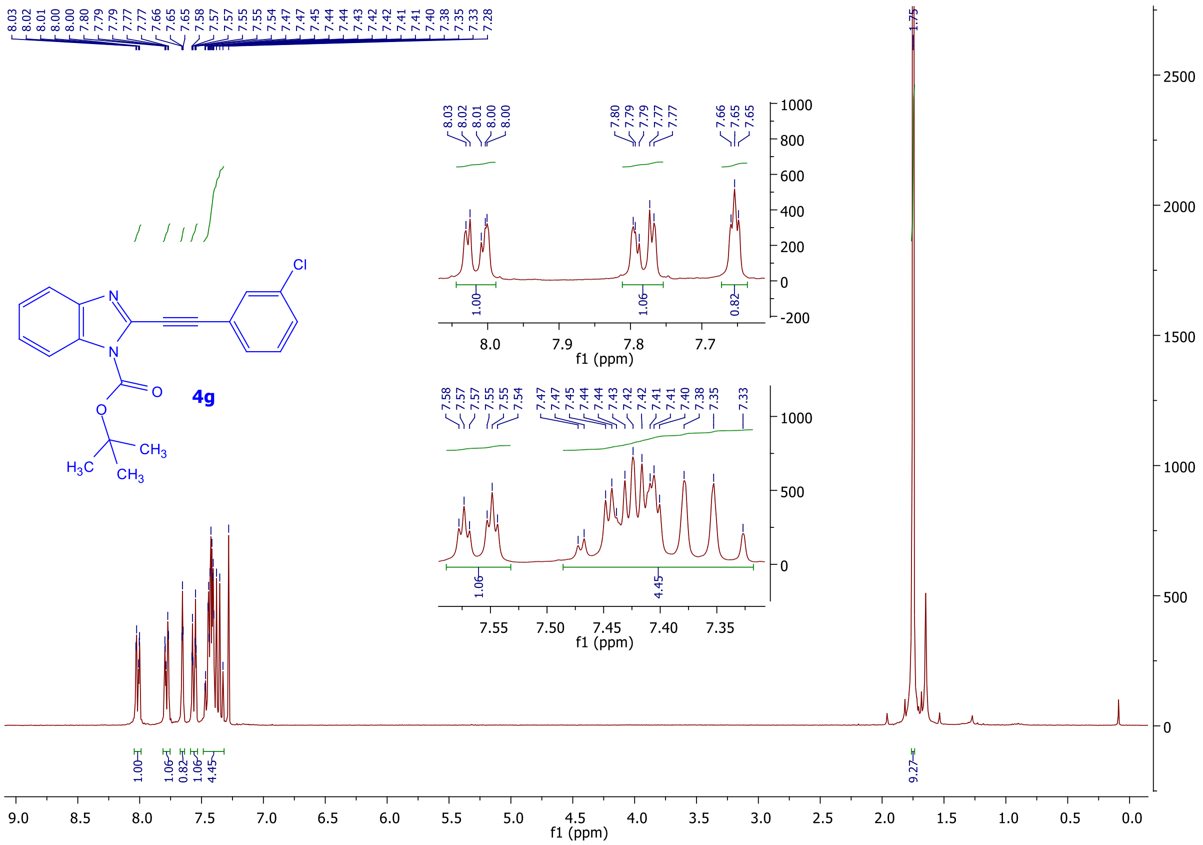

^{13}C NMR (75 MHz, $CDCl_3$) of **4g**

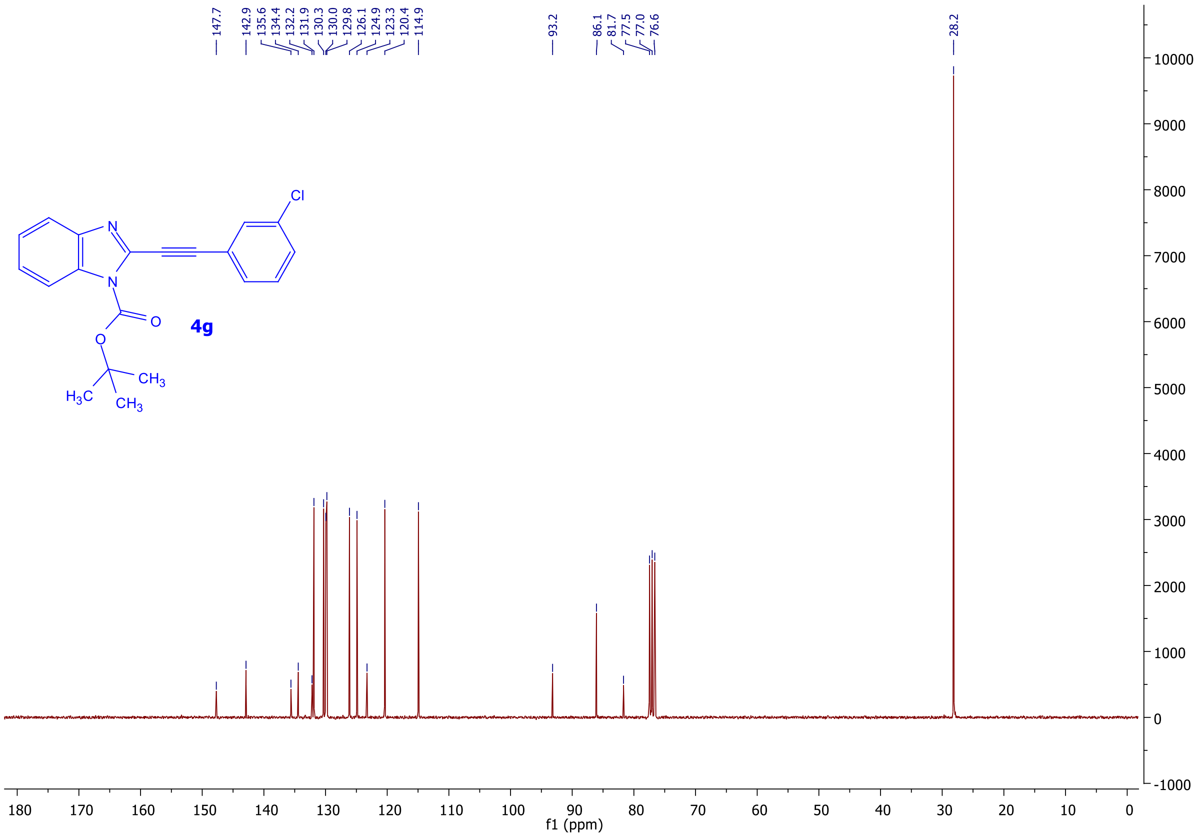

^{1}H NMR (300 MHz, $CDCl_3$) of **4h**

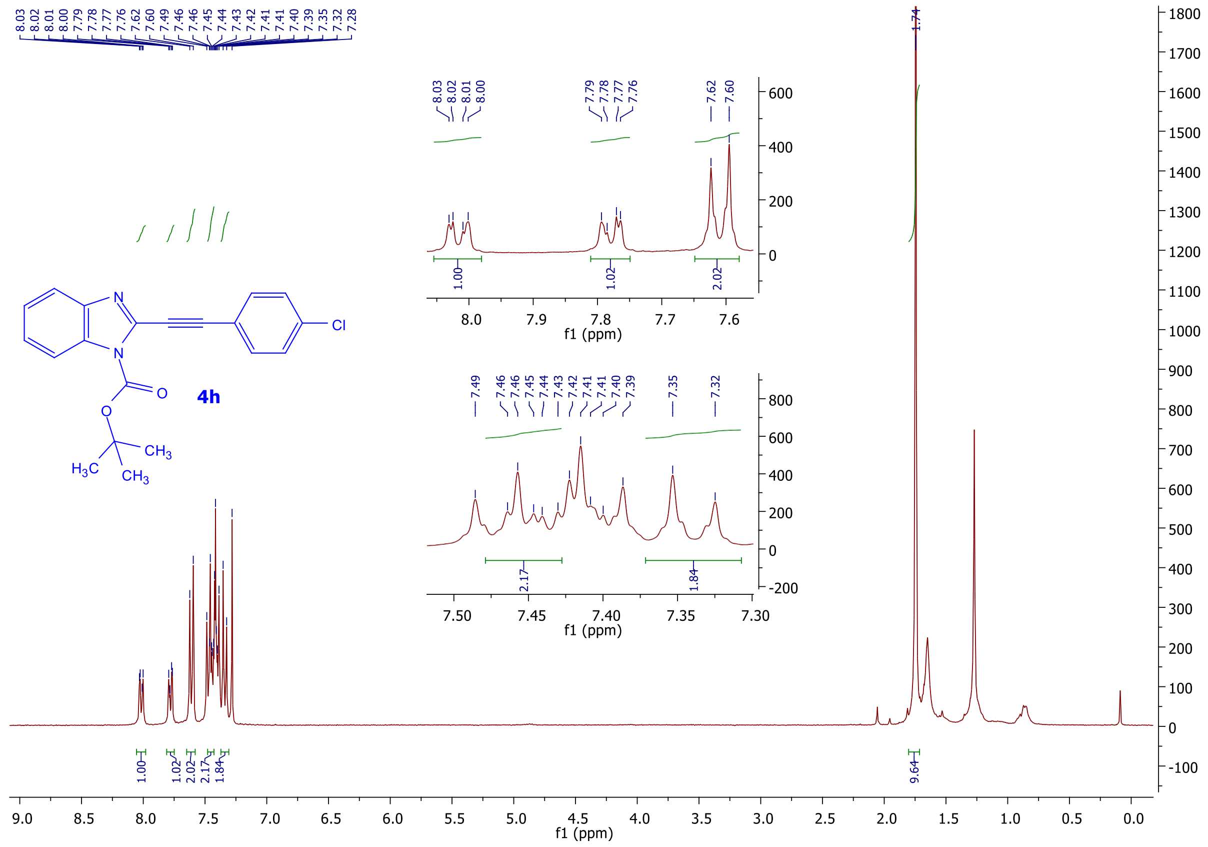

^{13}C NMR (75 MHz, $CDCl_3$) of **4h**

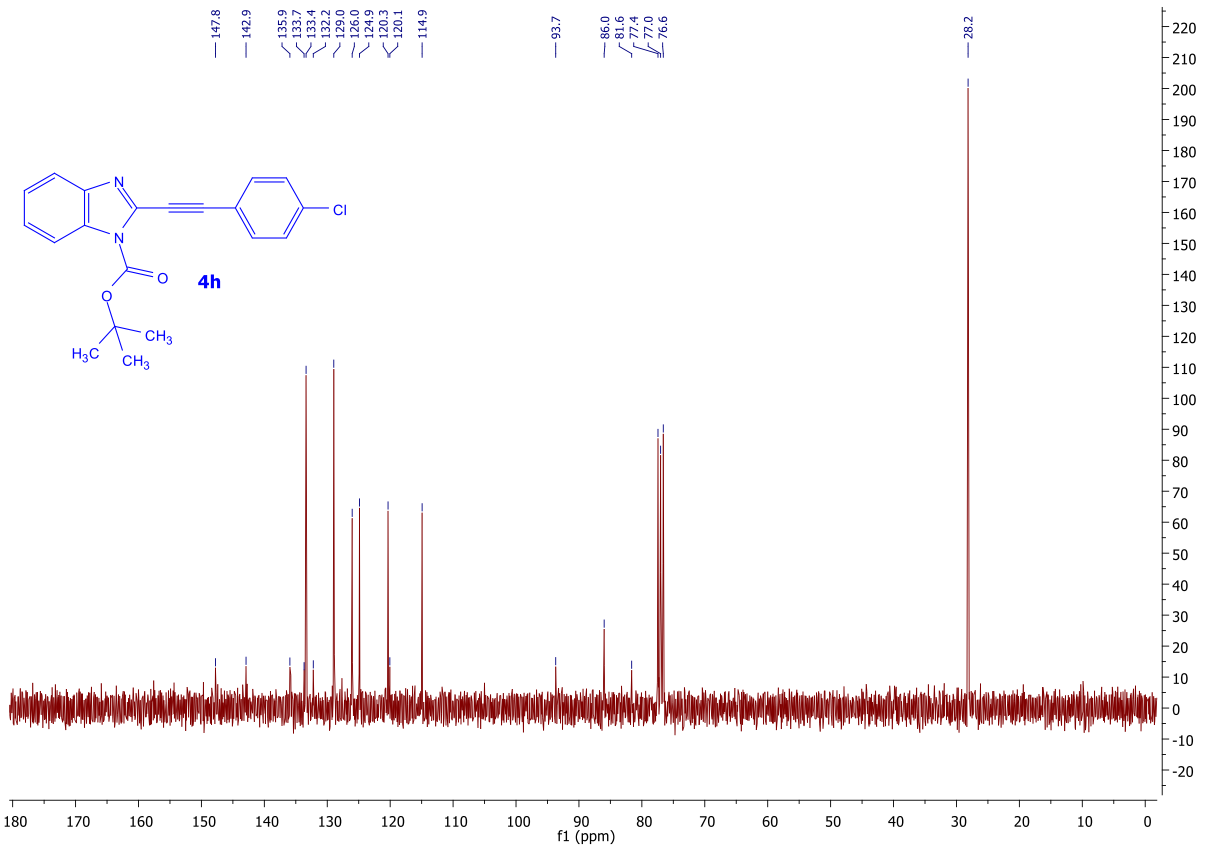

^{1}H NMR (300 MHz, $CDCl_3$) of **4i**

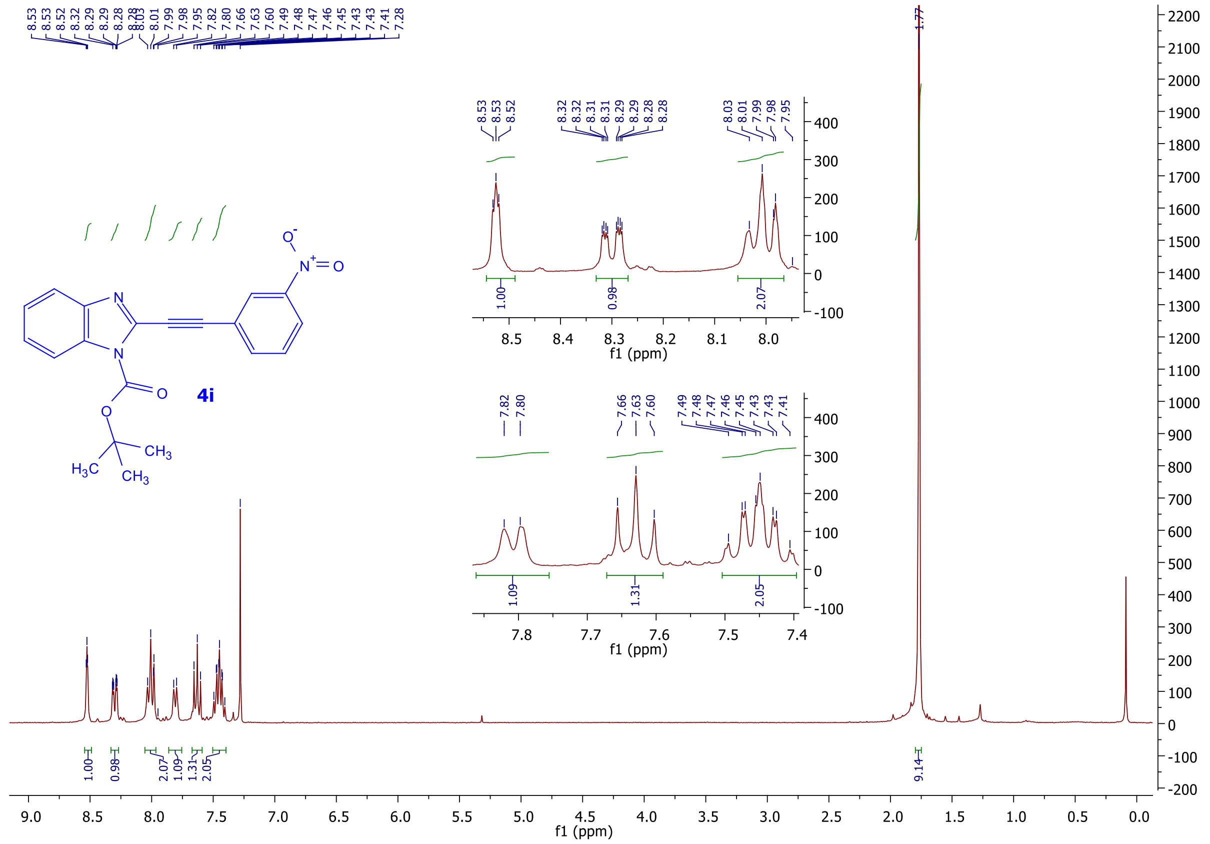

^{13}C NMR (75 MHz, $CDCl_3$) of **4i**

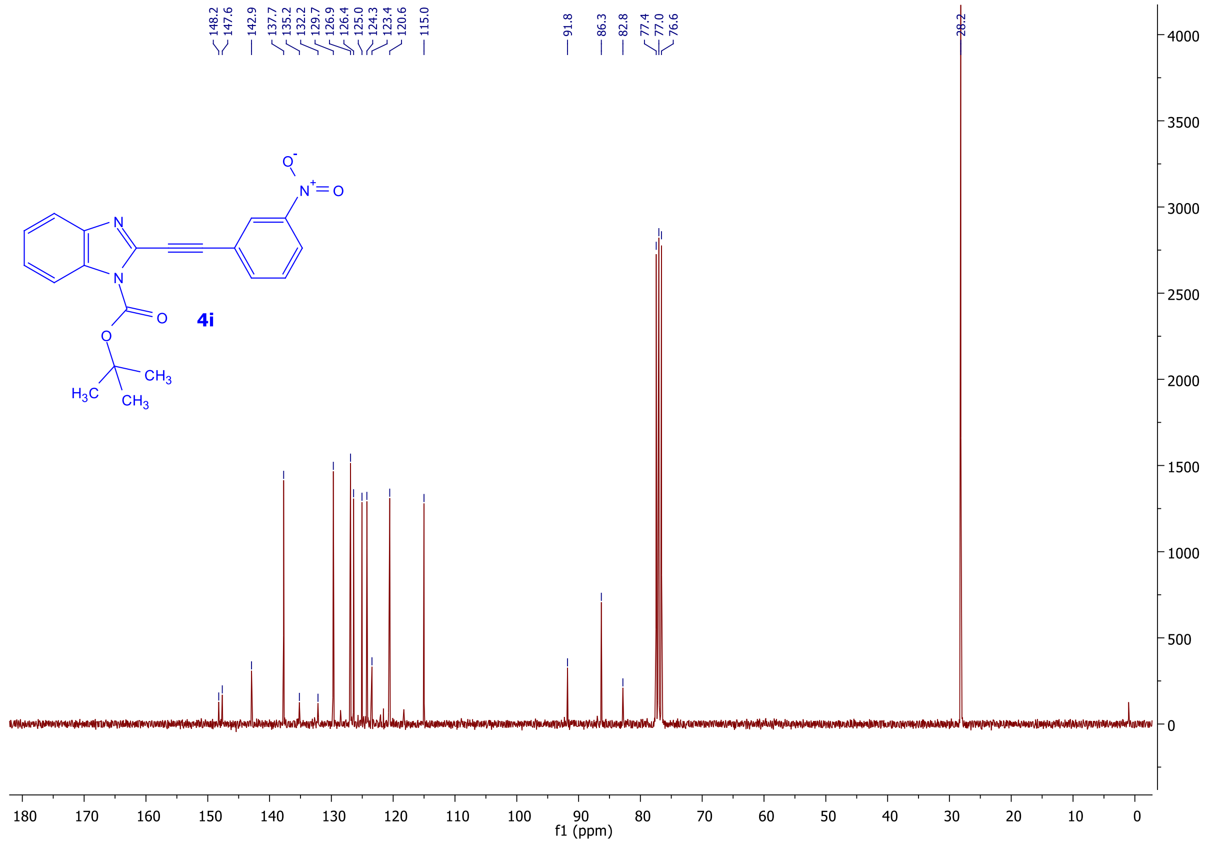

^{1}H NMR (300 MHz, $CDCl_3$) of **4j**

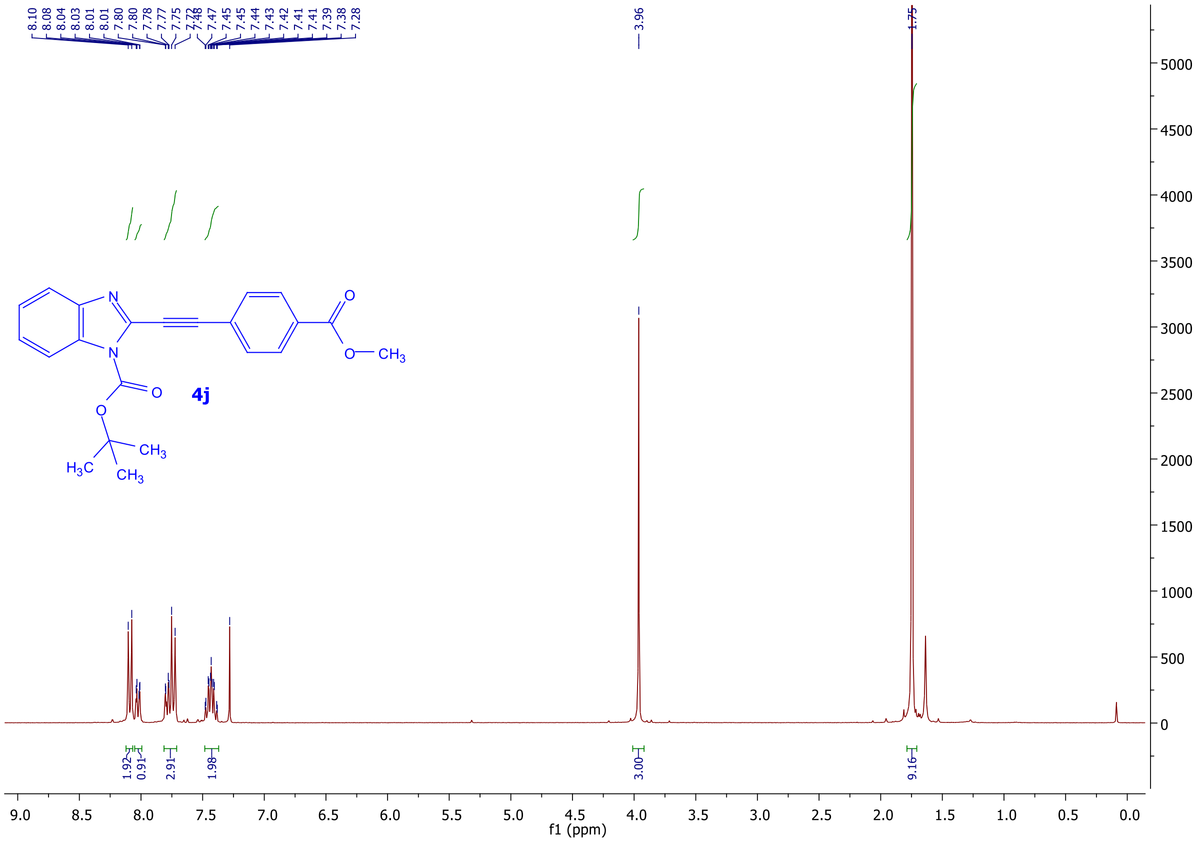

^{13}C NMR (75 MHz, $CDCl_3$) of **4j**

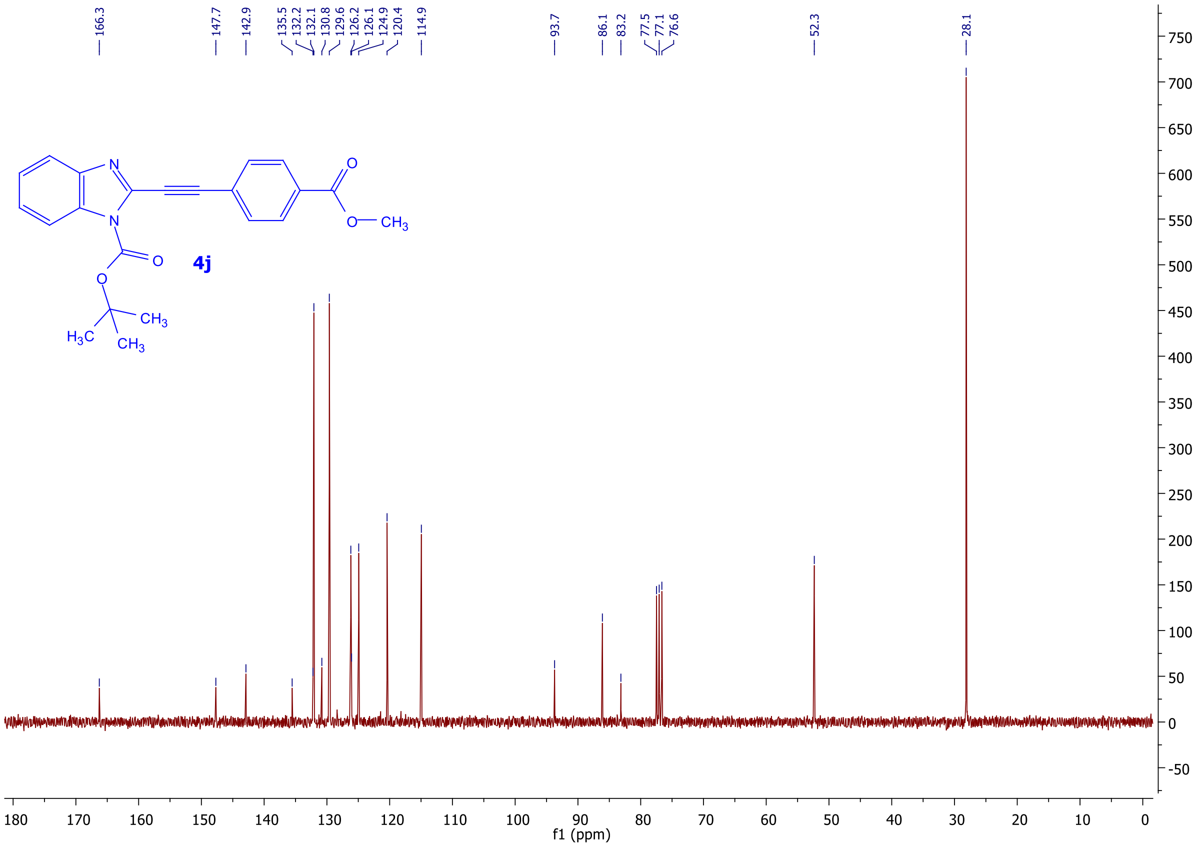

^{1}H NMR (300 MHz, $CDCl_3$) of **4k**

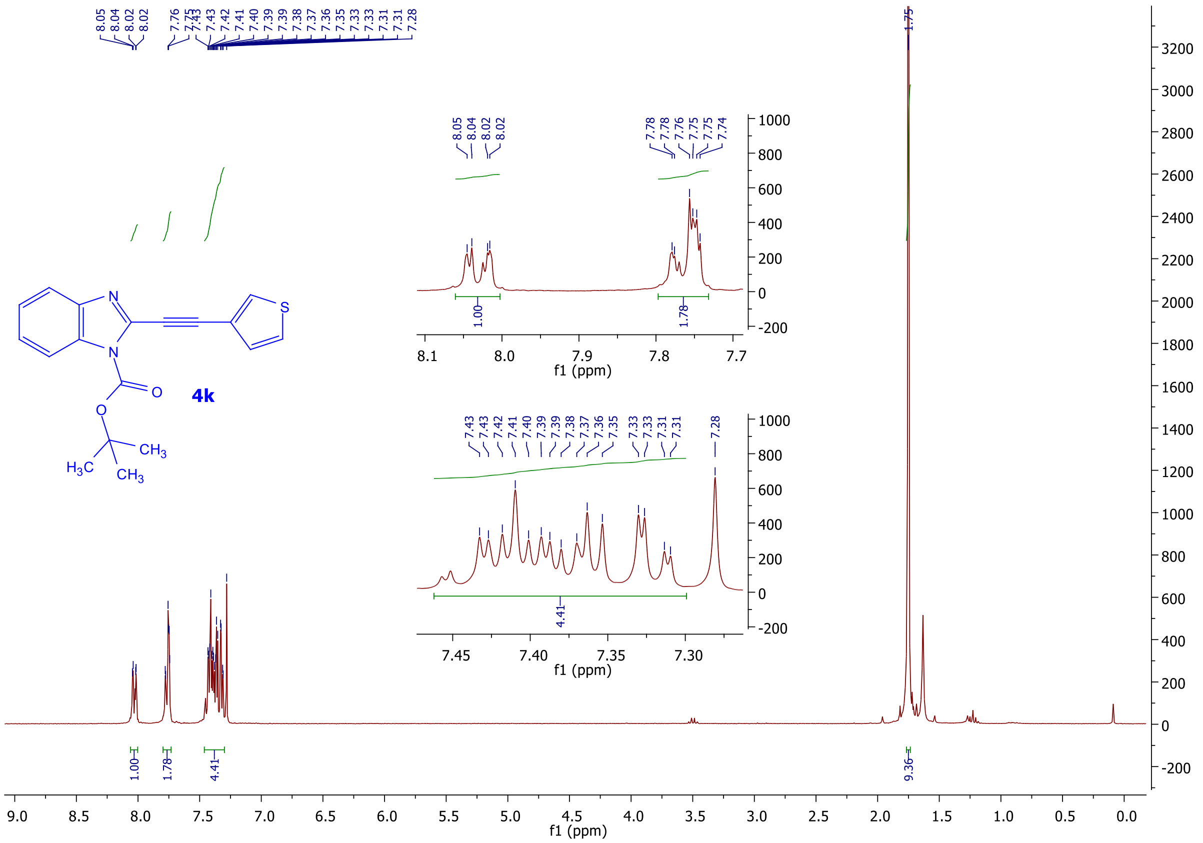

^{13}C NMR (75 MHz, $CDCl_3$) of **4k**

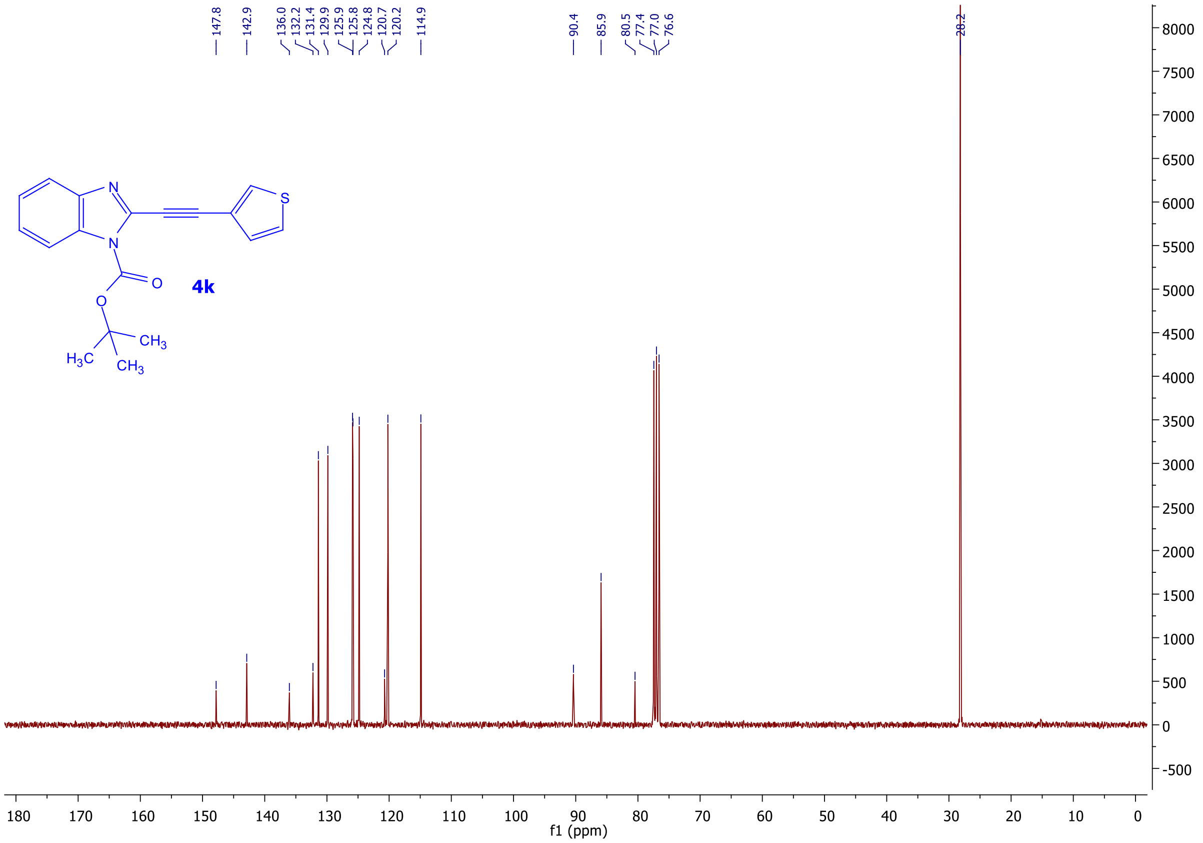

^{1}H NMR (300 MHz, $CDCl_3$) of **4l**

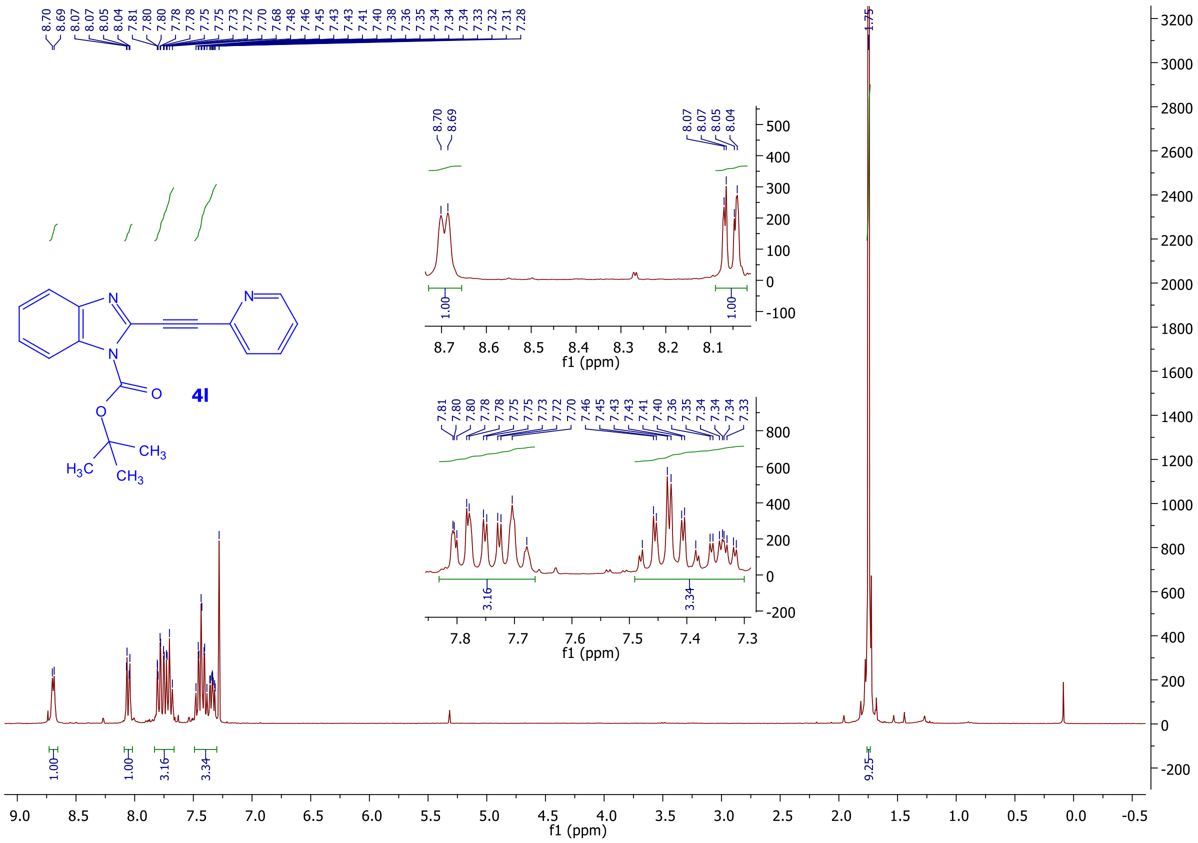

^{13}C NMR (75 MHz, $CDCl_3$) of **4l**

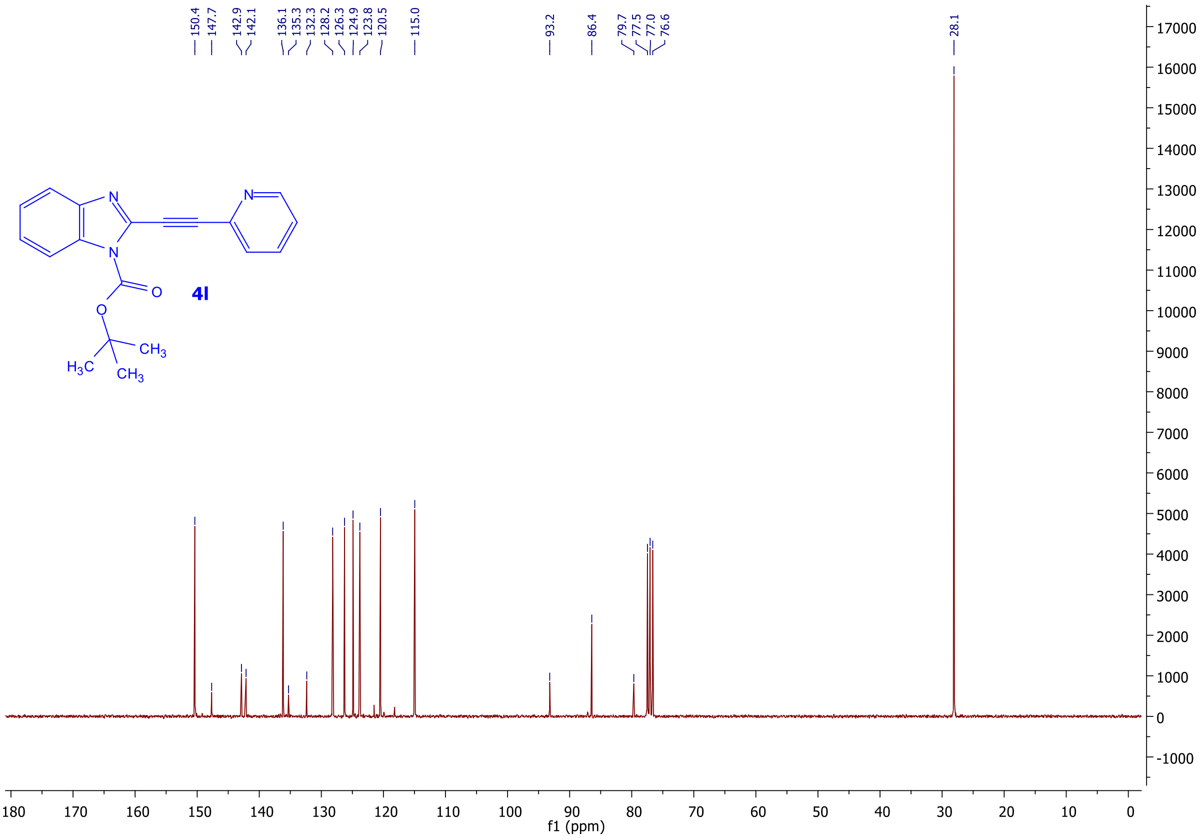

^1H NMR (300 MHz, $CDCl_3$) of **4m**

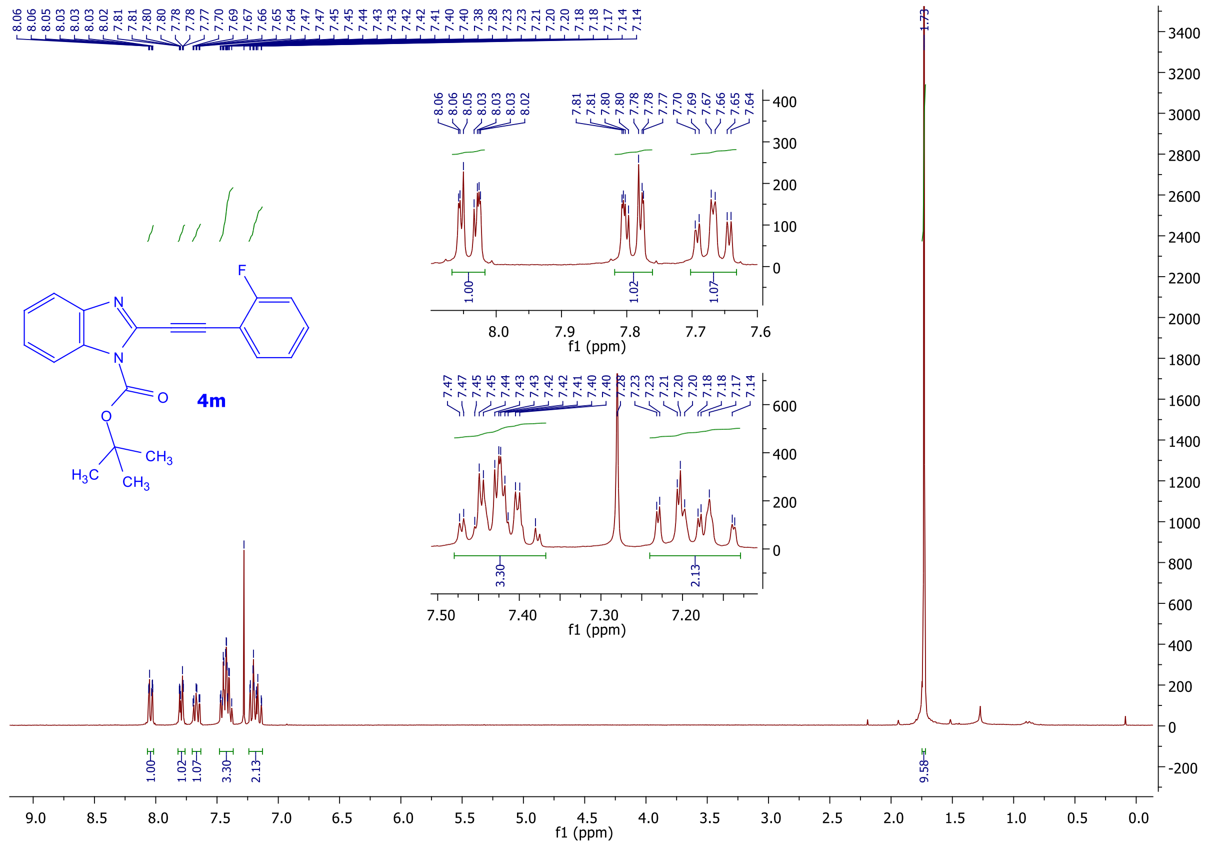

^{19}F NMR (282 MHz, $CDCl_3$) of **4m**

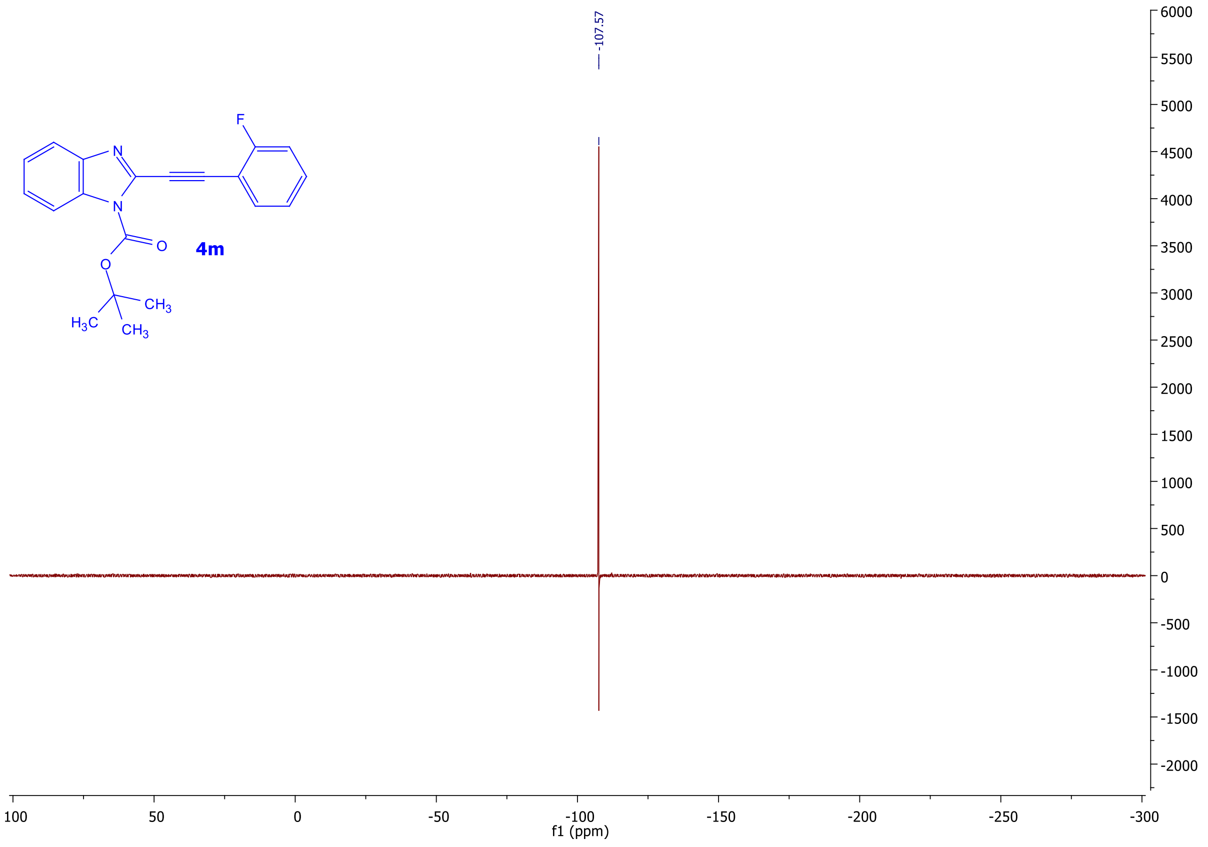

^{13}C NMR (75 MHz, $CDCl_3$) of **4m**

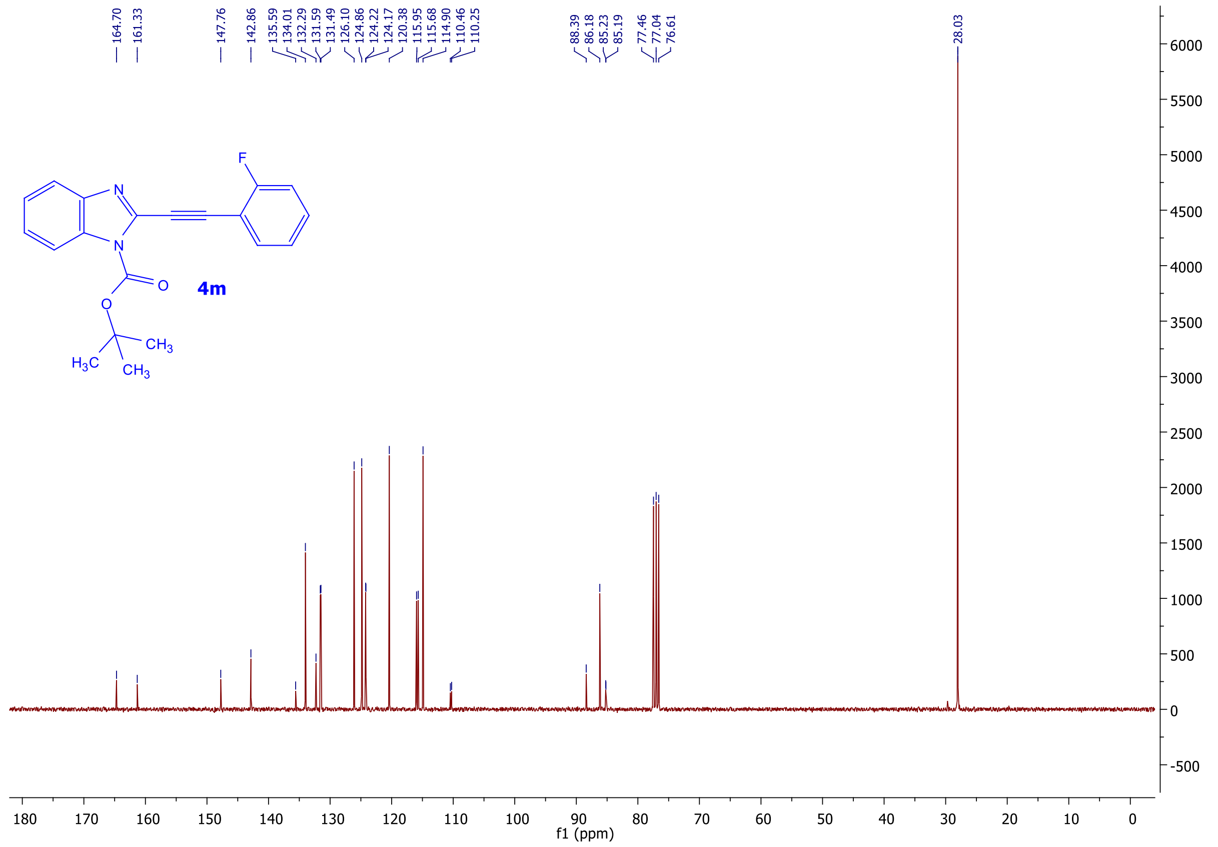

^{1}H NMR (300 MHz, $CDCl_3$) of **4n**

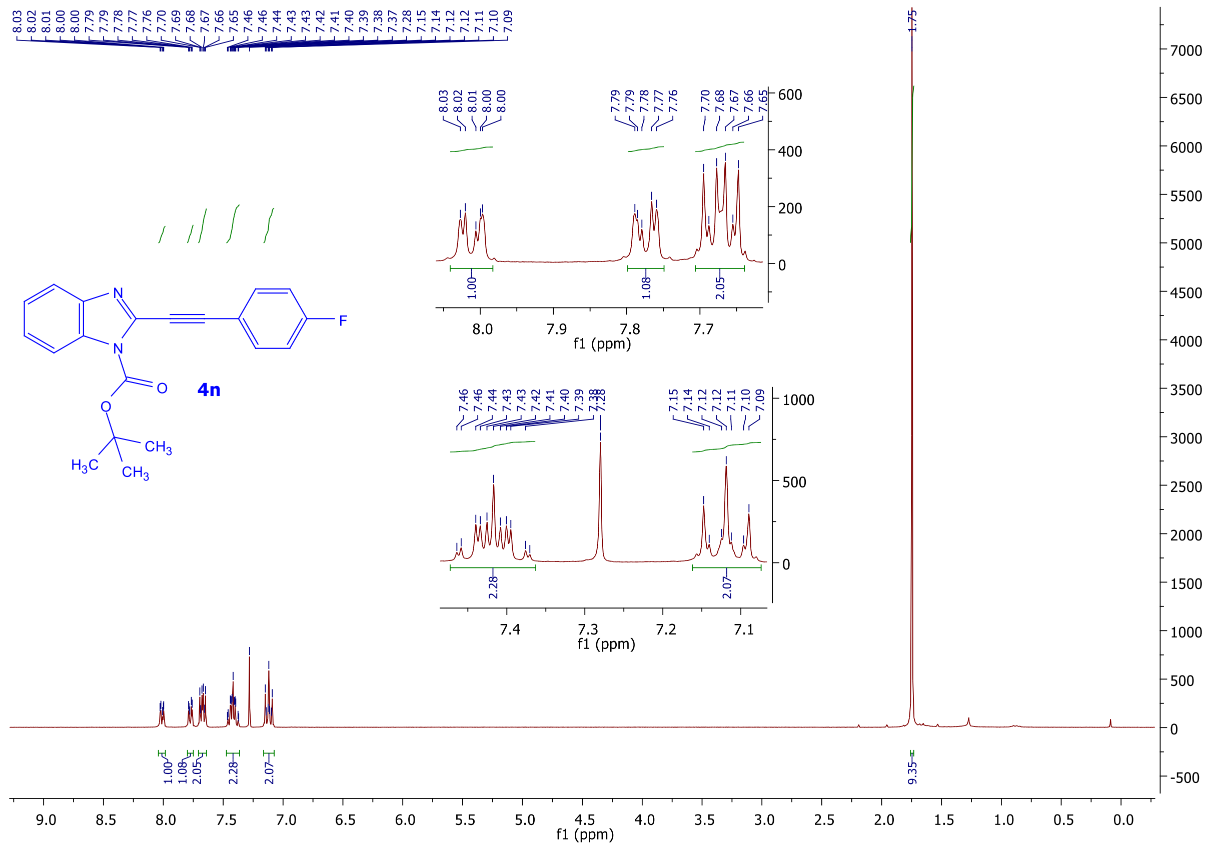

^{19}F NMR (282 MHz, $CDCl_3$) of **4n**

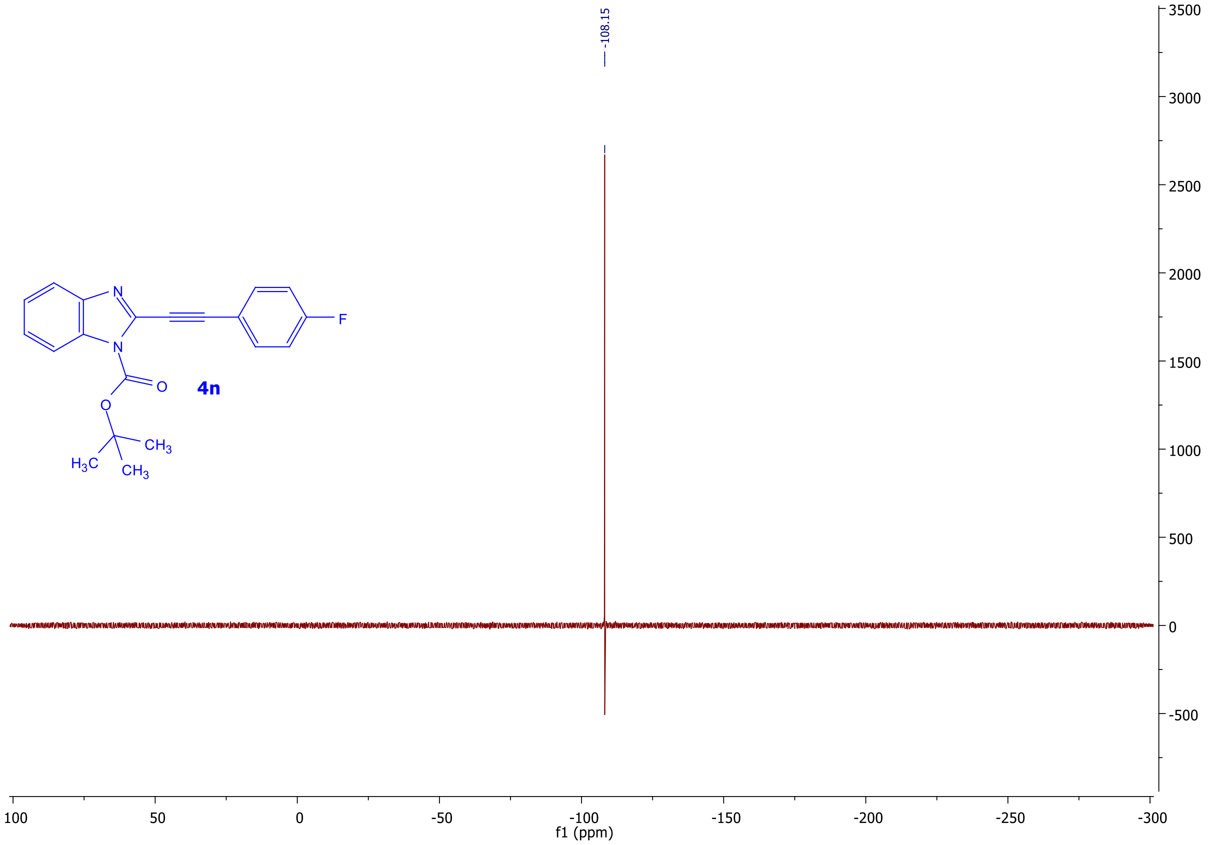

^{13}C NMR (75 MHz, $CDCl_3$) of **4n**

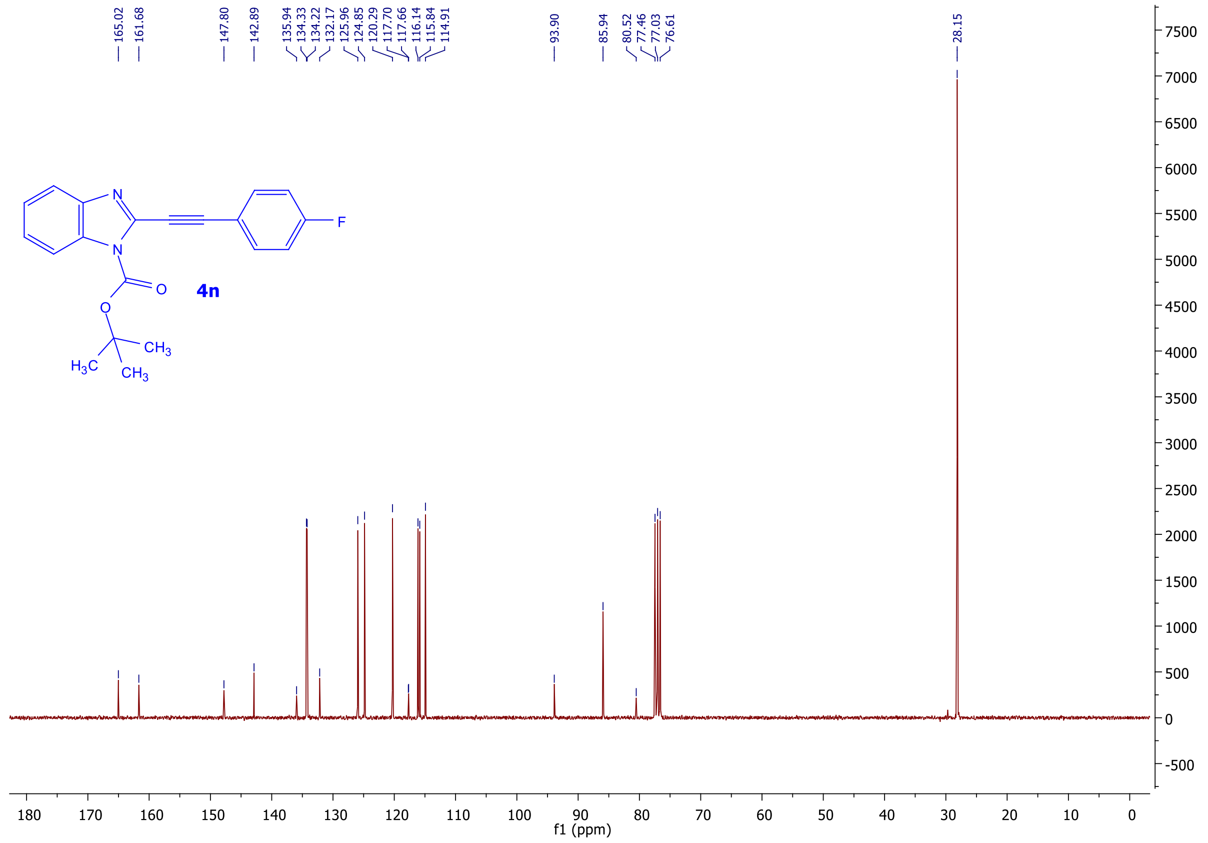

^{1}H NMR (300 MHz, $CDCl_3$) of **4o**

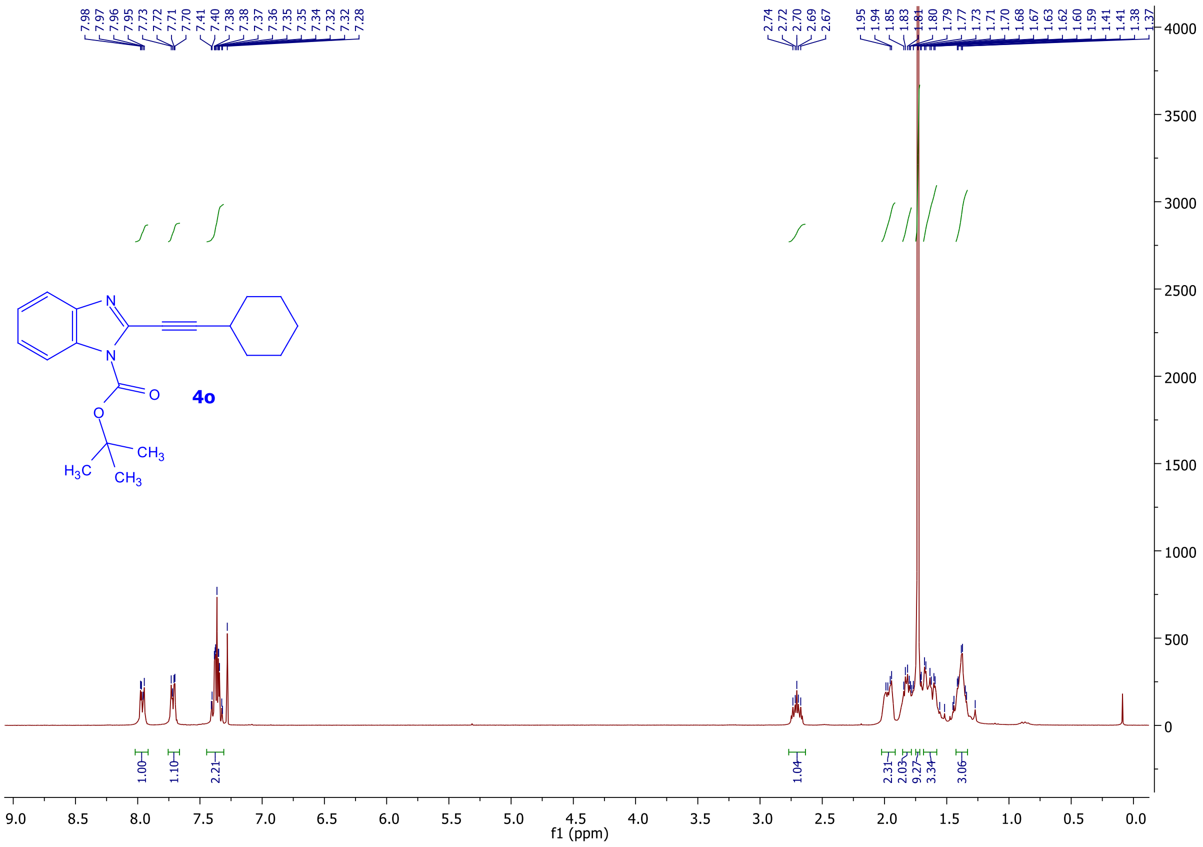

^{13}C NMR (75 MHz, $CDCl_3$) of **4o**

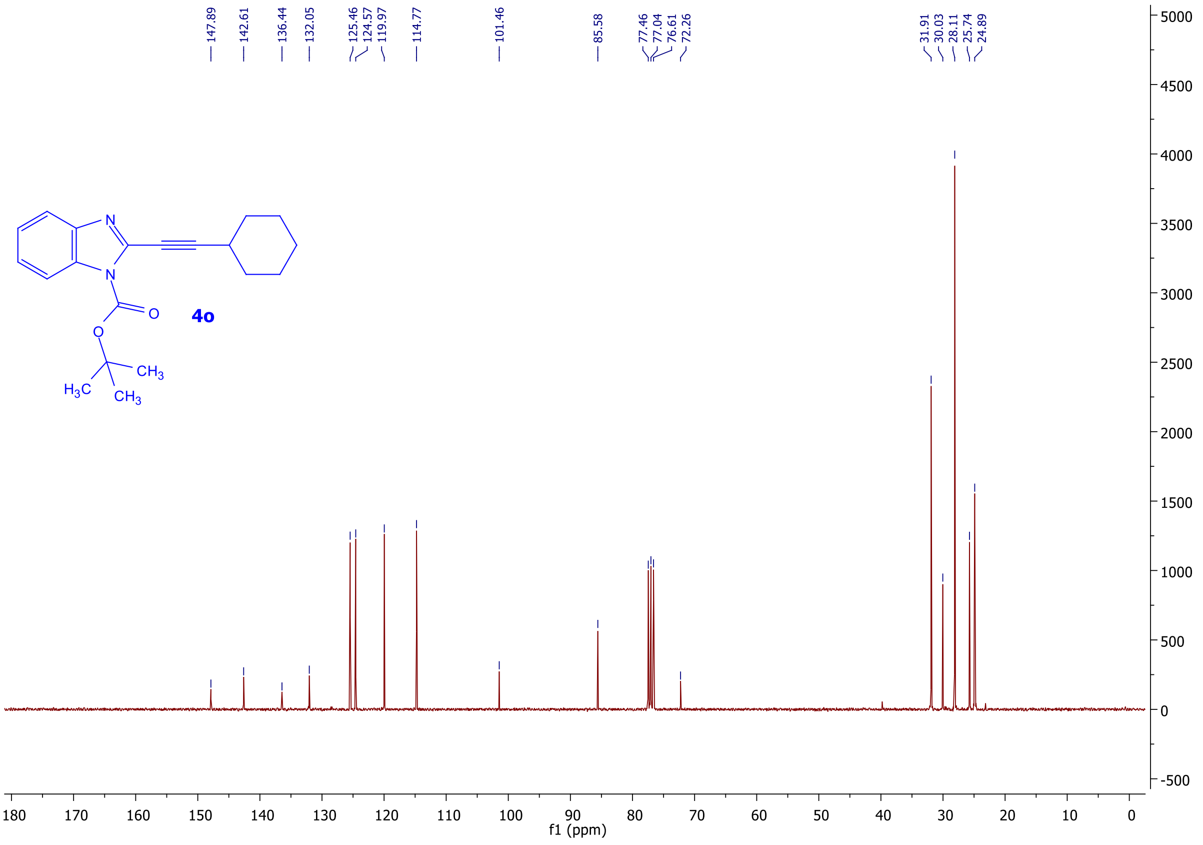

^{1}H NMR (300 MHz, $CDCl_3$) of **5a**

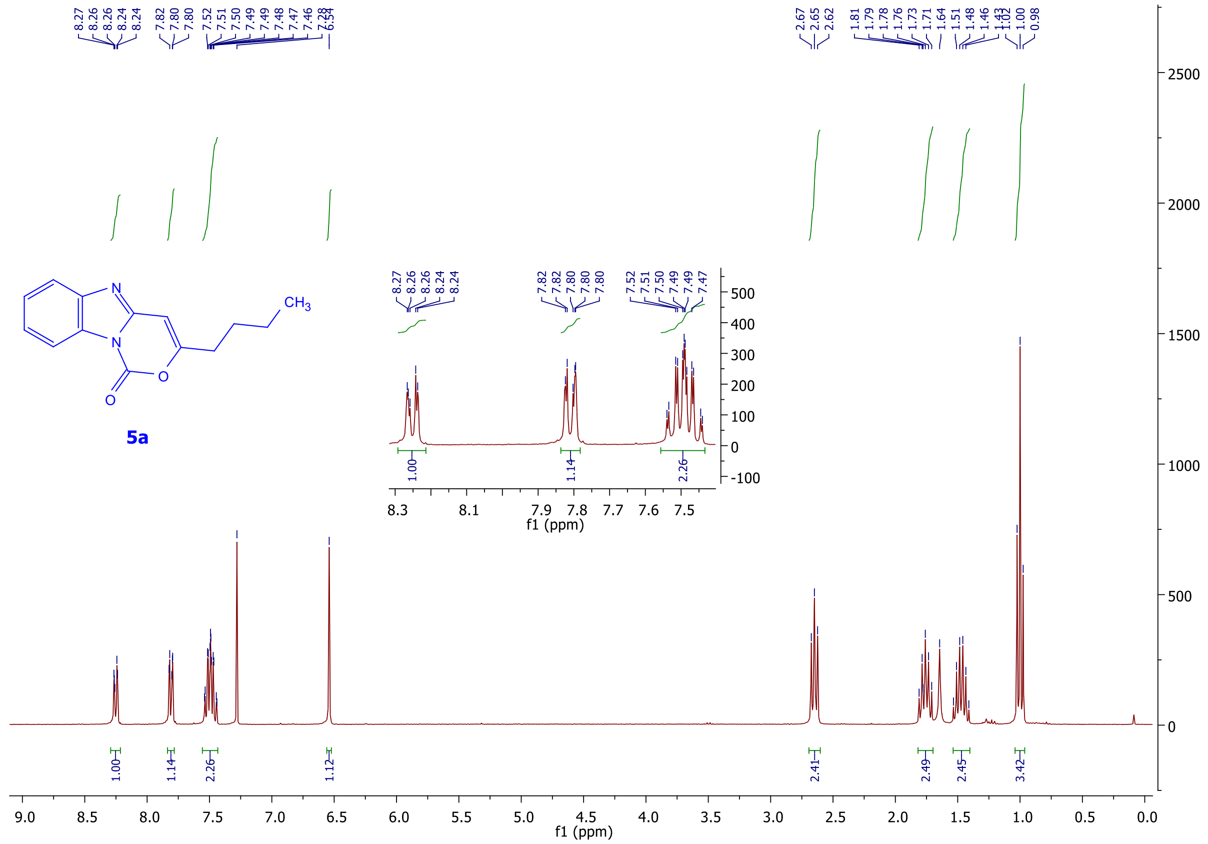

^{13}C NMR (75 MHz, $CDCl_3$) of **5a**

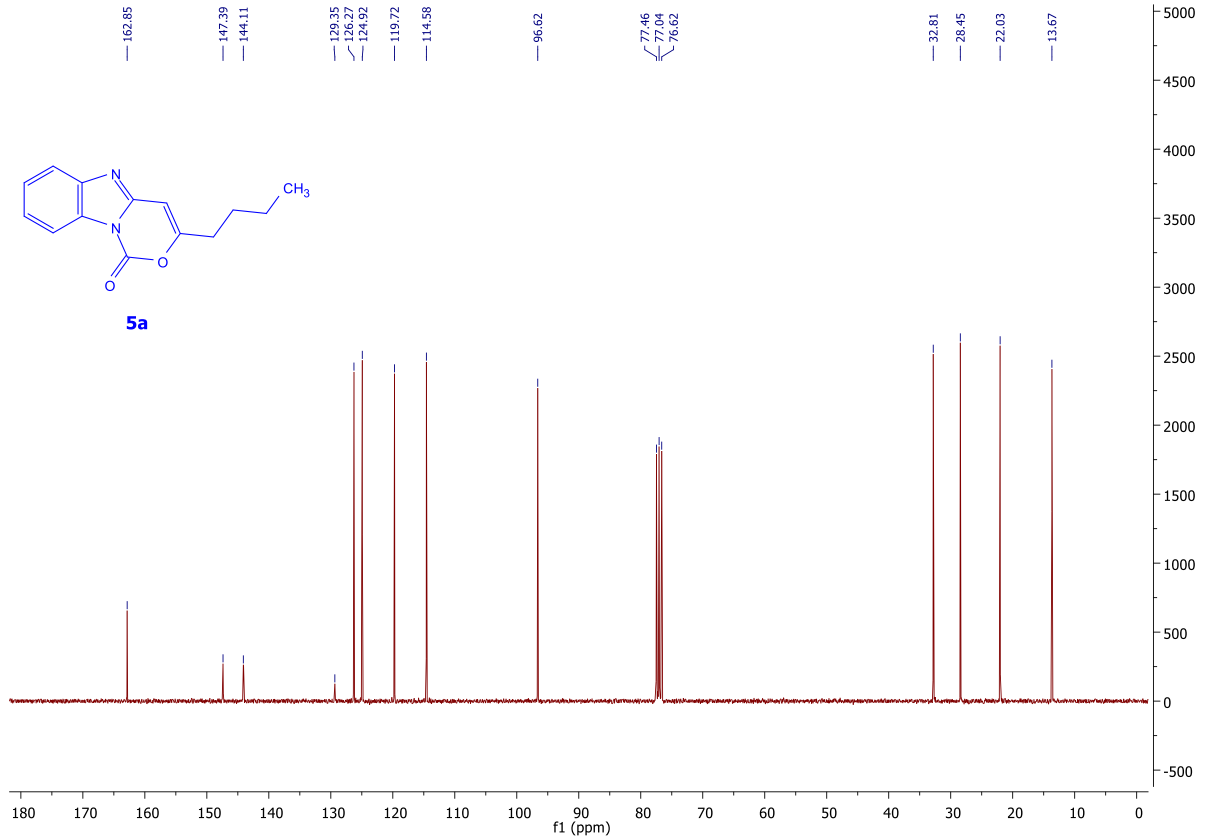

^{1}H NMR (300 MHz, $CDCl_3$) of **5b**

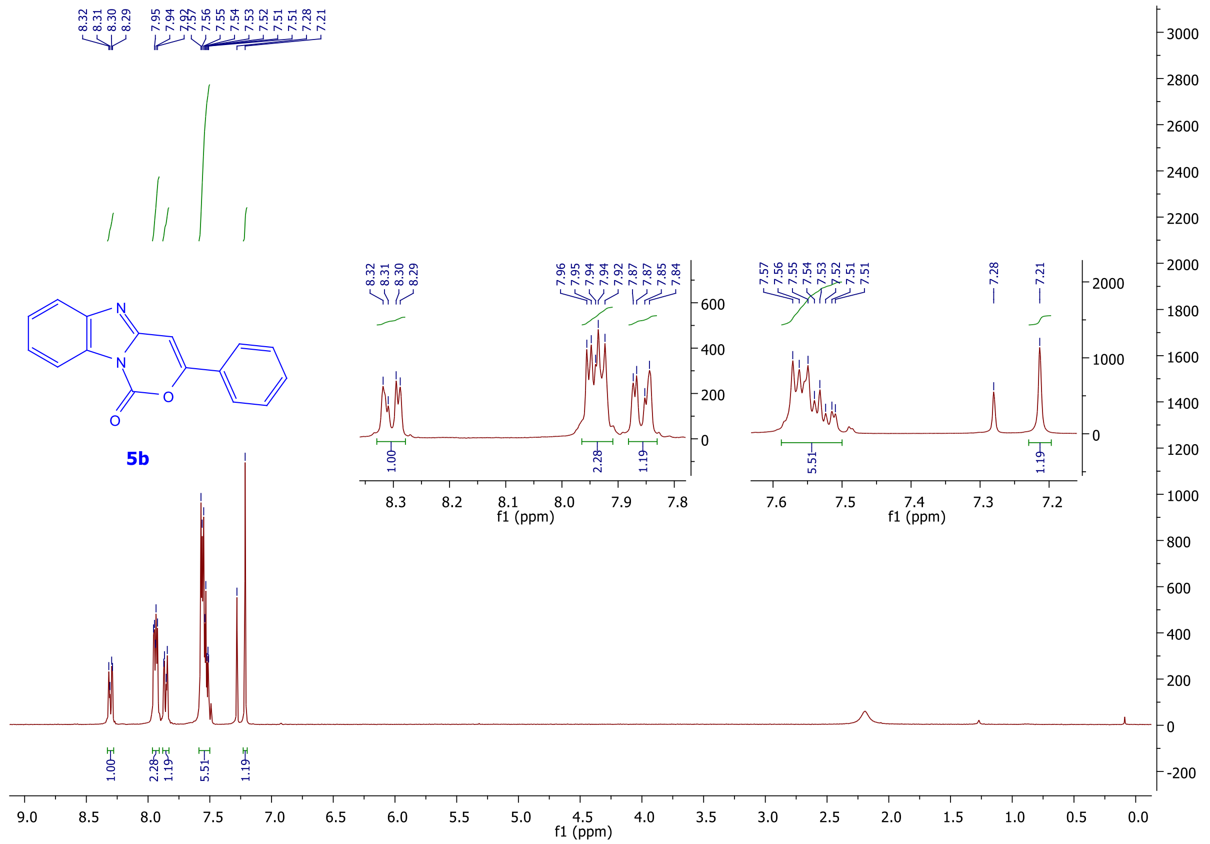

^{13}C NMR (75 MHz, $CDCl_3$) of **5b**

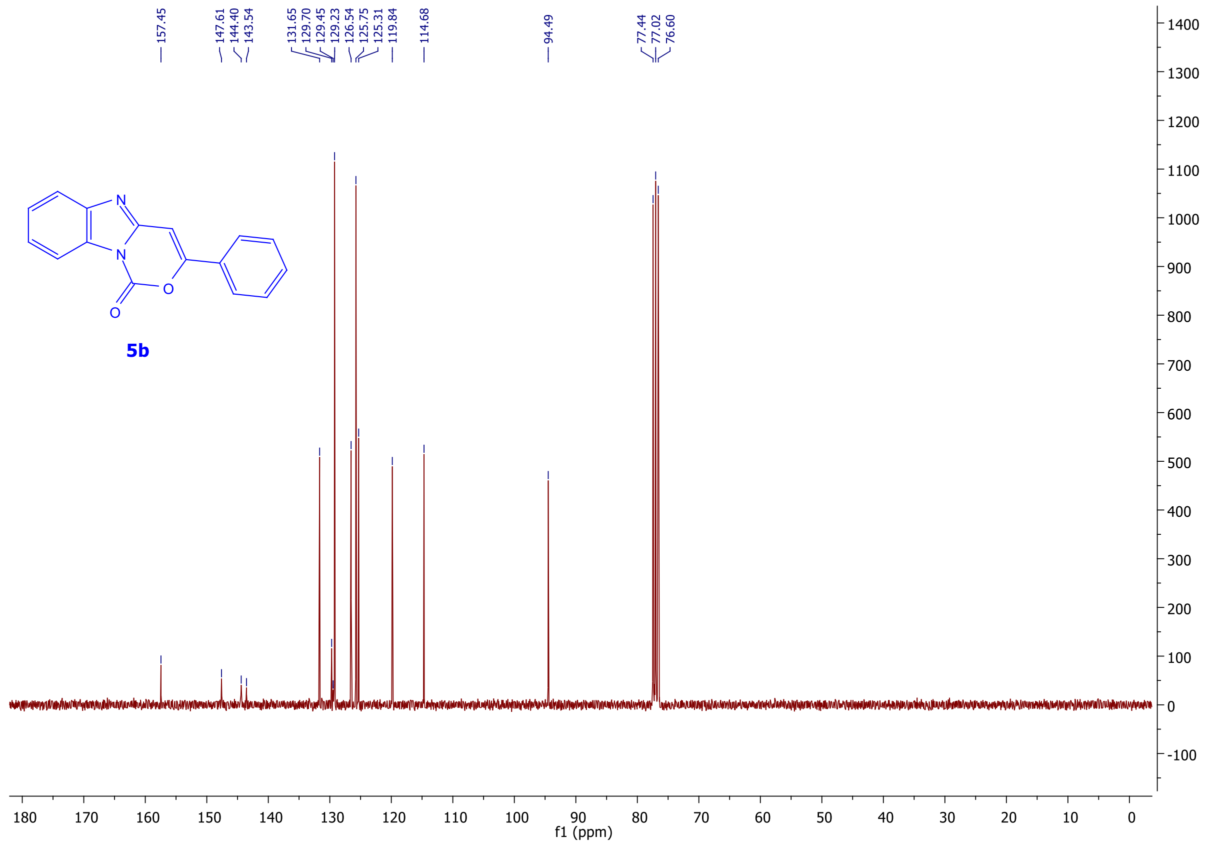

^{1}H NMR (300 MHz, $CDCl_3$) of **5c**

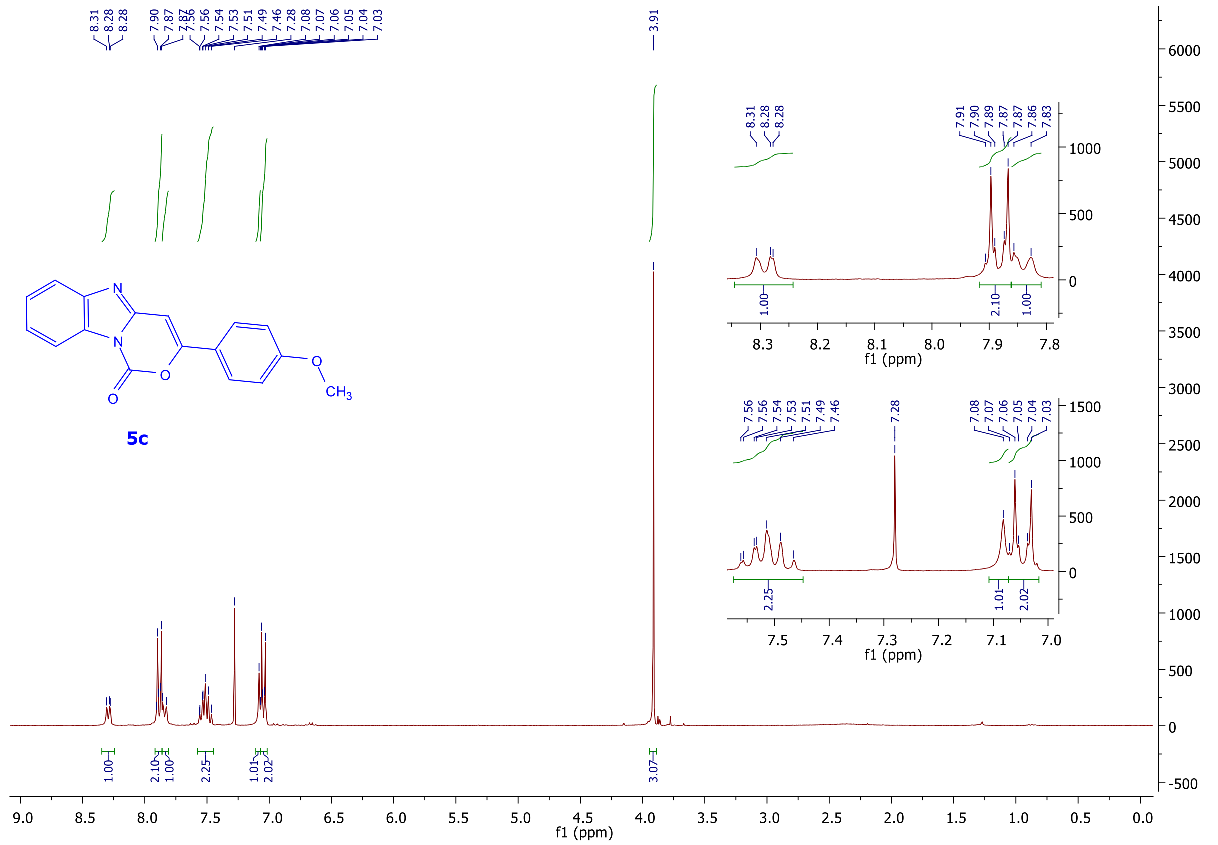

^{13}C NMR (75 MHz, $CDCl_3$) of **5c**

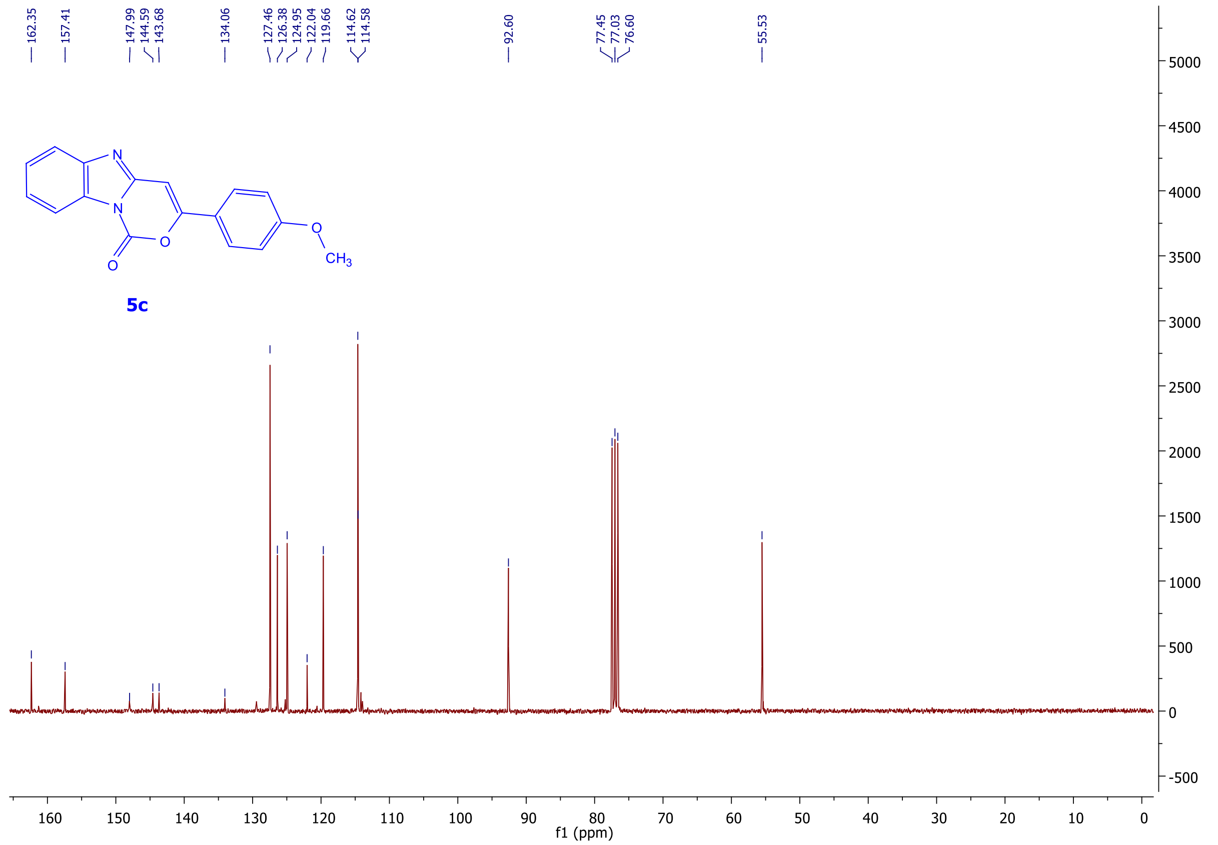

^{1}H NMR (300 MHz, $CDCl_3$) of **5d**

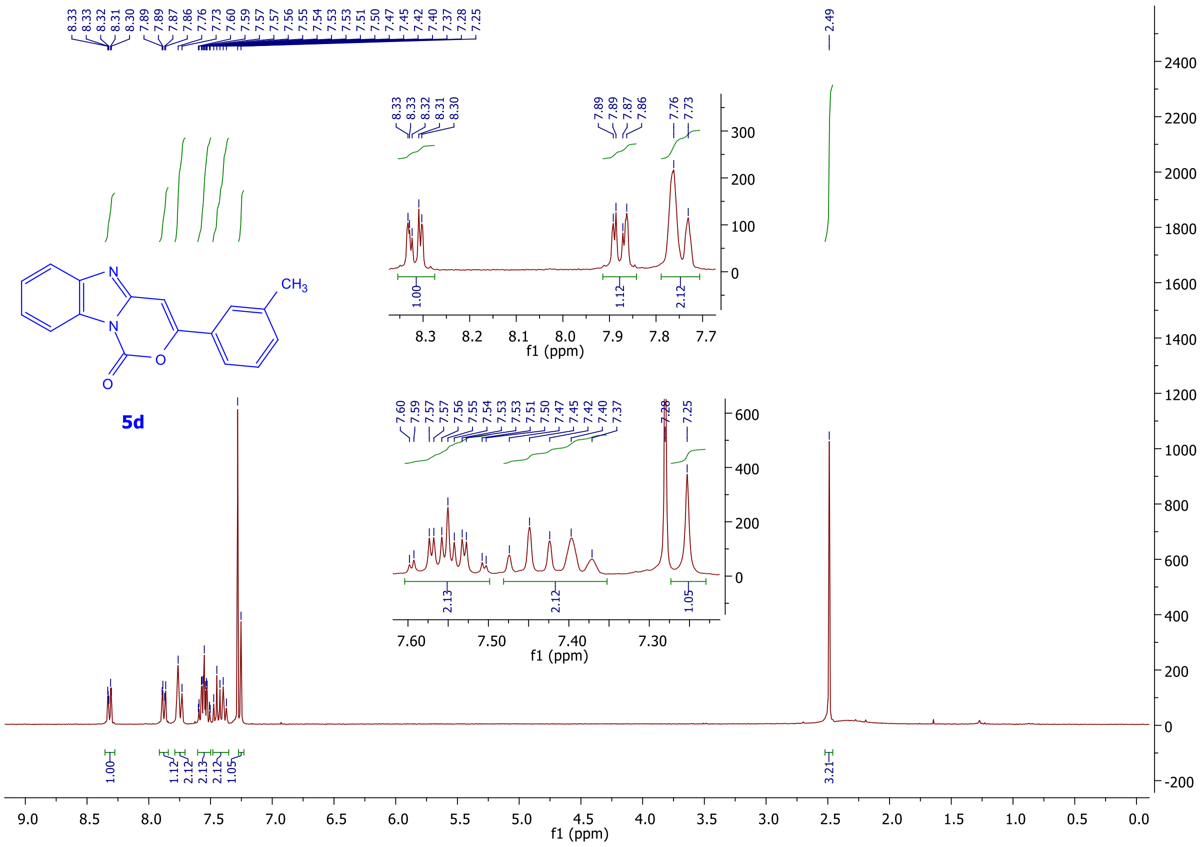

^{13}C NMR (75 MHz, $CDCl_3$) of **5d**

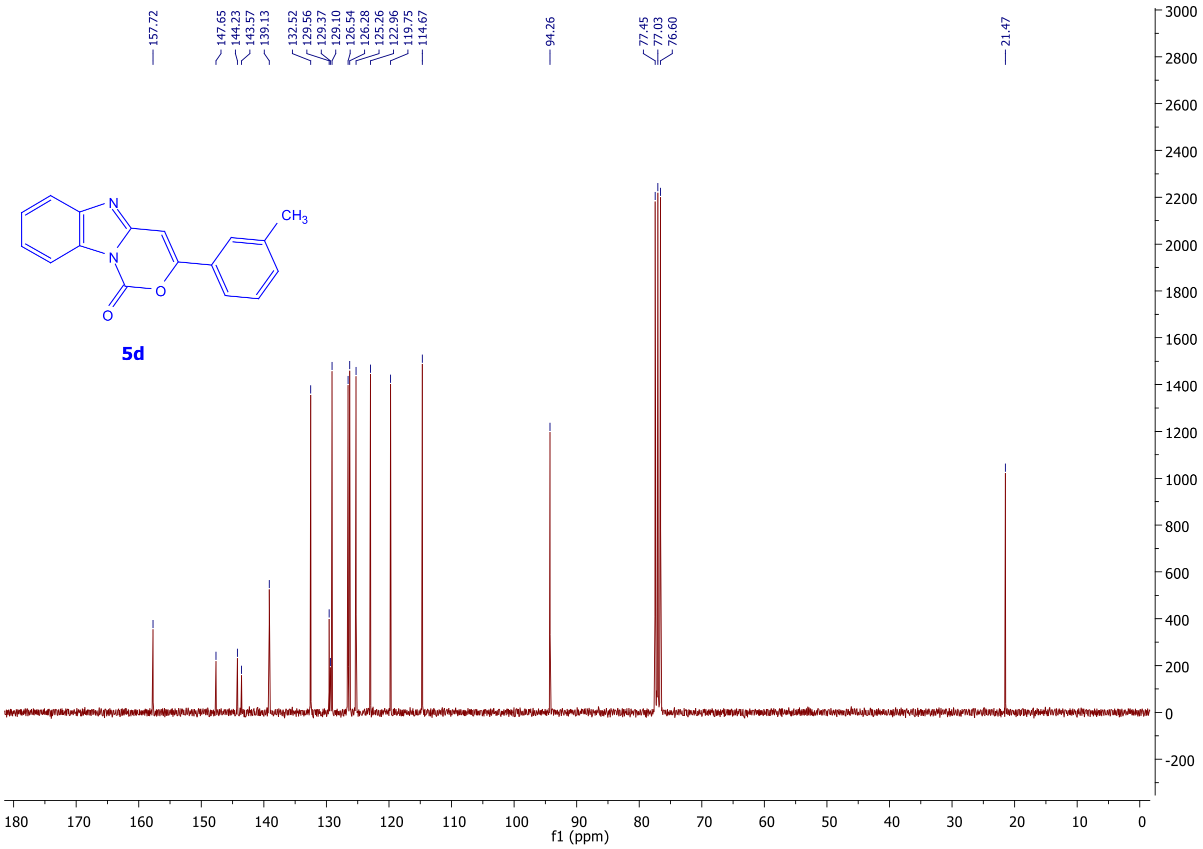

^{1}H NMR (300 MHz, $CDCl_3$) of **5e**

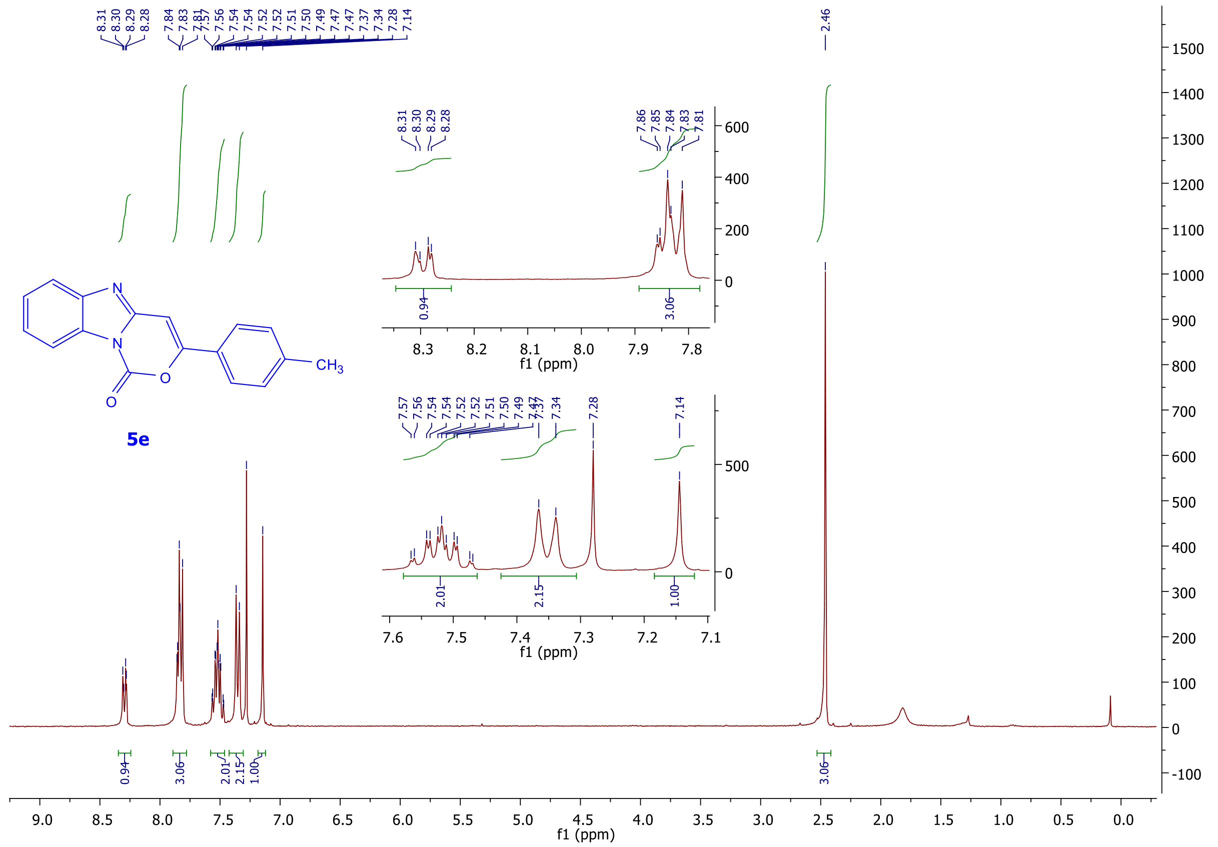

^{13}C NMR (75 MHz, $CDCl_3$) of **5e**

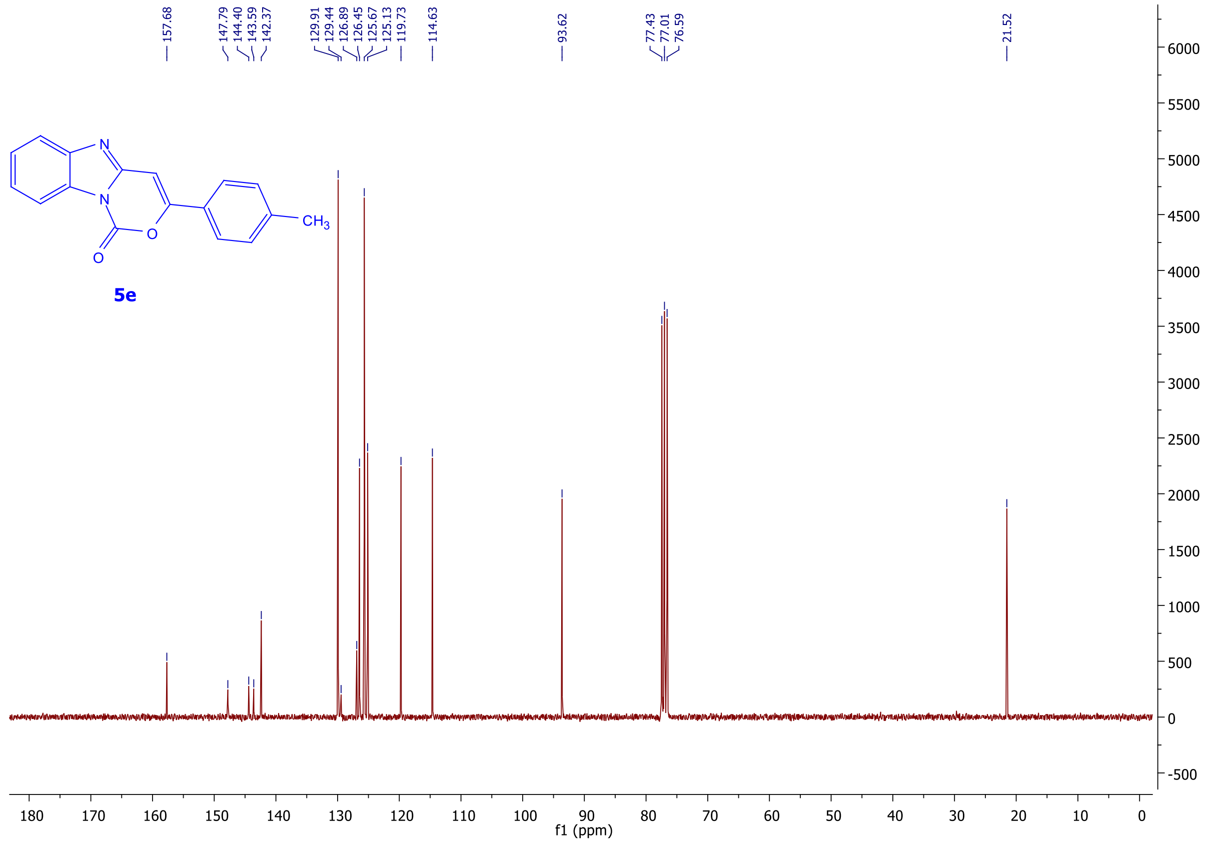

^{1}H NMR (300 MHz, $CDCl_3$) of **5f**

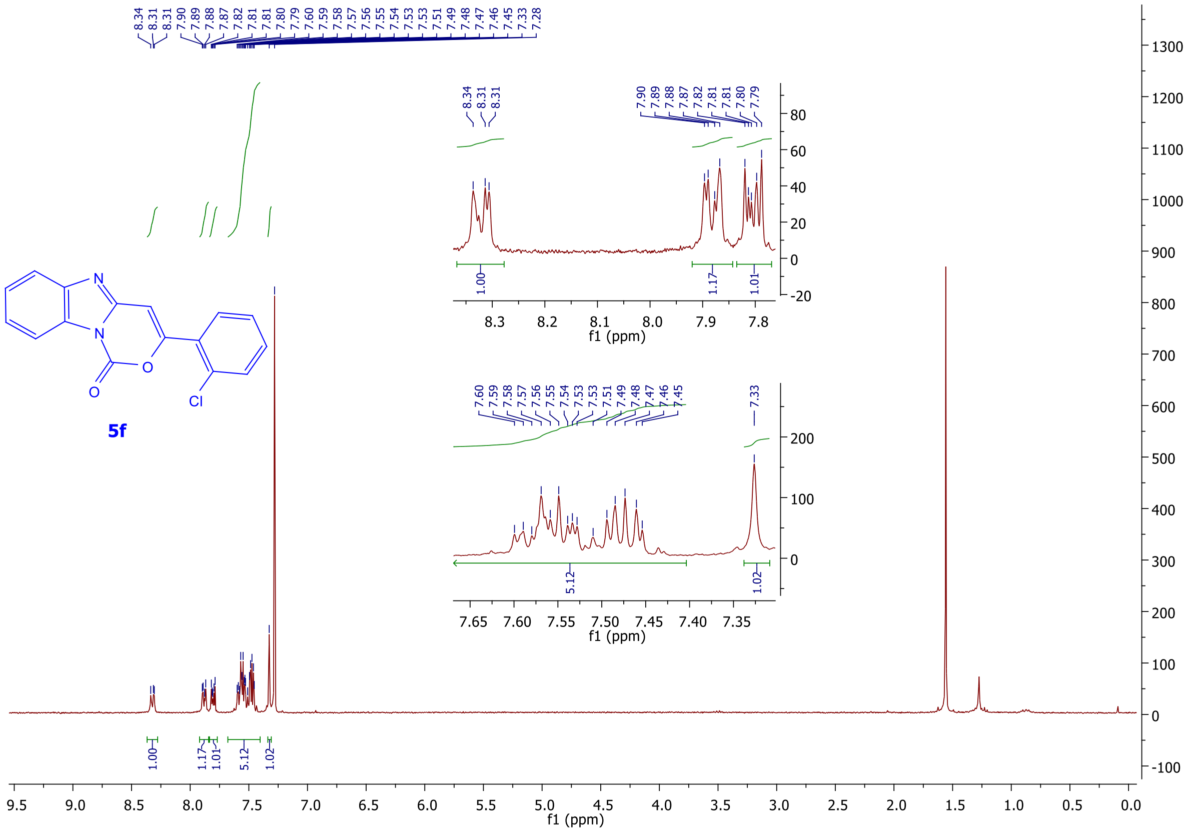

^{13}C NMR (75 MHz, $CDCl_3$) of **5f**

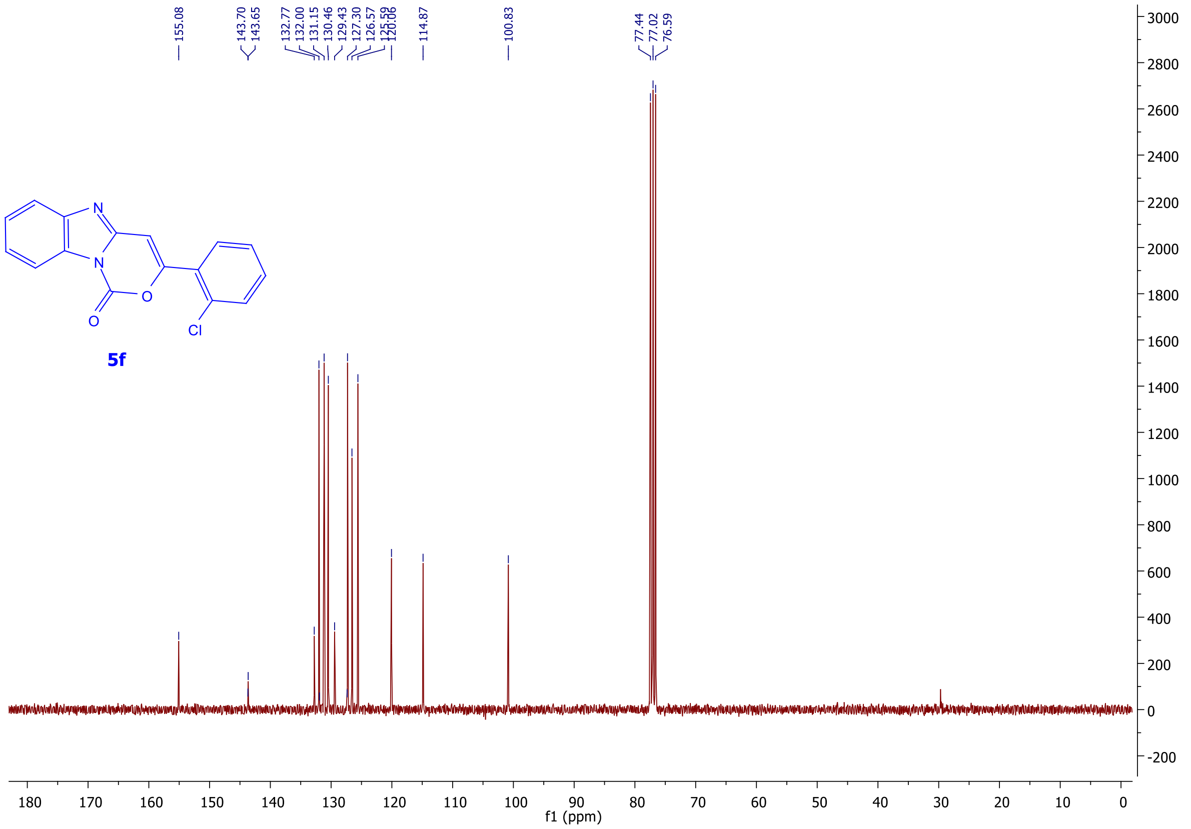

^{1}H NMR (300 MHz, DMSO-d_6) of **5g**

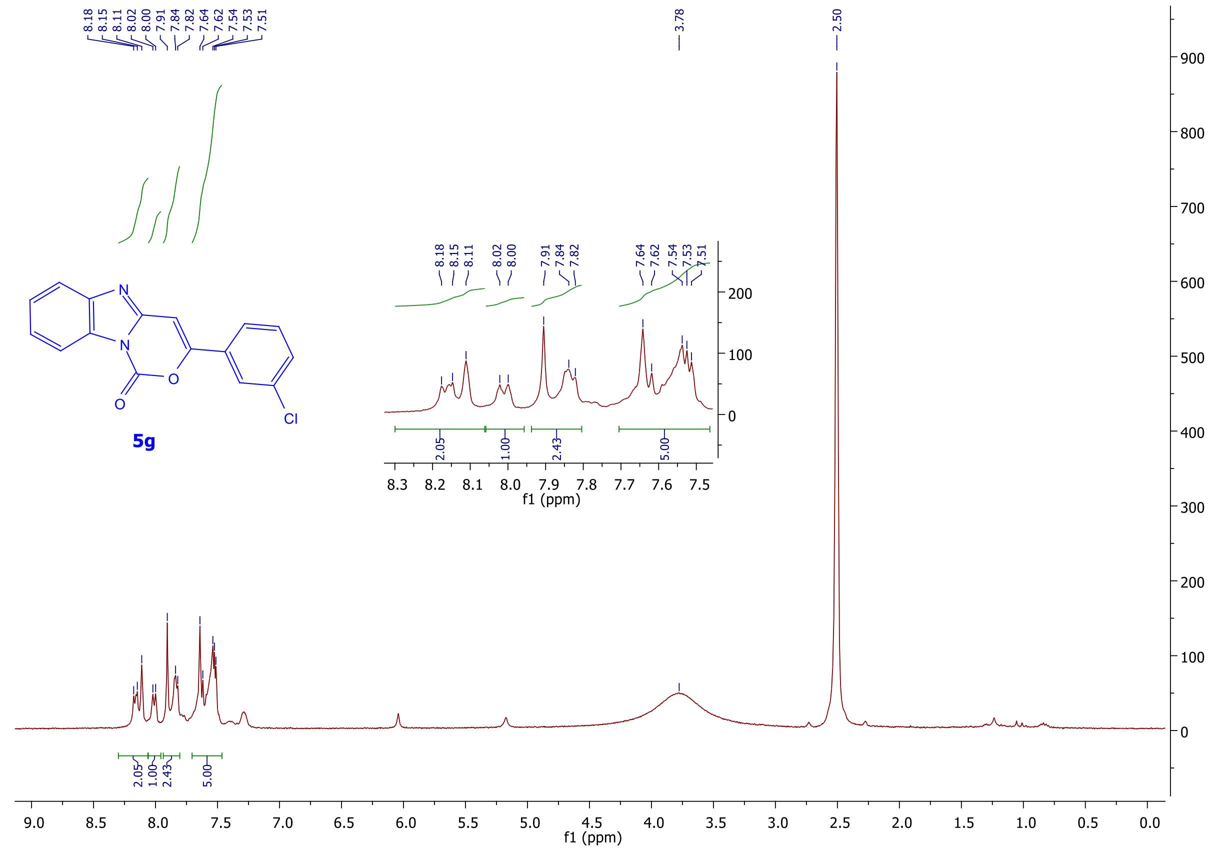

^{13}C NMR (75 MHz, $CDCl_3$) of **5g**

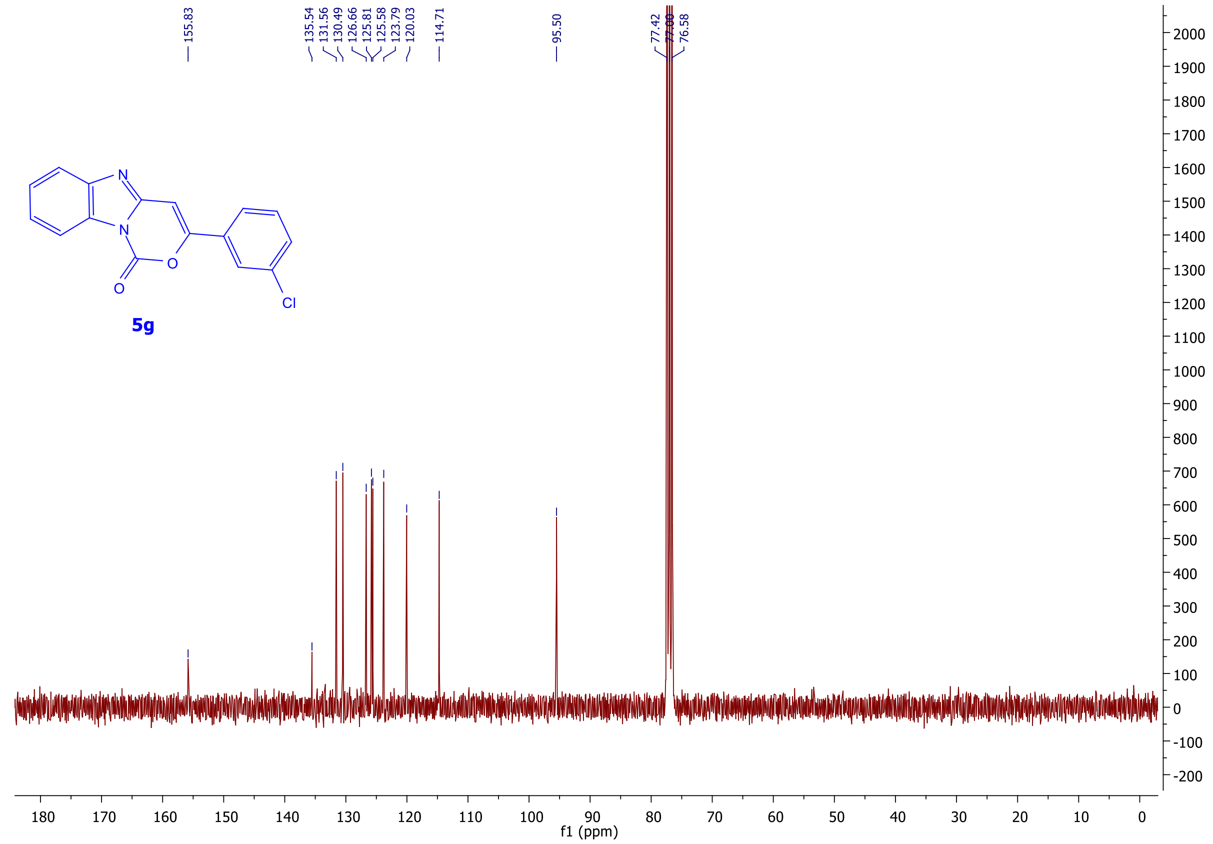

^{1}H NMR (300 MHz, $CDCl_3$) of **5h**

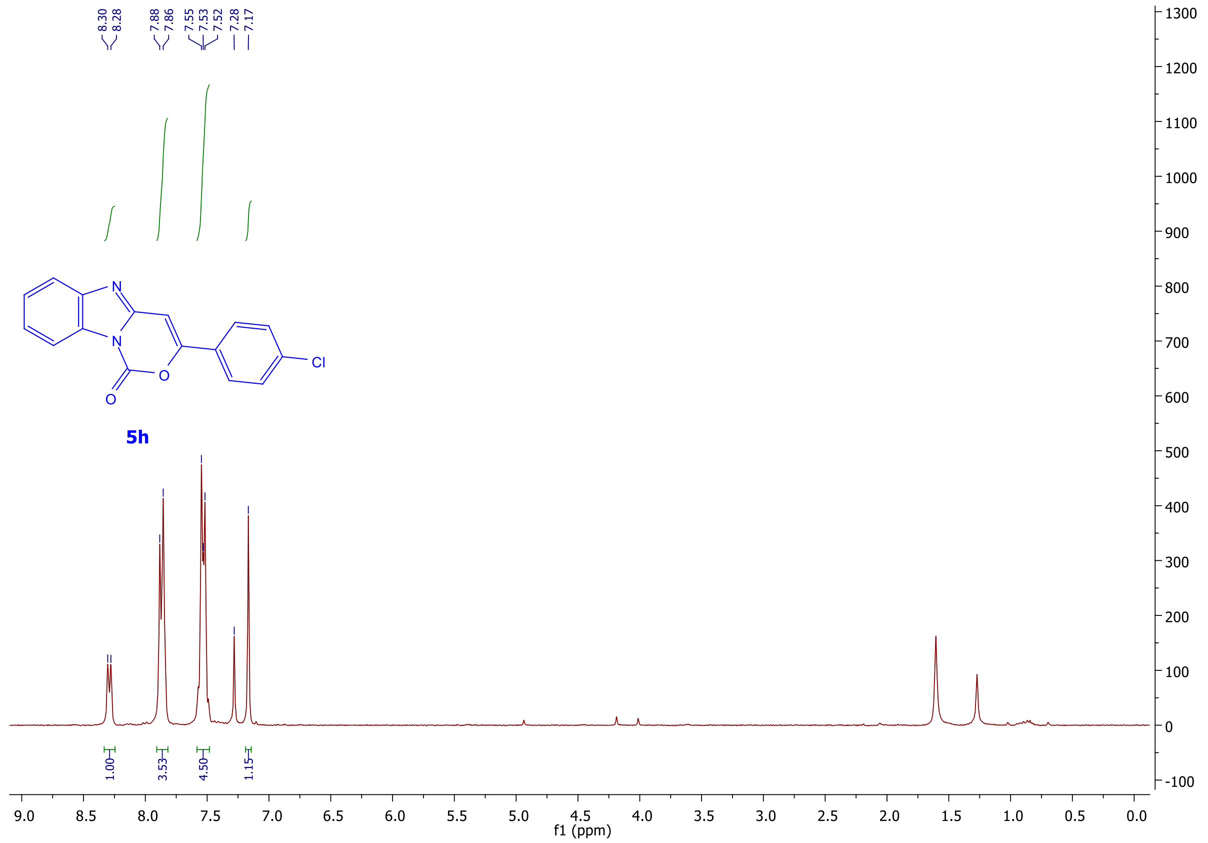

^{13}C NMR (75 MHz, $CDCl_3$) of **5h**

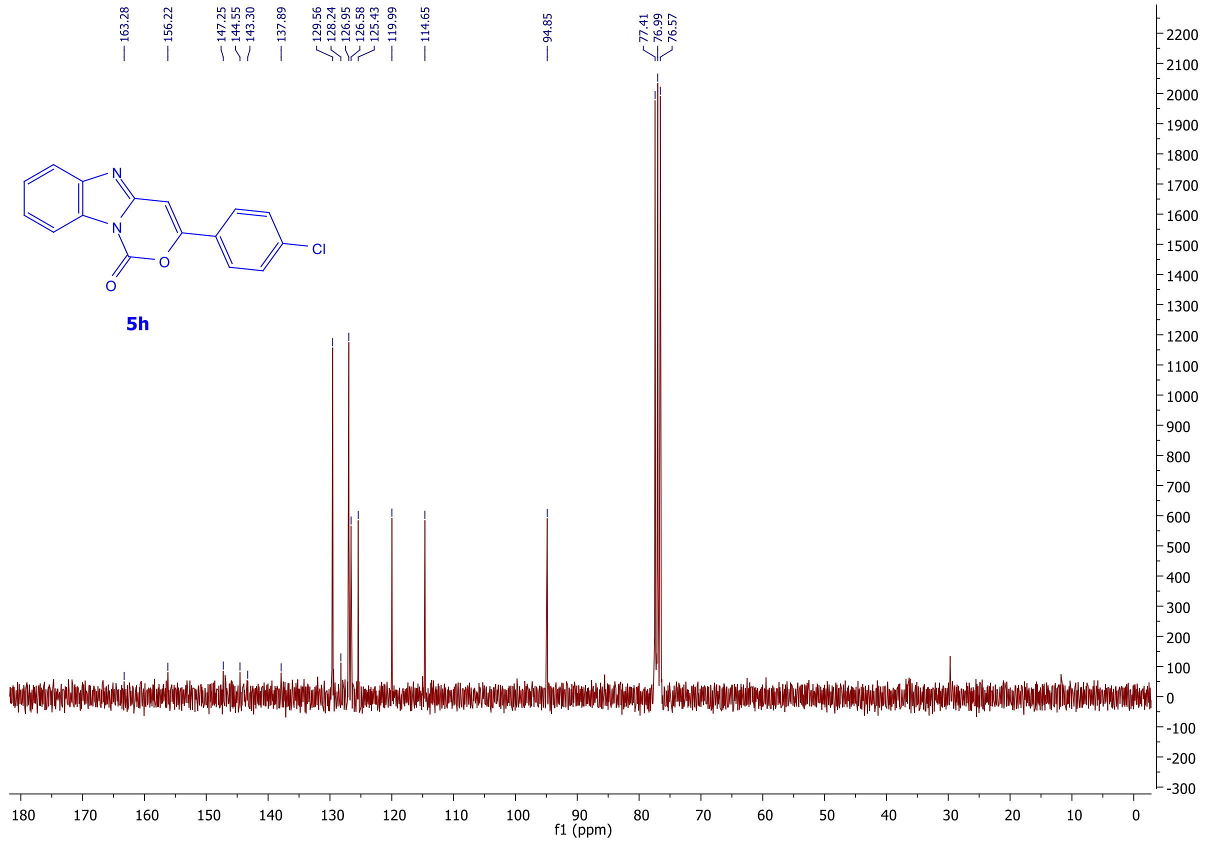

^{1}H NMR (300 MHz, $CDCl_3$) of **5i**

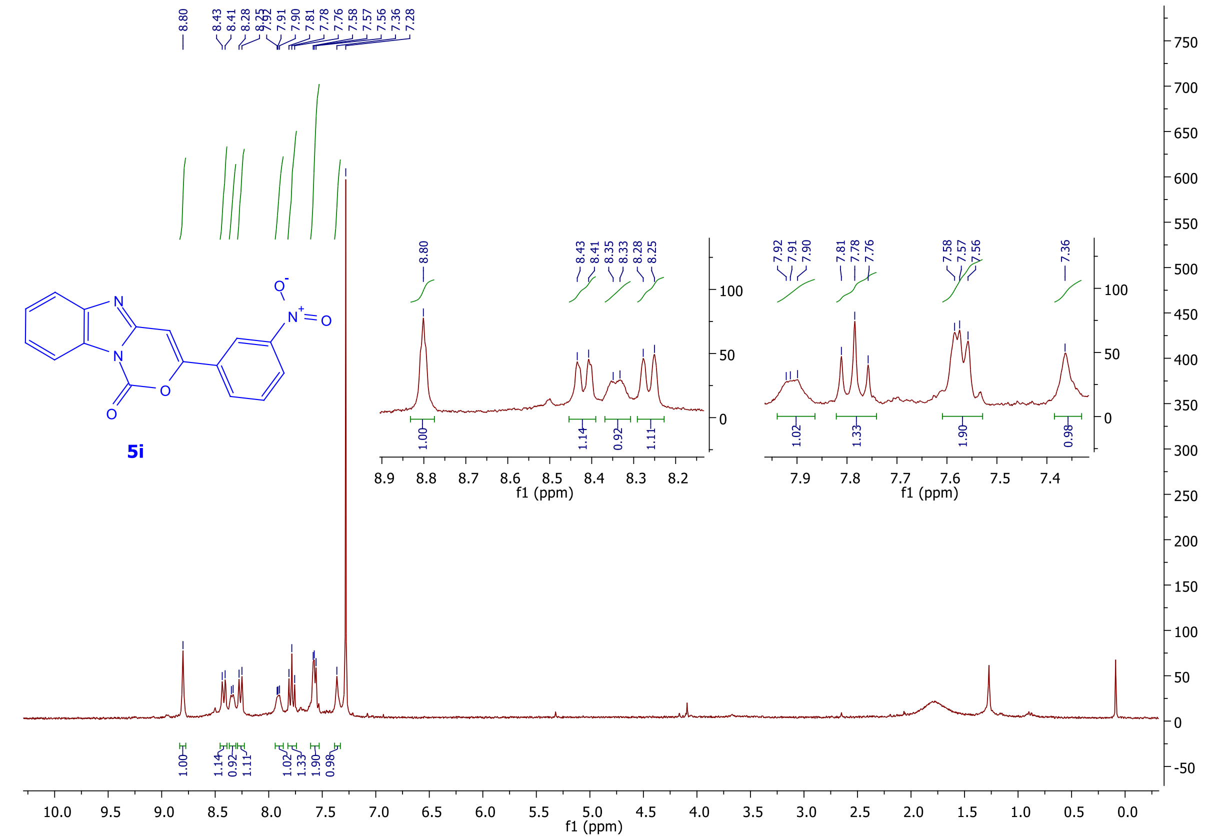

^{13}C NMR (75 MHz, $CDCl_3$/TFA-d_1) of **5i**

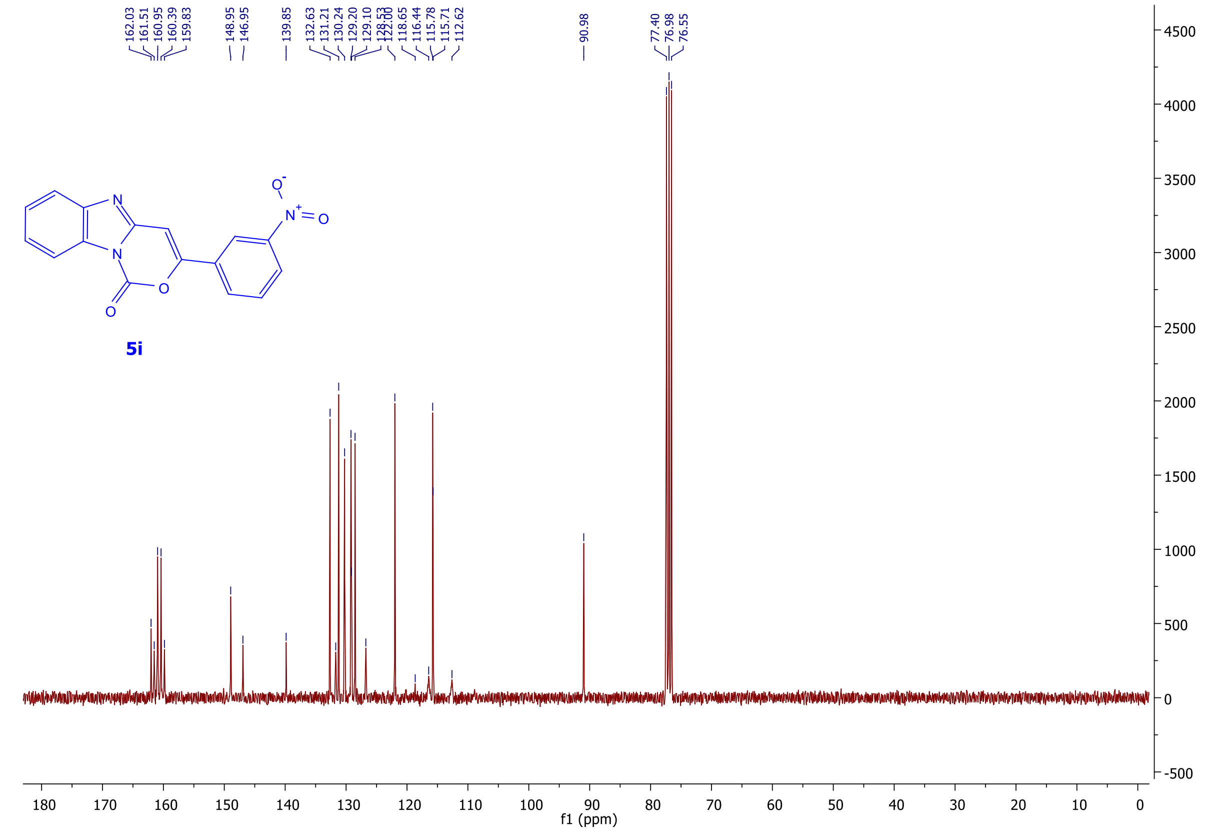

^{1}H NMR (300 MHz, $CDCl_3$) of **5j**

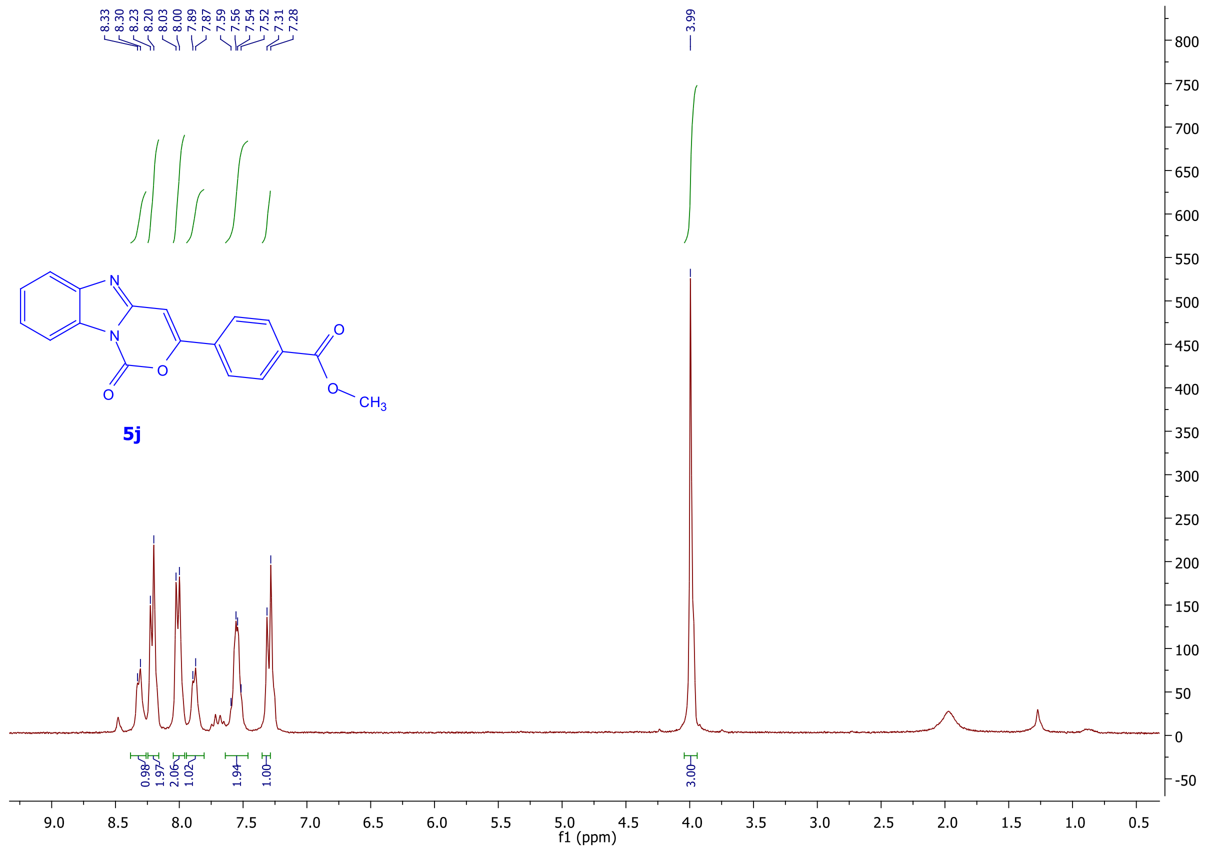

^{13}C NMR (75 MHz, $CDCl_3$/TFA-d_1) of **5j**

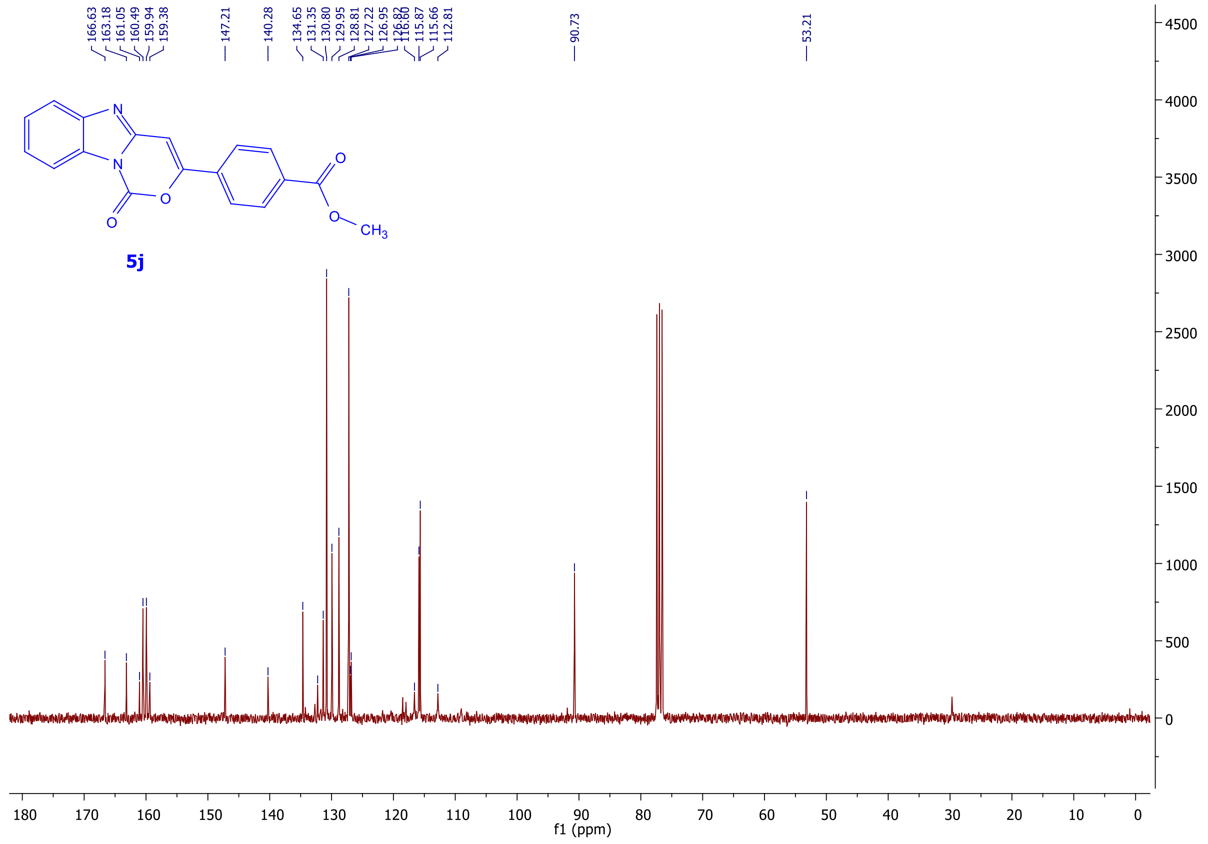

^{1}H NMR (300 MHz, $CDCl_3$) of **5k**

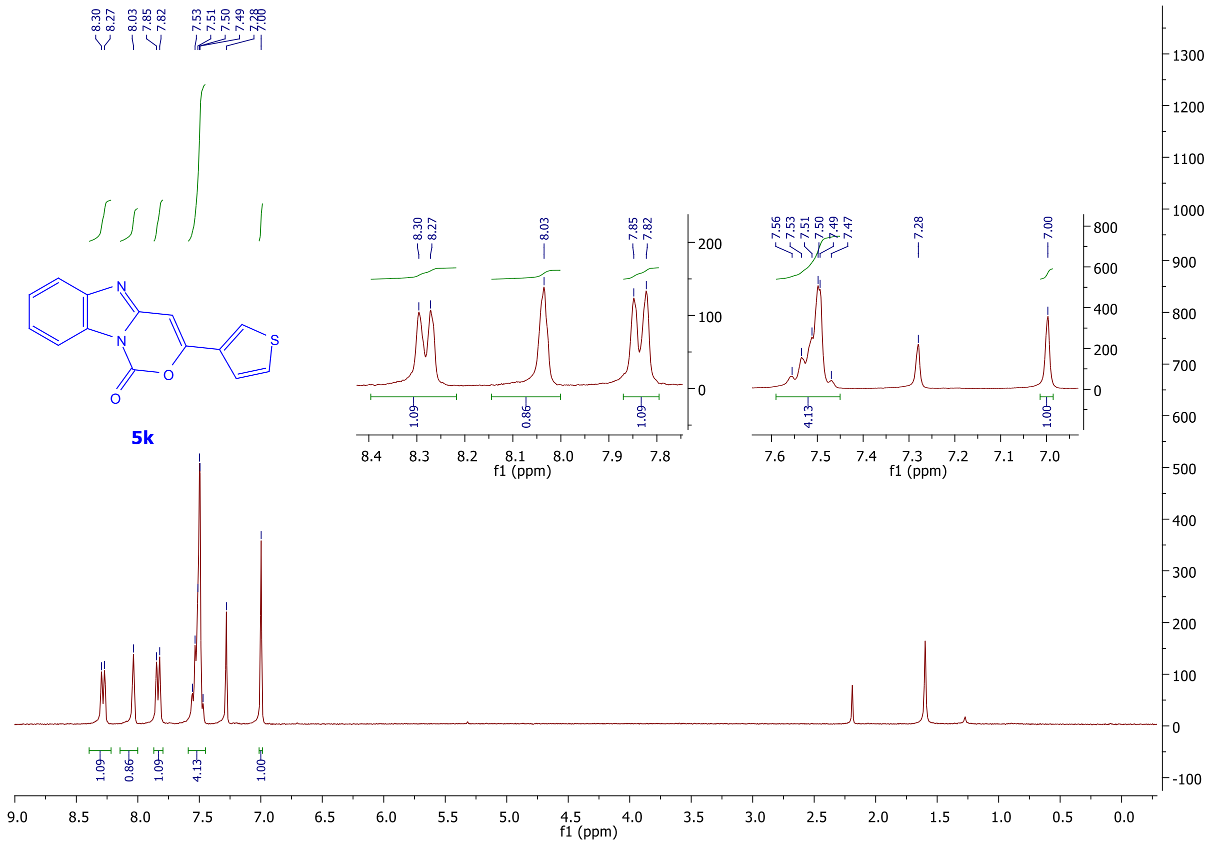

^{13}C NMR (75 MHz, $CDCl_3$) of **5k**

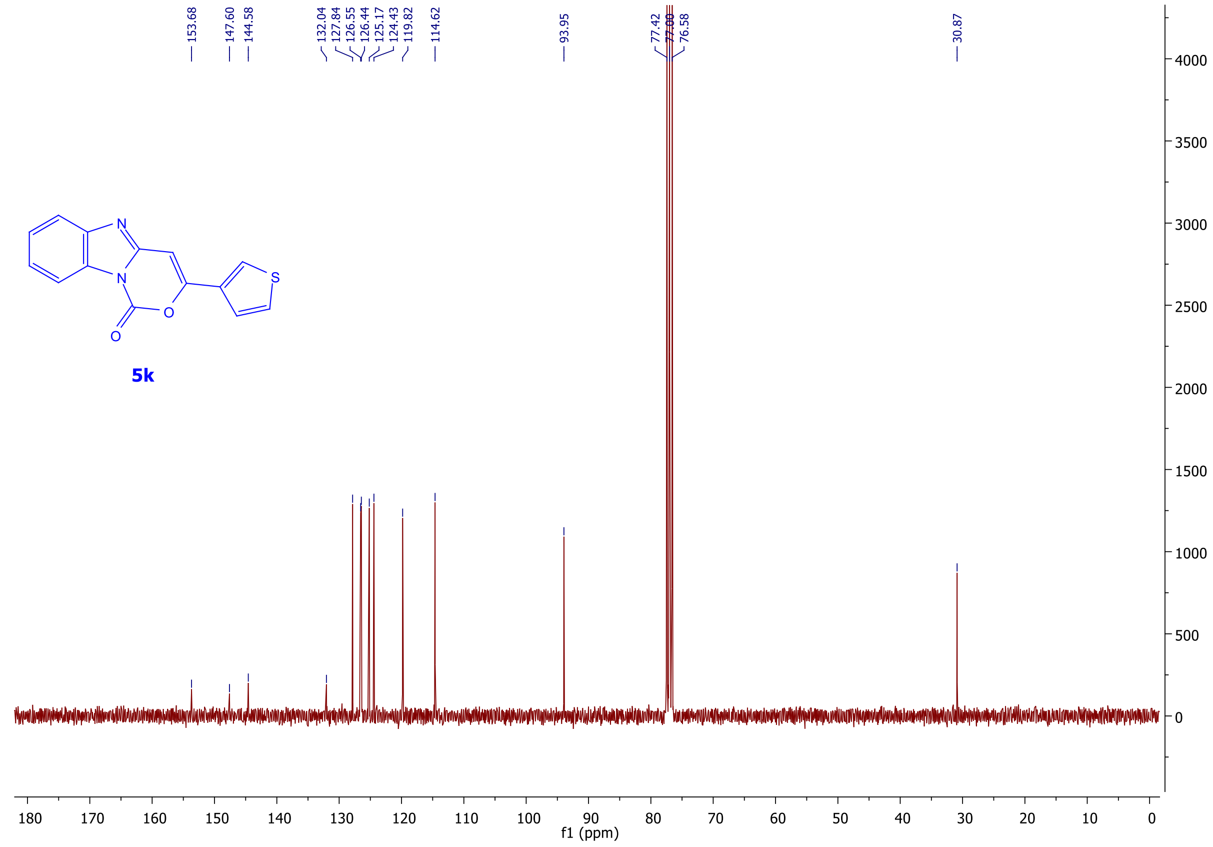

^{1}H NMR (300 MHz, $CDCl_3$) of **5l**

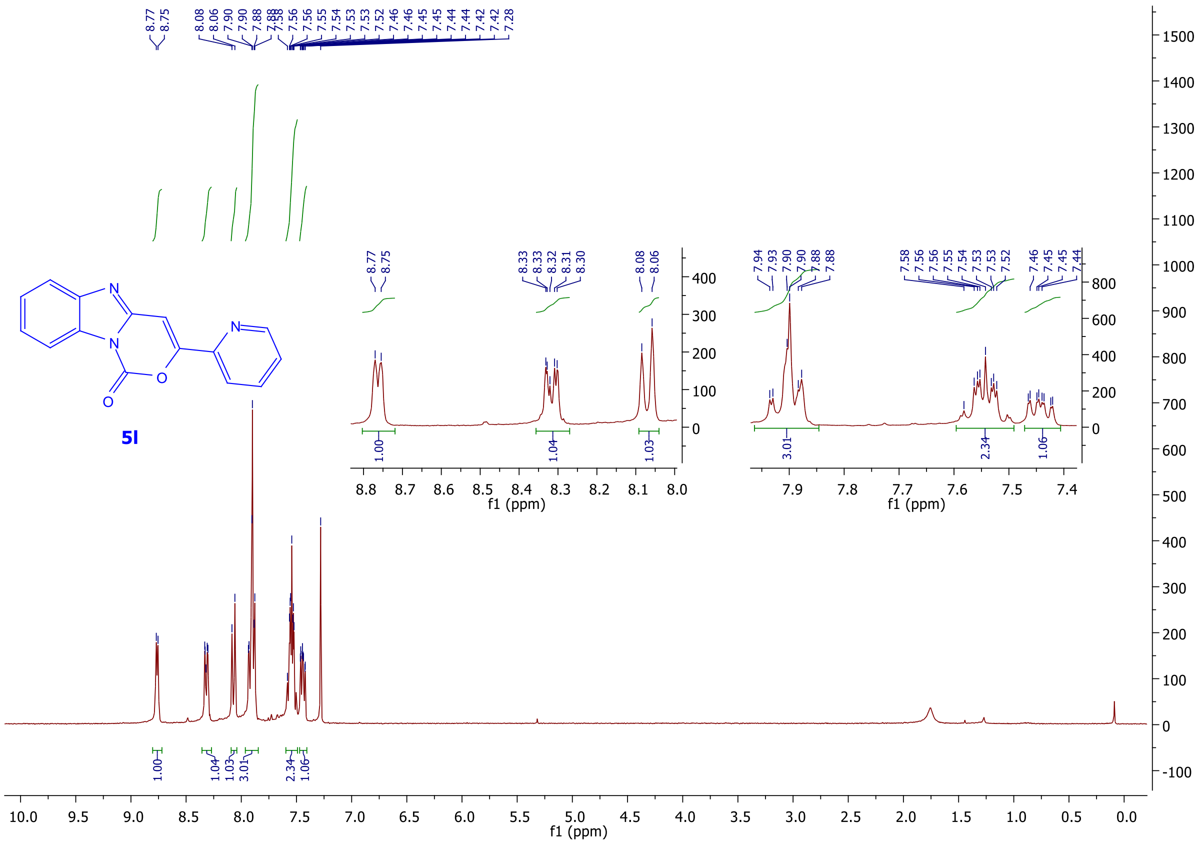

^{13}C NMR (75 MHz, $CDCl_3$) of **5l**

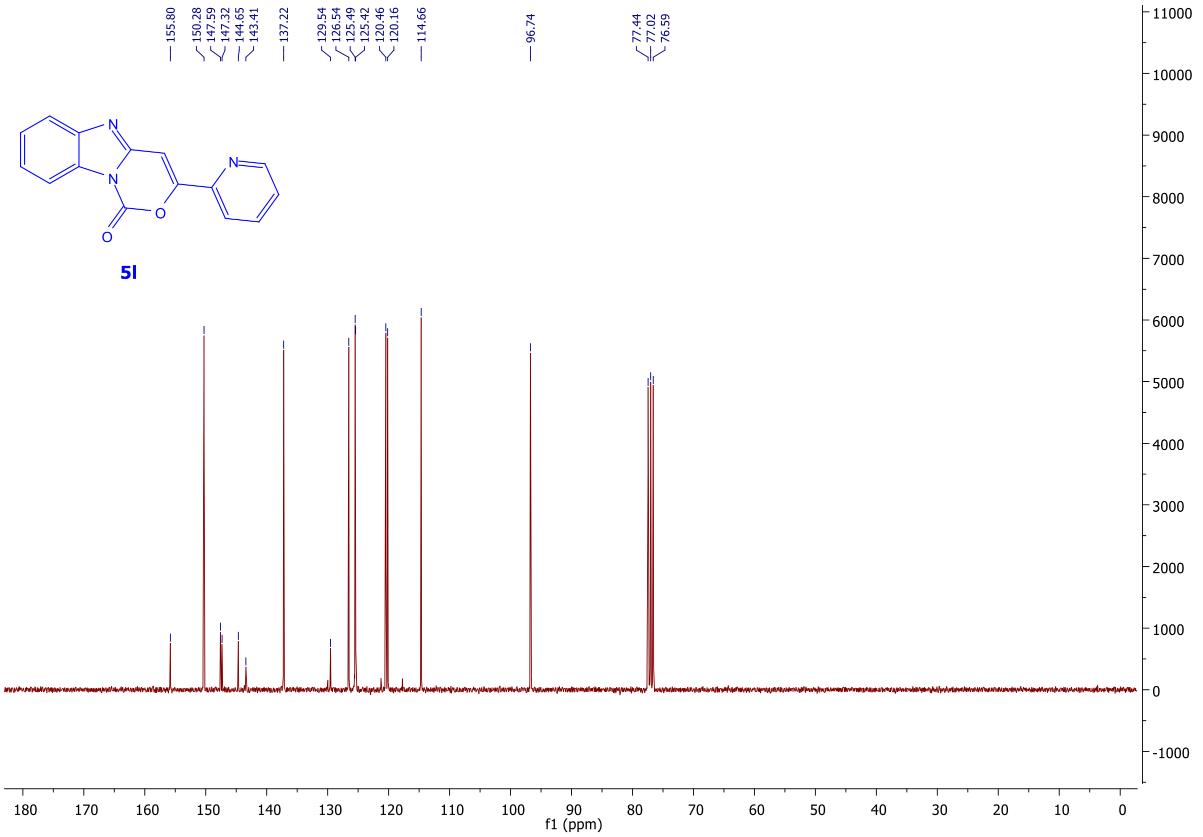

^{1}H NMR (300 MHz, $CDCl_3$) of **5m**

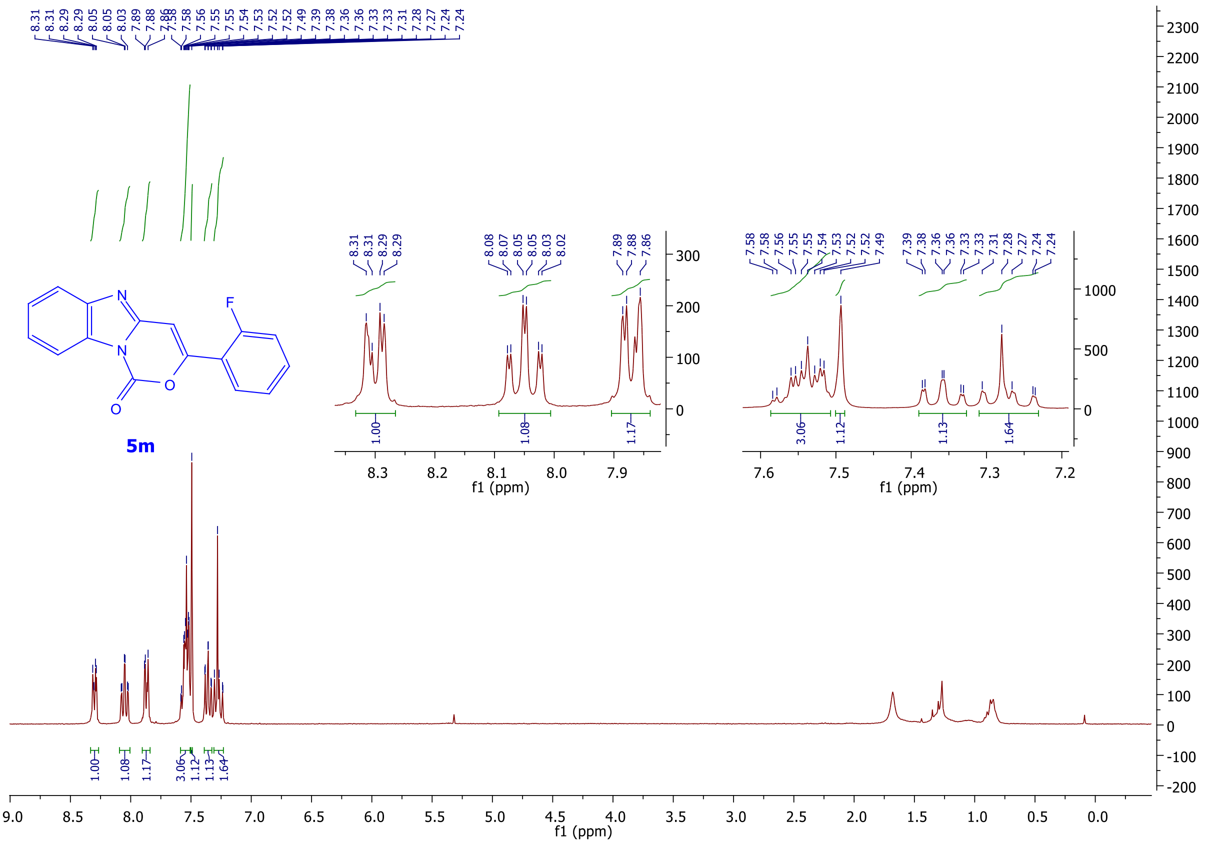

^{19}F NMR (282 MHz, $CDCl_3$) of **5m**

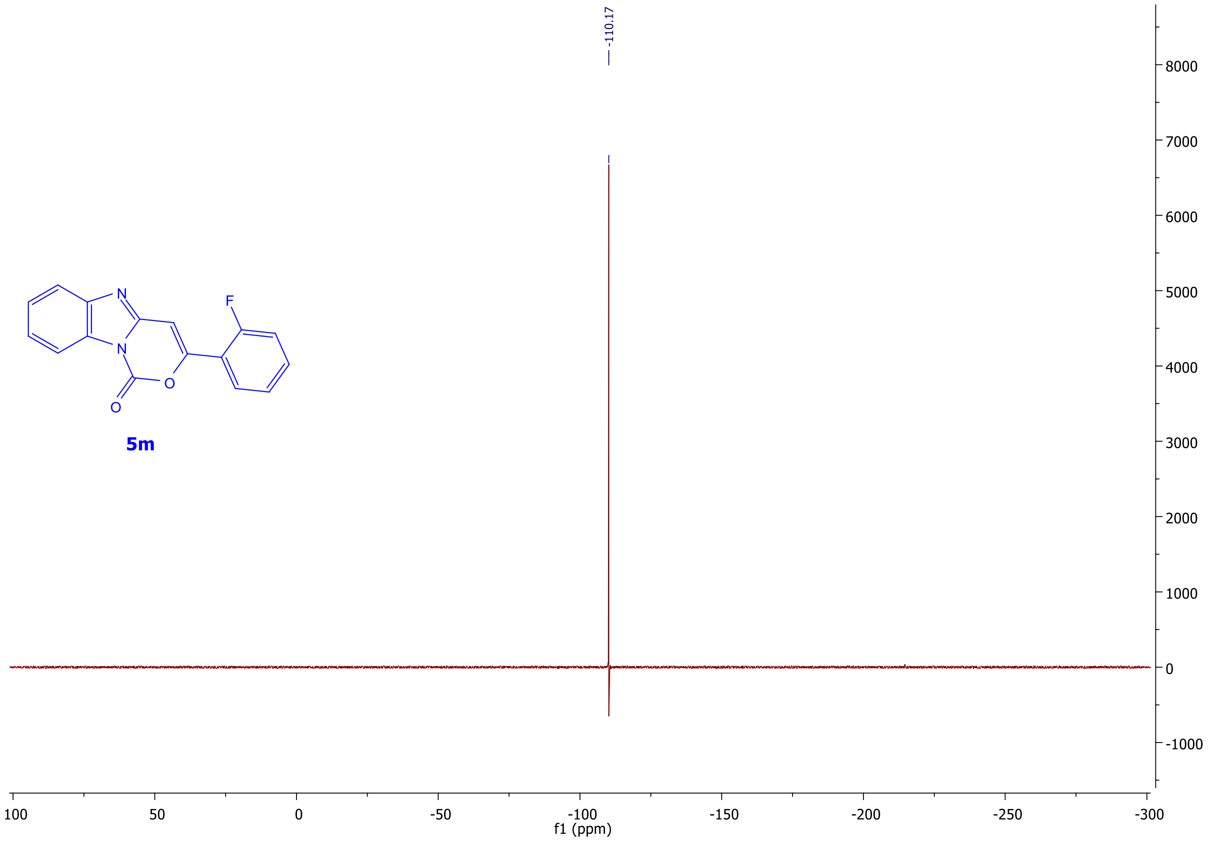

^{13}C NMR (75 MHz, $CDCl_3$) of **5m**

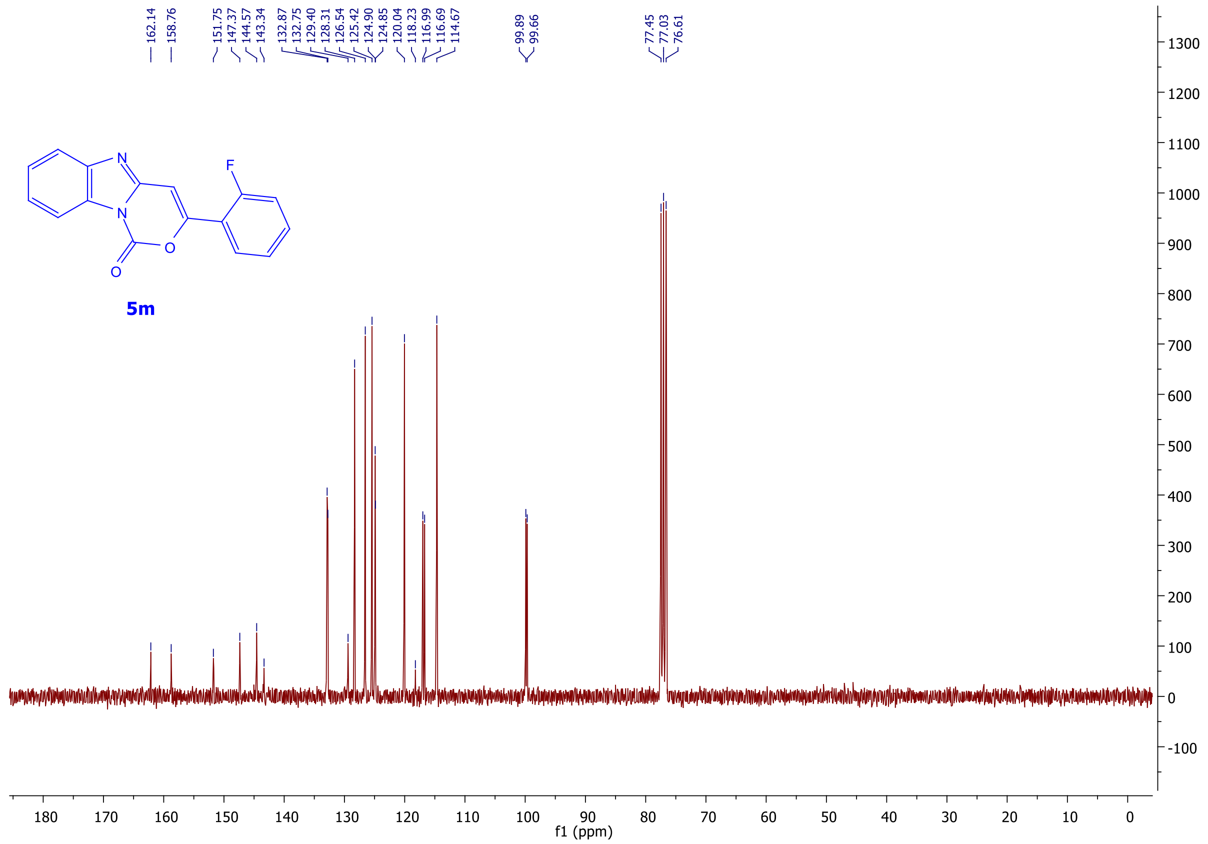

^{1}H NMR (300 MHz, $CDCl_3$) of **5n**

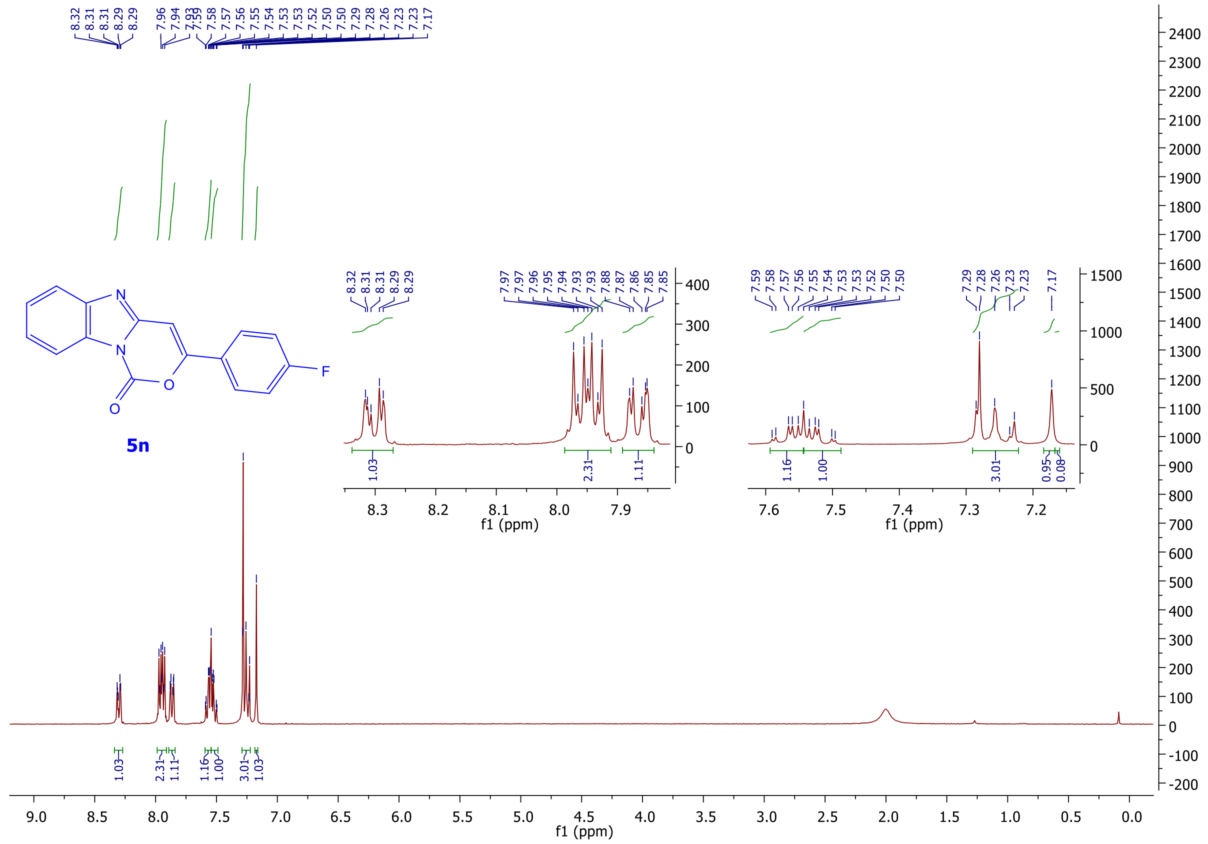

^{19}F NMR (282 MHz, $CDCl_3$) of **5n**

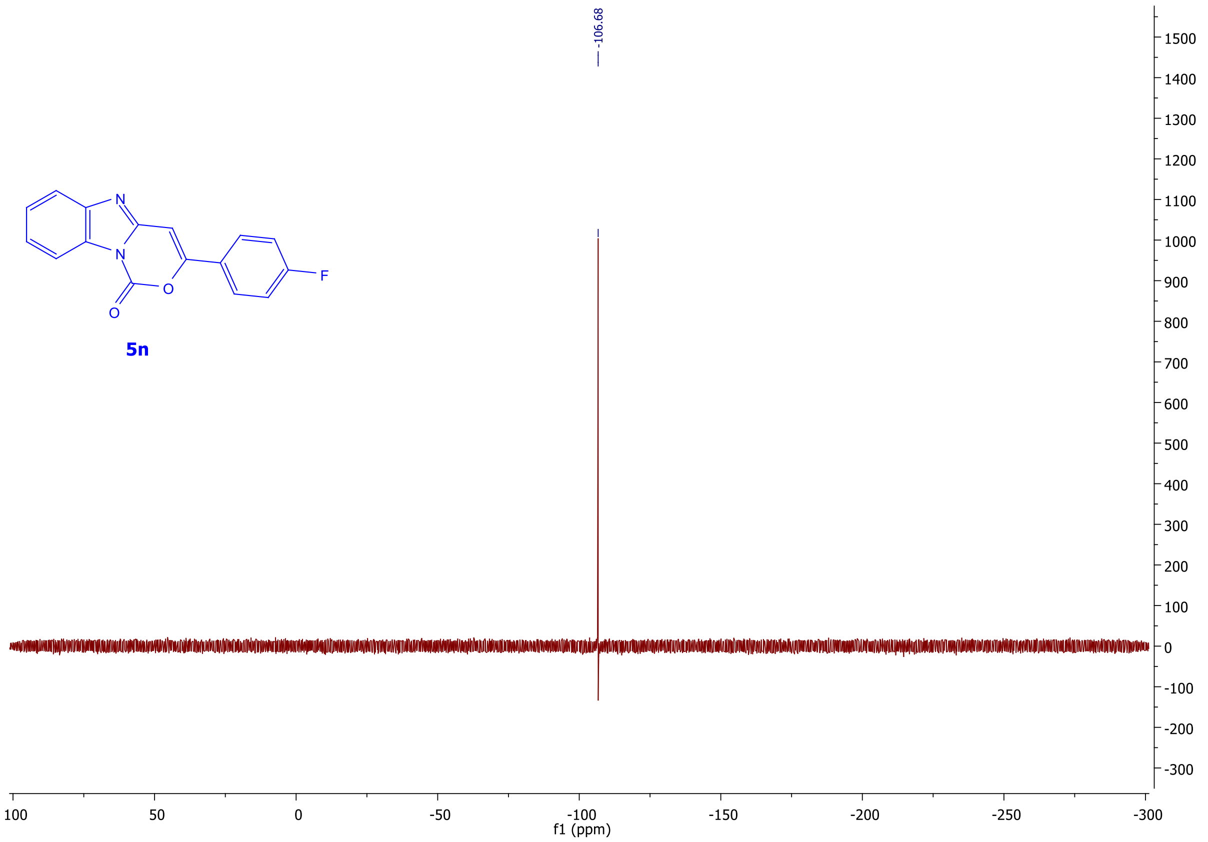

^{13}C NMR (75 MHz, DMSO-d_6) of **5n**

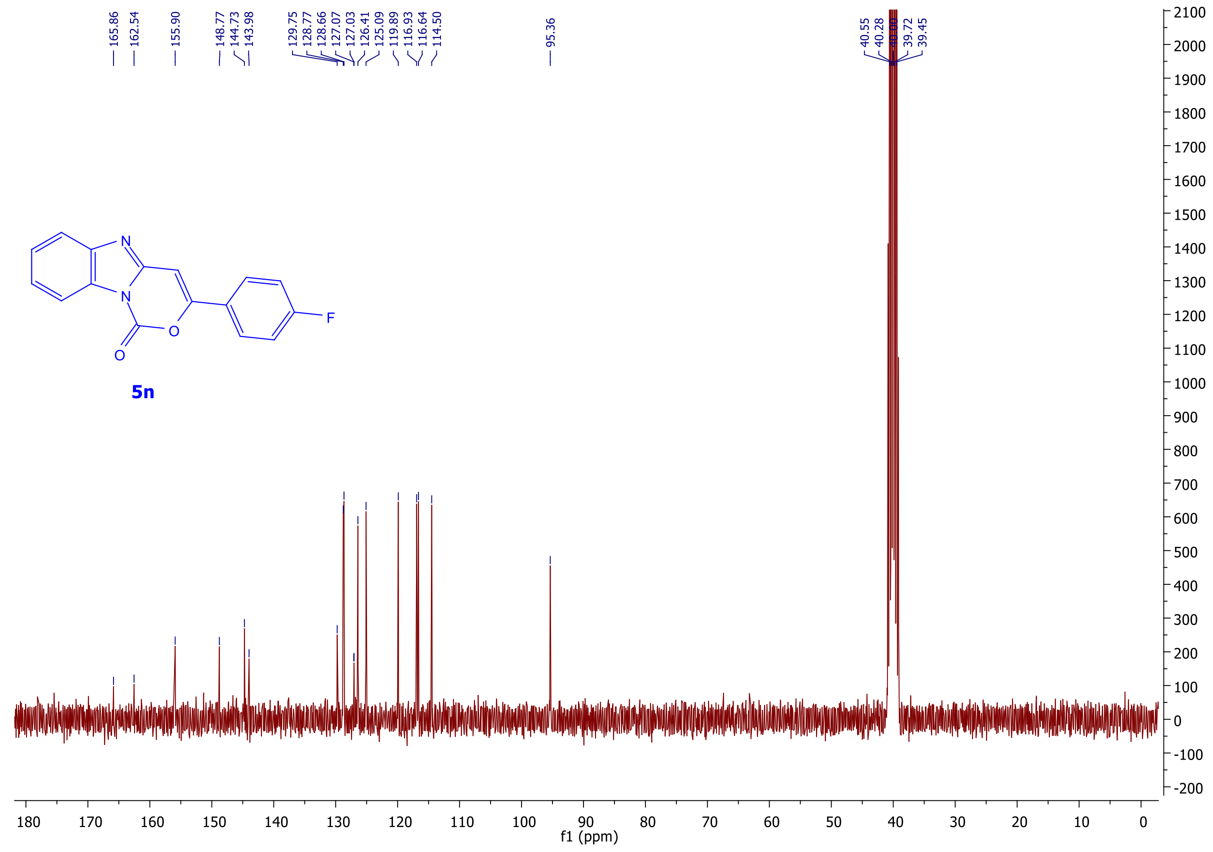

^{1}H NMR (300 MHz, $CDCl_3$) of **5o**

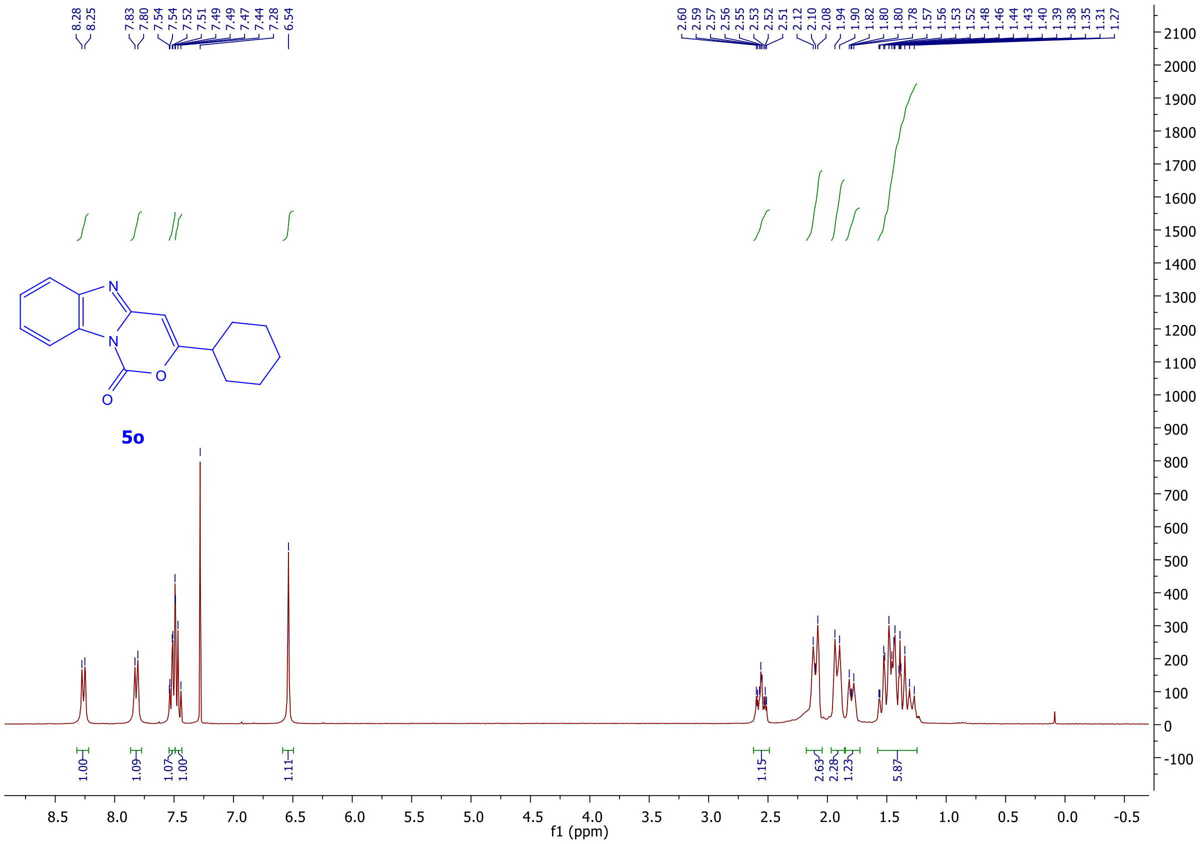

^{13}C NMR (75 MHz, $CDCl_3$) of **5o**

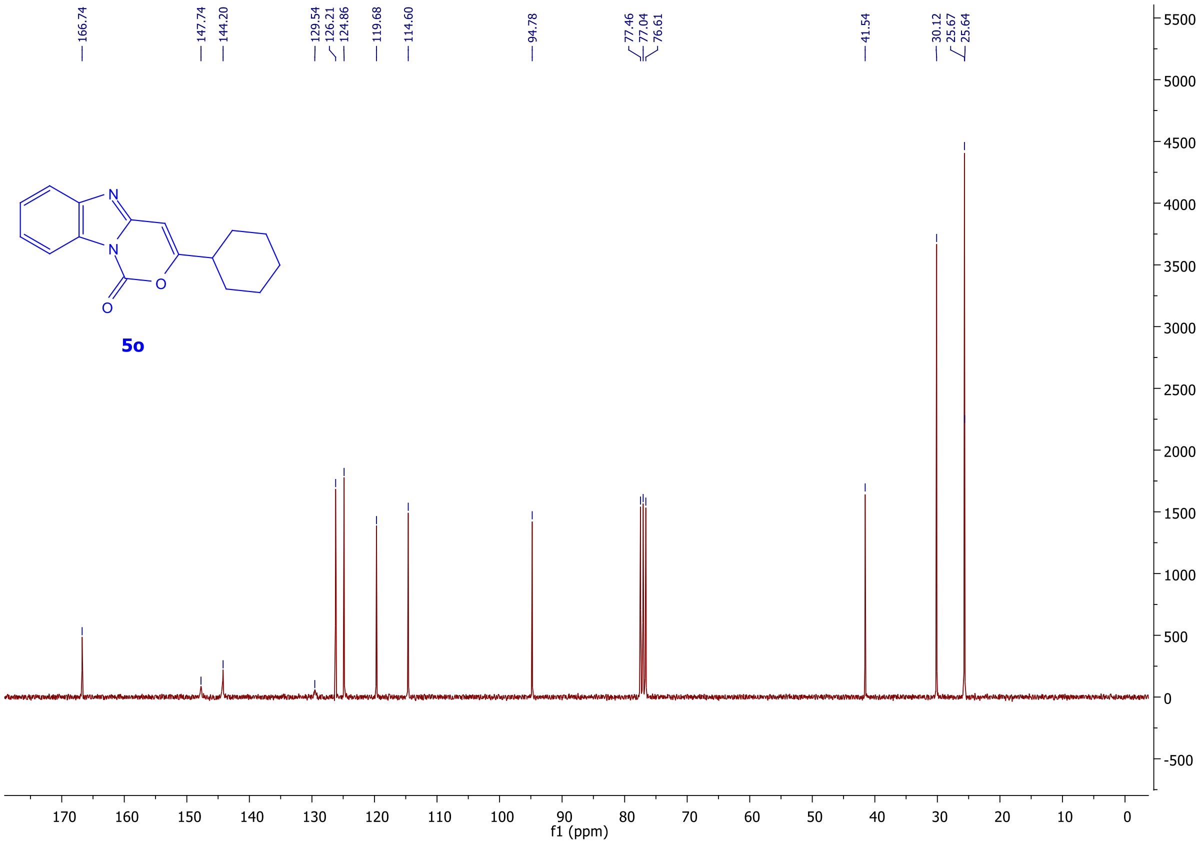

2. Details of DFT calculations

The structure of each studied species was optimized by using the Turbomole 7.4 program package.[1] Before their visualization using TmoleX (version 4.5.3), the structure of each individual species was optimized in the gas phase, with a convergence criterion of 10^{-8} Hartree, using the hybrid functional B3LYP and the triplet-ζ basis set 6-311+G*,[2] to collect its more stable 3D conformer. The stability of each structure was then investigated during further DFT calculations. During this step, the energy of each species was then minimized again using DFT calculations combining the Resolution of Identity (RI) approximation,[3,4] within the Turbomole 7.4 program package using the B3LYP function with the def2-TZVP basis set.[5–7] All minimum energy structures were obtained with full optimization, without constraints. Corrections for long range non-bonding interactions were given using the Grimme D3 dispersion model.[8] An implicit solvent model was additionally undertaken, using the COSMO Model implemented in Turbomole to determine thermodynamic and charge population properties in DCE. Analytical frequencies were then run on each structure at 1 atm and 298.15 K to calculate finally each energy.

2.1. Cartesian coordinates of the molecule 4a

	x	y	z
C	4.091520	1.523016	0.754366
C	2.956604	0.792659	0.407099
C	2.988477	-0.110663	-0.671854
C	4.137255	-0.312627	-1.428727
C	5.260214	0.424315	-1.068024
C	5.241390	1.327762	0.004462
N	1.695647	-0.655372	-0.729930
C	0.966147	-0.038203	0.325675
N	1.701268	0.809746	0.993133
C	-0.385864	-0.303908	0.625094
C	-1.534063	-0.480419	0.940105
C	-2.935651	-0.689389	1.261382
C	-3.884481	0.135883	0.373102
C	-5.354845	-0.105450	0.709541
H	4.057884	2.215499	1.584911
H	4.151533	-1.006951	-2.253229
H	6.174151	0.294175	-1.633919
H	6.140369	1.879924	0.247977
H	-3.108204	-0.437213	2.312810
H	-3.173066	-1.753770	1.155229
H	-3.644485	1.196516	0.486554
H	-3.697496	-0.113713	-0.675298
H	-5.529843	0.143055	1.761746
H	-5.578496	-1.172788	0.605694
O	0.021447	-1.944073	-1.497134
C	-0.654648	-2.927201	-2.373961
C	0.037946	-4.281244	-2.262373
C	-0.687234	-2.400891	-3.805087
C	-2.056105	-2.978844	-1.777459

C	1.290810	-1.608549	-1.680621
O	2.042781	-2.027959	-2.526666
H	1.038465	-4.251408	-2.686662
H	0.102041	-4.589000	-1.217204
H	-0.549126	-5.027333	-2.801422
H	0.310307	-2.360690	-4.236651
H	-1.305354	-3.062397	-4.415017
H	-1.128249	-1.402949	-3.830747
H	-2.670218	-3.682707	-2.340678
H	-2.524495	-1.995276	-1.816677
H	-2.015309	-3.302448	-0.737212
C	-6.302915	0.704828	-0.172705
H	-6.173142	0.449533	-1.227702
H	-7.346494	0.518840	0.089107
H	-6.118265	1.776906	-0.066648

2.2. Cartesian coordinates of the molecule 4b

	x	y	z
C	4.075269	1.367095	0.919766
C	2.929473	0.687216	0.509631
C	2.947603	-0.116076	-0.646577
C	4.093579	-0.266862	-1.419865
C	5.226831	0.418042	-0.995557
C	5.221424	1.222759	0.153565
N	1.646922	-0.632345	-0.752978
C	0.927960	-0.103106	0.353976
N	1.675535	0.669086	1.096837
C	-0.428476	-0.365457	0.629550
C	-1.581964	-0.528925	0.942842
H	4.051792	1.983114	1.808790
H	4.098391	-0.886921	-2.301665
H	6.138998	0.325566	-1.571547
H	6.128677	1.737395	0.443869
O	-0.035812	-1.856035	-1.603794
C	-0.694243	-2.843695	-2.494679
C	-0.001318	-4.194938	-2.357384
C	-0.691573	-2.324895	-3.928679
C	-2.108533	-2.893207	-1.932408
C	1.240824	-1.534632	-1.753614
O	2.000732	-1.926525	-2.605276
H	1.012138	-4.164290	-2.750185
H	0.028919	-4.500623	-1.310117
H	-0.568523	-4.943615	-2.913650
H	0.314289	-2.295399	-4.340989
H	-1.304421	-2.984161	-4.546385
H	-1.123464	-1.323425	-3.968796

H	-2.701170	-3.617096	-2.493294
H	-2.586626	-1.917371	-2.010429
H	-2.097183	-3.185379	-0.882671
C	-2.940861	-0.748595	1.291583
C	-3.281598	-1.186030	2.580879
C	-4.609247	-1.412275	2.913404
C	-5.614505	-1.203877	1.973122
C	-5.286016	-0.763724	0.693960
C	-3.961180	-0.536268	0.351871
H	-2.496726	-1.344722	3.308641
H	-4.861219	-1.751067	3.910586
H	-6.649620	-1.380983	2.236979
H	-6.065546	-0.594592	-0.038549
H	-3.701968	-0.186293	-0.638336

2.3. Cartesian coordinates of the molecule 4c

	x	y	z
C	4.119786	1.350593	0.872779
C	2.962328	0.690994	0.462349
C	2.982946	-0.161817	-0.657593
C	4.141786	-0.382167	-1.393691
C	5.286856	0.283345	-0.969483
C	5.279542	1.136375	0.143936
N	1.667442	-0.636005	-0.779540
C	0.937946	-0.037207	0.285362
N	1.694130	0.739832	1.015709
C	-0.425937	-0.260904	0.552505
C	-1.582916	-0.378359	0.876782
H	4.094779	2.005273	1.733726
H	4.148686	-1.042494	-2.245785
H	6.209634	0.135532	-1.516332
H	6.195890	1.633549	0.436276
O	-0.048676	-1.773550	-1.683548
C	-0.709918	-2.797353	-2.529839
C	-0.136329	-4.172145	-2.204618
C	-0.557554	-2.430695	-4.002194
C	-2.163010	-2.691754	-2.088365
C	1.258742	-1.565921	-1.751651
O	2.040690	-2.070087	-2.520731
H	0.904090	-4.252605	-2.511524
H	-0.210682	-4.369382	-1.133847
H	-0.714336	-4.933214	-2.732089
H	0.475596	-2.516140	-4.329529
H	-1.173593	-3.104169	-4.601402
H	-0.903067	-1.410029	-4.176070
H	-2.764998	-3.426092	-2.625226

H	-2.553809	-1.697531	-2.305044
H	-2.257323	-2.873476	-1.018093
C	-2.940168	-0.536910	1.254563
C	-3.288268	-0.748521	2.594018
C	-4.613103	-0.915181	2.974963
C	-5.622539	-0.867096	2.011268
C	-5.289485	-0.648962	0.669770
C	-3.971017	-0.486175	0.298256
H	-2.507436	-0.783897	3.342277
H	-4.844604	-1.078464	4.017325
H	-6.086316	-0.607648	-0.061118
H	-3.720957	-0.309521	-0.738720
C	-7.349291	-1.227141	3.623453
O	-6.947053	-1.017891	2.275567
H	-8.432766	-1.312932	3.599424
H	-7.065498	-0.382841	4.258725
H	-6.920570	-2.148675	4.028213

2.4. Cartesian coordinates of the molecule 4d

	x	y	z
C	4.074175	1.372341	0.921998
C	2.927906	0.693990	0.510767
C	2.943858	-0.102256	-0.650351
C	4.088011	-0.247411	-1.427350
C	5.221832	0.435965	-1.001892
C	5.218578	1.233739	0.151995
N	1.644094	-0.621196	-0.755062
C	0.927019	-0.098664	0.356613
N	1.675501	0.671142	1.101156
C	-0.430483	-0.358139	0.628634
C	-1.586356	-0.518546	0.934933
H	4.052462	1.982939	1.814815
H	4.090677	-0.861181	-2.313554
H	6.132481	0.348105	-1.581007
H	6.126038	1.747613	0.443052
O	-0.026867	-1.876307	-1.582425
C	-0.683991	-2.864343	-2.473325
C	0.038848	-4.203124	-2.369802
C	-0.721640	-2.325837	-3.899655
C	-2.084895	-2.950652	-1.882444
C	1.238444	-1.523679	-1.755639
O	1.989996	-1.891751	-2.625310
H	1.042974	-4.146418	-2.783194
H	0.097388	-4.522116	-1.327731
H	-0.525020	-4.955513	-2.924415
H	0.274844	-2.269047	-4.331405

H	-1.332623	-2.989677	-4.514271
H	-1.175672	-1.333557	-3.916831
H	-2.674240	-3.679105	-2.440941
H	-2.584609	-1.984287	-1.935597
H	-2.045016	-3.257038	-0.837353
C	-2.948026	-0.735925	1.274583
C	-3.293564	-1.170845	2.563060
C	-4.619366	-1.401369	2.915351
C	-5.612163	-1.181565	1.956553
C	-5.284721	-0.744928	0.677908
C	-3.960934	-0.521063	0.329806
H	-2.505733	-1.326218	3.289761
H	-6.651013	-1.349592	2.216944
H	-6.067875	-0.572369	-0.050133
H	-3.700478	-0.170346	-0.659616
C	-4.975473	-1.899002	4.291725
H	-4.182259	-1.685024	5.008807
H	-5.895954	-1.437549	4.653587
H	-5.132936	-2.981559	4.285041

2.5. Cartesian coordinates of the molecule 4e

	x	y	z
C	4.058620	1.383565	0.912879
C	2.916792	0.698344	0.500550
C	2.948653	-0.125789	-0.640494
C	4.103876	-0.291877	-1.396509
C	5.233096	0.398565	-0.970226
C	5.214389	1.223533	0.164236
N	1.646861	-0.636536	-0.758358
C	0.914224	-0.087764	0.330348
N	1.655143	0.693253	1.071195
C	-0.439582	-0.361020	0.606184
C	-1.589093	-0.533554	0.930475
H	4.024954	2.015749	1.790156
H	4.119850	-0.930724	-2.264728
H	6.152673	0.293314	-1.532089
H	6.118900	1.741497	0.457258
O	-0.050546	-1.788369	-1.678613
C	-0.699186	-2.799801	-2.549691
C	-0.094688	-4.171399	-2.271233
C	-0.567478	-2.385362	-4.011231
C	-2.149884	-2.737101	-2.092269
C	1.250990	-1.549766	-1.752244
O	2.037499	-2.014327	-2.541041
H	0.944602	-4.220647	-2.588262
H	-0.156280	-4.402990	-1.206563

H	-0.660978	-4.928000	-2.817599
H	0.464101	-2.439904	-4.350116
H	-1.175417	-3.052394	-4.625666
H	-0.934759	-1.366991	-4.150630
H	-2.742602	-3.463730	-2.649580
H	-2.562193	-1.743654	-2.269259
H	-2.231126	-2.958120	-1.028432
C	-2.940536	-0.761453	1.299336
C	-3.269700	-1.132564	2.611921
C	-4.589517	-1.362429	2.965131
C	-5.623583	-1.230436	2.034537
C	-5.291686	-0.850313	0.732447
C	-3.975604	-0.619125	0.364124
H	-2.480514	-1.235049	3.345260
H	-4.823659	-1.646749	3.984749
H	-6.078005	-0.729310	-0.003935
H	-3.736968	-0.316404	-0.646486
C	-7.050288	-1.513679	2.419966
H	-7.246298	-1.229017	3.454960
H	-7.749420	-0.974981	1.778972
H	-7.273560	-2.580936	2.326796

2.6. Cartesian coordinates of the molecule 4f

	x	y	z
C	3.932539	1.821925	0.484632
C	2.833411	1.002294	0.230839
C	2.945391	-0.091965	-0.649177
C	4.136683	-0.399421	-1.297033
C	5.220915	0.428793	-1.031688
C	5.124157	1.521328	-0.155977
N	1.667763	-0.666276	-0.680834
C	0.871576	0.109875	0.198697
N	1.547788	1.090335	0.737635
C	-0.463389	-0.193812	0.529663
C	-1.590442	-0.435062	0.882356
H	3.840064	2.660295	1.162159
H	4.212245	-1.245626	-1.961352
H	6.168034	0.220137	-1.513101
H	5.997322	2.136752	0.020354
O	-0.012061	-1.832389	-1.606265
C	-0.667818	-2.922472	-2.374236
C	-0.289872	-4.268820	-1.768404
C	-0.285700	-2.809804	-3.846032
C	-2.146355	-2.620693	-2.166517
C	1.305382	-1.740797	-1.518847
O	2.132730	-2.424670	-2.069180

H	0.765958	-4.487929	-1.911553
H	-0.520313	-4.279802	-0.702435
H	-0.876663	-5.052624	-2.251123
H	0.768868	-3.023711	-4.002524
H	-0.877350	-3.524313	-4.421392
H	-0.506295	-1.808058	-4.219231
H	-2.747623	-3.348818	-2.713008
H	-2.389013	-1.622570	-2.532278
H	-2.404711	-2.677464	-1.111076
C	-2.925493	-0.714275	1.261593
C	-3.333691	-1.996094	1.665179
C	-4.647966	-2.258808	2.019565
C	-5.587213	-1.235327	1.981311
C	-5.208829	0.046420	1.591607
C	-3.894950	0.303090	1.237160
H	-4.927098	-3.257232	2.326710
H	-6.612507	-1.442195	2.260160
H	-5.938400	0.845386	1.564888
H	-3.588720	1.294932	0.932823
Cl	-2.161786	-3.304556	1.741047

2.7. Cartesian coordinates of the molecule 4g

	x	y	z
C	4.079047	1.366391	0.919592
C	2.932364	0.687790	0.509438
C	2.948098	-0.112878	-0.648771
C	4.092760	-0.262591	-1.424345
C	5.226634	0.420835	-0.999948
C	5.223530	1.223182	0.151112
N	1.647818	-0.630170	-0.753026
C	0.932058	-0.103700	0.355971
N	1.679695	0.667676	1.099169
C	-0.425752	-0.361827	0.631251
C	-1.580003	-0.522016	0.941718
H	4.057374	1.979997	1.810292
H	4.095716	-0.879718	-2.308208
H	6.137942	0.329324	-1.577400
H	6.131512	1.736783	0.440794
O	-0.031478	-1.869640	-1.586556
C	-0.694037	-2.854500	-2.478380
C	0.014215	-4.200105	-2.366221
C	-0.718020	-2.319810	-3.906294
C	-2.099029	-2.924147	-1.895052
C	1.238701	-1.531569	-1.753944
O	1.991186	-1.908847	-2.618208
H	1.020981	-4.155312	-2.774517

H	0.063868	-4.516576	-1.322936
H	-0.554341	-4.948416	-2.921509
H	0.281320	-2.275073	-4.332811
H	-1.332504	-2.978988	-4.522376
H	-1.161225	-1.322747	-3.929238
H	-2.693217	-3.647251	-2.455212
H	-2.587740	-1.952278	-1.954196
H	-2.068309	-3.229431	-0.849327
C	-2.941194	-0.738881	1.281833
C	-3.279116	-1.199262	2.563394
C	-4.610205	-1.419482	2.873930
C	-5.622794	-1.193194	1.950185
C	-5.282536	-0.731181	0.682889
C	-3.957411	-0.503207	0.344118
H	-2.505899	-1.379619	3.296303
H	-6.654029	-1.373118	2.220344
H	-6.063923	-0.546039	-0.043270
H	-3.695554	-0.136536	-0.638699
Cl	-5.028818	-2.001970	4.483271

2.8. Cartesian coordinates of the molecule 4h

	x	y	z
C	4.070763	1.361151	0.927179
C	2.925848	0.681815	0.513319
C	2.945787	-0.118127	-0.645260
C	4.092821	-0.266118	-1.417604
C	5.224921	0.418127	-0.989618
C	5.217729	1.219527	0.162004
N	1.645340	-0.634137	-0.755401
C	0.925293	-0.108684	0.351709
N	1.671173	0.661302	1.098596
C	-0.431064	-0.372262	0.627149
C	-1.583673	-0.533870	0.944261
H	4.045859	1.974534	1.817956
H	4.099378	-0.883813	-2.301037
H	6.137927	0.327859	-1.564582
H	6.124432	1.733614	0.454856
O	-0.038398	-1.849536	-1.616851
C	-0.693372	-2.839076	-2.509188
C	-0.006799	-4.192088	-2.359548
C	-0.677366	-2.327117	-3.945452
C	-2.112399	-2.880516	-1.958442
C	1.241016	-1.534691	-1.758630
O	2.004229	-1.930058	-2.605312
H	1.010098	-4.166976	-2.743742
H	0.013280	-4.493344	-1.310754

H	-0.571677	-4.941232	-2.917495
H	0.331533	-2.304857	-4.350586
H	-1.289132	-2.986168	-4.564433
H	-1.103628	-1.323585	-3.993795
H	-2.702871	-3.606128	-2.519230
H	-2.586267	-1.903482	-2.048520
H	-2.110662	-3.165791	-0.906728
C	-2.940248	-0.750891	1.298833
C	-3.281396	-1.158142	2.597590
C	-4.604411	-1.385192	2.943407
C	-5.597329	-1.201823	1.989137
C	-5.288872	-0.791533	0.698240
C	-3.964268	-0.567036	0.357704
H	-2.499498	-1.296338	3.332336
H	-4.865739	-1.701009	3.943959
H	-6.077028	-0.647869	-0.027966
H	-3.713177	-0.239857	-0.641943
Cl	-7.277526	-1.492112	2.423766

2.9. Cartesian coordinates of the molecule 4i

	x	y	z
C	4.068324	1.420249	0.890798
C	2.926498	0.727372	0.490689
C	2.947754	-0.090802	-0.655240
C	4.093466	-0.244650	-1.428557
C	5.222013	0.453341	-1.014505
C	5.213450	1.273075	0.124514
N	1.651091	-0.618261	-0.751997
C	0.932846	-0.080496	0.348679
N	1.673974	0.707166	1.080633
C	-0.423602	-0.343468	0.629112
C	-1.575883	-0.508137	0.943000
H	4.042400	2.046862	1.772221
H	4.101223	-0.875588	-2.302593
H	6.134017	0.359666	-1.590462
H	6.118077	1.796725	0.406387
O	-0.023846	-1.873889	-1.569447
C	-0.682791	-2.873862	-2.447737
C	0.029155	-4.215517	-2.315471
C	-0.707529	-2.359856	-3.883080
C	-2.088039	-2.939080	-1.864121
C	1.246042	-1.535168	-1.740758
O	2.000164	-1.922041	-2.598696
H	1.035813	-4.173720	-2.724276
H	0.079717	-4.516831	-1.267776
H	-0.536798	-4.973577	-2.860042

H	0.291941	-2.318248	-4.309529
H	-1.319227	-3.029869	-4.490129
H	-1.153731	-1.364554	-3.921118
H	-2.680203	-3.671192	-2.414465
H	-2.579001	-1.969055	-1.937978
H	-2.056467	-3.230643	-0.814456
C	-2.936933	-0.729519	1.279533
C	-3.283504	-1.209337	2.548807
C	-4.619130	-1.428401	2.837606
C	-5.632927	-1.188704	1.920174
C	-5.283851	-0.707505	0.664491
C	-3.953435	-0.478407	0.343492
H	-2.527972	-1.408760	3.293589
H	-6.660152	-1.376042	2.195387
H	-6.056377	-0.507706	-0.066514
H	-3.684631	-0.097214	-0.632286
N	-4.979475	-1.944015	4.181100
O	-4.070182	-2.148734	4.973100
O	-6.165800	-2.134221	4.414057

2.10. Cartesian coordinates of the molecule 4j

	x	y	z
C	4.116985	1.353315	0.879088
C	2.957949	0.689616	0.479111
C	2.957966	-0.125265	-0.669399
C	4.098821	-0.305098	-1.444128
C	5.244915	0.364071	-1.030019
C	5.257523	1.180705	0.110927
N	1.649803	-0.623997	-0.763478
C	0.944985	-0.071354	0.339284
N	1.706578	0.697748	1.071276
C	-0.420027	-0.289660	0.610746
C	-1.581098	-0.409520	0.915680
H	4.107368	1.978294	1.762074
H	4.089127	-0.931840	-2.321173
H	6.153289	0.250476	-1.608182
H	6.174659	1.682265	0.392755
O	-0.029061	-1.904878	-1.532936
C	-0.703197	-2.902767	-2.402333
C	0.029974	-4.236497	-2.308014
C	-0.778613	-2.372773	-3.830157
C	-2.088515	-2.993262	-1.776652
C	1.227892	-1.543192	-1.743251
O	1.962351	-1.916299	-2.624596
H	1.023285	-4.176864	-2.746325
H	0.115717	-4.548825	-1.265832

H	-0.542918	-4.995407	-2.844066
H	0.206510	-2.313530	-4.286818
H	-1.400816	-3.043577	-4.425529
H	-1.238652	-1.383168	-3.842484
H	-2.685900	-3.731331	-2.313322
H	-2.597124	-2.031600	-1.827910
H	-2.020718	-3.290098	-0.730085
C	-2.947819	-0.581083	1.252908
C	-3.318745	-0.932161	2.560684
C	-4.652131	-1.115688	2.884588
C	-5.641894	-0.950781	1.912317
C	-5.278971	-0.593543	0.611624
C	-3.948724	-0.408885	0.282734
H	-2.550150	-1.059021	3.311288
H	-4.934306	-1.388078	3.891517
H	-6.056953	-0.463327	-0.128652
H	-3.667740	-0.123158	-0.721810
O	-7.310621	-1.470062	3.496761
C	-7.086859	-1.140062	2.207561
O	-7.972365	-1.015837	1.392892
C	-8.687590	-1.669731	3.859913
H	-8.674630	-1.921318	4.916726
H	-9.262518	-0.760131	3.687846
H	-9.121553	-2.481388	3.276320

2.11. Cartesian coordinates of the molecule 4k

	x	y	z
C	1.293667	0.427785	2.268154
C	0.175244	-0.308555	1.880241
C	0.244620	-1.195324	0.788839
C	1.414489	-1.374600	0.059050
C	2.520054	-0.632974	0.460753
C	2.464118	0.254417	1.545905
N	-1.042590	-1.744747	0.682562
C	-1.804582	-1.153956	1.727303
N	-1.094140	-0.312662	2.432382
C	-3.150802	-1.449218	2.014541
C	-4.291522	-1.615966	2.372169
H	1.231277	1.107947	3.107089
H	1.459211	-2.060983	-0.771042
H	3.450399	-0.747055	-0.081273
H	3.351268	0.810644	1.821299
O	-2.702810	-2.971128	-0.207596
C	-3.317149	-4.027910	-1.049167
C	-2.698893	-5.377630	-0.702251
C	-3.162255	-3.672029	-2.523911

C	-4.777566	-3.968255	-0.623733
C	-1.403955	-2.712934	-0.270652
O	-0.596008	-3.201422	-1.022242
H	-1.653498	-5.424664	-0.998818
H	-2.776224	-5.565271	0.370022
H	-3.244544	-6.164782	-1.225994
H	-2.123346	-3.723991	-2.839565
H	-3.746955	-4.374015	-3.121696
H	-3.542268	-2.666477	-2.713268
H	-5.350045	-4.722189	-1.165597
H	-5.195598	-2.986205	-0.847071
H	-4.876165	-4.152789	0.445779
S	-7.701246	-2.087451	4.306449
C	-7.909822	-2.214157	2.581947
C	-6.736121	-2.052497	1.918618
C	-5.623217	-1.819115	2.798614
C	-6.005196	-1.809697	4.120475
H	-8.889451	-2.394959	2.170526
H	-6.641501	-2.085199	0.844240
H	-5.374104	-1.654636	4.979846

2.12. Cartesian coordinates of the molecule 4l

	x	y	z
C	1.183627	-0.028749	2.532911
C	0.025350	-0.665759	2.086653
C	0.007181	-1.325994	0.841367
C	1.123728	-1.373465	0.013649
C	2.267151	-0.732768	0.475098
C	2.299757	-0.070476	1.713102
N	-1.281072	-1.860722	0.731773
C	-1.966215	-1.470234	1.907193
N	-1.206053	-0.766824	2.708487
C	-3.332691	-1.708055	2.142938
C	-4.506763	-1.834004	2.386064
H	1.191903	0.476482	3.489493
H	1.092865	-1.871382	-0.942616
H	3.156841	-0.741787	-0.141818
H	3.214426	0.415581	2.028183
O	-2.800424	-3.338237	-0.002021
C	-3.557800	-4.155991	-0.989427
C	-2.664681	-5.270457	-1.524272
C	-4.084417	-3.241783	-2.089108
C	-4.694647	-4.716315	-0.146558
C	-1.721033	-2.658397	-0.347481
O	-1.128385	-2.684370	-1.397697
H	-1.854558	-4.879319	-2.135018

H	-2.243414	-5.846508	-0.698488
H	-3.270236	-5.944670	-2.132898
H	-3.274304	-2.825637	-2.684848
H	-4.737484	-3.817515	-2.747493
H	-4.669958	-2.430129	-1.654528
H	-5.320230	-5.359429	-0.768283
H	-5.310803	-3.917202	0.264610
H	-4.298802	-5.312740	0.676971
C	-5.889596	-1.971708	2.689596
C	-6.369151	-1.609230	3.956454
C	-7.721885	-1.750302	4.221344
C	-8.554394	-2.243696	3.223802
C	-7.988225	-2.576828	1.996314
N	-6.692975	-2.450440	1.720100
H	-5.682293	-1.227792	4.699479
H	-8.119963	-1.478491	5.190945
H	-9.616643	-2.369147	3.387333
H	-8.606885	-2.964745	1.193690

2.13. Cartesian coordinates of the molecule 4m

	x	y	z
C	4.144966	1.160227	1.047838
C	2.973233	0.543506	0.609677
C	2.934624	-0.113006	-0.636566
C	4.043060	-0.177043	-1.473425
C	5.200970	0.443685	-1.019929
C	5.254023	1.102364	0.218766
N	1.636379	-0.627761	-0.736246
C	0.966792	-0.227041	0.445628
N	1.744251	0.461457	1.241375
C	-0.404188	-0.439359	0.684799
C	-1.580800	-0.538283	0.928857
H	4.169104	1.663328	2.005283
H	3.996376	-0.672335	-2.430491
H	6.085213	0.421781	-1.644308
H	6.178786	1.573035	0.527696
O	0.117731	-2.128775	-1.425330
C	-0.628153	-2.984166	-2.385363
C	0.279600	-4.097114	-2.898686
C	-1.183994	-2.111165	-3.504104
C	-1.744767	-3.548969	-1.517203
C	1.188142	-1.443743	-1.795677
O	1.765910	-1.485638	-2.853491
H	1.074503	-3.707327	-3.529722
H	0.722223	-4.641022	-2.062345
H	-0.319124	-4.799803	-3.481368

H	-0.386407	-1.696656	-4.117417
H	-1.830559	-2.717347	-4.141281
H	-1.780752	-1.298856	-3.087159
H	-2.366128	-4.218827	-2.113304
H	-2.369206	-2.747987	-1.128298
H	-1.330874	-4.110542	-0.679005
C	-2.960697	-0.663322	1.215647
C	-3.429470	-0.794065	2.534218
C	-4.784163	-0.919421	2.798667
C	-5.705167	-0.915125	1.753715
C	-5.270049	-0.782725	0.439147
C	-3.917032	-0.658511	0.194092
H	-2.706341	-0.794602	3.338540
H	-5.123778	-1.019915	3.821143
H	-6.763491	-1.012235	1.958655
H	-5.961449	-0.770867	-0.392749
F	-3.499847	-0.525408	-1.084245

2.14. Cartesian coordinates of the molecule 4n

	x	y	z
C	4.124816	1.274748	0.997250
C	2.962537	0.668159	0.523139
C	2.983198	-0.093237	-0.661038
C	4.147003	-0.272789	-1.400786
C	5.296424	0.338149	-0.912353
C	5.289136	1.100253	0.265625
N	1.662517	-0.534578	-0.834534
C	0.931833	-0.014442	0.268062
N	1.689125	0.689878	1.066681
C	-0.435583	-0.252753	0.509392
C	-1.592967	-0.410332	0.810828
H	4.099967	1.858888	1.907483
H	4.154127	-0.863998	-2.302217
H	6.223479	0.219168	-1.458905
H	6.209793	1.556989	0.606188
O	-0.070329	-1.521347	-1.873844
C	-0.748816	-2.436644	-2.825997
C	-0.239319	-3.857746	-2.614462
C	-0.541617	-1.943778	-4.253812
C	-2.208107	-2.310707	-2.410689
C	1.245451	-1.362275	-1.893107
O	2.028525	-1.827845	-2.684286
H	0.805064	-3.954110	-2.902578
H	-0.351135	-4.148042	-1.568359
H	-0.832278	-4.543078	-3.222935
H	0.495882	-2.042310	-4.562981

H	-1.166954	-2.533242	-4.927154
H	-0.841533	-0.897884	-4.341136
H	-2.825077	-2.960110	-3.033287
H	-2.550723	-1.283264	-2.534907
H	-2.340243	-2.595706	-1.367342
C	-2.953363	-0.629088	1.151577
C	-3.298589	-1.098009	2.429151
C	-4.623553	-1.335639	2.761221
C	-5.601050	-1.098068	1.809010
C	-5.300538	-0.625949	0.542140
C	-3.973255	-0.391412	0.217216
H	-2.517505	-1.276341	3.155865
H	-4.904440	-1.699525	3.740372
H	-6.097087	-0.446652	-0.167463
H	-3.715360	-0.018250	-0.763952
F	-6.892492	-1.333853	2.127035

2.15. Cartesian coordinates of the molecule 4o

	x	y	z
C	1.222609	0.634467	2.338269
C	0.078624	-0.070334	1.968754
C	0.086051	-0.903544	0.834313
C	1.218754	-1.059750	0.043241
C	2.351265	-0.349469	0.427100
C	2.356681	0.484582	1.554449
N	-1.209868	-1.439752	0.767157
C	-1.916212	-0.889596	1.874332
N	-1.164723	-0.088131	2.580039
C	-3.261609	-1.173910	2.185482
C	-4.403817	-1.373010	2.510159
H	1.207947	1.273388	3.211246
H	1.214259	-1.701090	-0.823264
H	3.253416	-0.445657	-0.163925
H	3.262139	1.018463	1.814192
O	-2.911095	-2.654042	-0.056763
C	-3.612920	-3.575118	-0.978864
C	-2.941211	-4.944045	-0.950441
C	-3.651880	-2.969697	-2.378323
C	-5.008618	-3.637270	-0.369559
C	-1.638907	-2.327198	-0.235105
O	-0.903037	-2.704478	-1.114598
H	-1.944805	-4.906309	-1.383966
H	-2.871060	-5.309895	0.075472
H	-3.545854	-5.649998	-1.522991
H	-2.658217	-2.920944	-2.817786
H	-4.286715	-3.586946	-3.016789

H	-4.077209	-1.965243	-2.344020
H	-5.642250	-4.293871	-0.967292
H	-5.458243	-2.644536	-0.342170
H	-4.962565	-4.024953	0.648304
C	-8.193494	-0.858580	2.555812
C	-6.735183	-0.614211	2.163394
C	-5.790416	-1.601105	2.884084
C	-5.987515	-1.524856	4.413039
C	-7.449625	-1.764339	4.794577
C	-8.387349	-0.793117	4.072948
H	-8.503879	-1.845780	2.193904
H	-6.441591	0.404793	2.433056
H	-6.061528	-2.614955	2.561217
H	-5.669563	-0.534958	4.753607
H	-7.726548	-2.793195	4.536260
H	-8.183323	0.227107	4.417484
H	-8.836041	-0.127785	2.058012
H	-6.603949	-0.706104	1.082972
H	-5.335387	-2.251928	4.901164
H	-7.567239	-1.672819	5.877422
H	-9.427386	-1.009548	4.330481

2.16. Cartesian coordinates of Ag^{+} (charge +1)

	x	y	z
Ag	0	0	0

2.17. Cartesian coordinates of TS1 (charge +1)

	x	y	z
C	3.662499	1.593233	0.892982
C	2.583750	0.828237	0.447794
C	2.742327	-0.112656	-0.588684
C	3.966630	-0.322143	-1.211081
C	5.029403	0.449303	-0.755684
C	4.884525	1.390811	0.276612
N	1.460031	-0.670891	-0.781756
C	0.630557	-0.040899	0.186502
N	1.276111	0.839290	0.896989
H	3.530279	2.310761	1.691017
H	4.092533	-1.053276	-1.993015
H	6.002080	0.313446	-1.210288
H	5.746631	1.962340	0.593948
O	-0.131368	-1.762644	-2.009155
C	-0.613631	-2.813227	-2.980986
C	-0.210898	-4.190798	-2.474602
C	-0.090010	-2.499152	-4.374493
C	-2.126908	-2.641733	-2.917571

C	1.194757	-1.631358	-1.766535
O	2.063921	-2.227642	-2.335418
H	0.865031	-4.332860	-2.526146
H	-0.543543	-4.336929	-1.445540
H	-0.687004	-4.949563	-3.097279
H	0.985118	-2.639017	-4.438015
H	-0.569191	-3.168705	-5.090606
H	-0.332475	-1.472839	-4.657906
H	-2.618773	-3.336930	-3.598092
H	-2.425766	-1.637707	-3.274399
H	-2.512766	-2.836994	-1.914573
C	-0.731798	-0.340783	0.427389
C	-1.811597	-0.575121	0.960331
C	-3.014306	-0.885989	1.643994
C	-3.567129	-2.175685	1.554937
C	-4.732284	-2.472743	2.241314
C	-5.351754	-1.497284	3.020898
C	-4.804597	-0.219950	3.116965
C	-3.642526	0.094202	2.430791
Ag	-2.038452	-0.198044	-1.509400
H	-3.068302	-2.933682	0.964424
H	-5.156076	-3.466474	2.178430
H	-6.260316	-1.735621	3.558654
H	-5.284963	0.531309	3.729858
H	-3.207224	1.082107	2.502537

2.18. Cartesian coordinates of $TSIIa_{endo}$ (charge +1)

	x	y	z
C	3.029894	0.064291	0.384387
C	3.302187	-0.560462	-0.849603
C	4.618440	-0.817103	-1.218991
C	5.622499	-0.439491	-0.337404
C	5.330944	0.179860	0.885466
C	4.021776	0.445845	1.273829
N	2.128094	-0.840461	-1.548771
C	1.141461	-0.424168	-0.823682
N	1.624193	0.158201	0.417536
C	0.789775	0.647496	1.329172
O	-0.492913	0.606234	1.119616
C	-1.064325	0.061039	-0.101761
C	-0.262835	-0.458632	-1.041527
C	-2.518277	0.197373	-0.058938
C	-3.230356	-0.028912	1.124958
C	-4.614678	0.067541	1.138599
C	-5.305038	0.399488	-0.023209
C	-4.603752	0.640438	-1.201000

C	-3.219781	0.543251	-1.220380
H	4.837856	-1.295220	-2.163646
H	6.655896	-0.626789	-0.597911
H	6.142000	0.458844	1.544895
H	3.796560	0.923027	2.215059
Ag	-0.898085	-1.416820	-2.817994
H	-2.703565	-0.309692	2.026799
H	-5.157262	-0.124368	2.055287
H	-6.384449	0.476394	-0.009326
H	-5.135254	0.919728	-2.101584
H	-2.672383	0.769846	-2.126425
C	0.465526	1.827287	3.536824
C	-0.384888	0.756886	4.193670
C	-0.331132	2.977555	2.948558
C	1.586789	2.306550	4.439075
H	-0.845665	1.184849	5.085173
H	0.314187	3.646534	2.378167
H	2.193665	1.470556	4.786299
H	-0.755947	3.550160	3.774482
H	-1.181525	0.410586	3.539490
H	0.226221	-0.090601	4.505300
H	2.225040	3.023087	3.922286
H	-1.150185	2.637328	2.319301
H	1.153142	2.799772	5.309536
O	1.274395	1.156176	2.399288

2.19. Cartesian coordinates of TSII'a$_{endo}$ (charge +1)

	x	y	z
C	2.955389	0.057893	0.340510
C	3.204469	-0.595966	-0.883291
C	4.513818	-0.856049	-1.274240
C	5.534531	-0.452693	-0.423732
C	5.265976	0.195189	0.789588
C	3.964070	0.465223	1.199077
N	2.017146	-0.897993	-1.550260
C	1.044153	-0.468057	-0.814784
N	1.550285	0.146767	0.401244
C	0.733048	0.655756	1.317627
O	-0.553571	0.604182	1.136314
C	-1.148125	0.025829	-0.058482
C	-0.364299	-0.513694	-1.001888
C	-2.601467	0.157041	0.011928
C	-3.287237	-0.044790	1.215575
C	-4.671323	0.046866	1.256522
C	-5.387492	0.349696	0.102408
C	-4.712437	0.565841	-1.095324

C	-3.328812	0.473326	-1.141978
H	4.715394	-1.356309	-2.211369
H	6.563052	-0.641946	-0.701525
H	6.089473	0.493558	1.424653
H	3.756611	0.964290	2.133013
Ag	-1.032581	-1.518582	-2.739996
H	-2.740259	-0.303158	2.112079
H	-5.193582	-0.126100	2.188673
H	-6.466674	0.423087	0.137579
H	-5.264148	0.822266	-1.990529
H	-2.801623	0.680706	-2.064459
C	0.449583	1.892494	3.499239
C	-0.388036	0.839138	4.198995
C	-0.358571	3.027070	2.896460
C	1.587121	2.394598	4.368141
H	-0.830529	1.289325	5.088784
H	0.275926	3.682113	2.298411
H	2.200398	1.567623	4.725657
H	-0.769881	3.619798	3.714972
H	-1.197813	0.477676	3.569673
H	0.228923	-0.000922	4.519063
H	2.215626	3.097212	3.821289
H	-1.188005	2.670321	2.290302
H	1.169475	2.910287	5.233401
O	1.237659	1.193329	2.364161

2.20. Cartesian coordinates of $TSIIb_{exo}$ (charge +1)

	x	y	z
C	3.391689	-0.319611	0.161007
C	3.319653	0.995250	0.684490
C	4.487954	1.637192	1.073059
C	5.687149	0.946923	0.926393
C	5.732905	-0.351889	0.406836
C	4.574393	-1.016746	0.010833
N	1.990329	1.461274	0.726436
C	1.280009	0.489964	0.255191
N	2.047648	-0.636086	-0.112297
C	1.241577	-1.582874	-0.608752
O	-0.008444	-1.201156	-0.595521
C	-0.089576	0.200655	-0.033394
C	-1.193641	0.933667	0.088054
C	-2.564482	0.600618	-0.228735
C	-2.999270	-0.722973	-0.449584
C	-4.322419	-0.992066	-0.754821
C	-5.245850	0.046726	-0.859641
C	-4.839537	1.359311	-0.641336

C	-3.519693	1.628818	-0.316992
H	4.457214	2.640438	1.475234
H	6.611189	1.426052	1.221876
H	6.687195	-0.851911	0.310285
H	4.602689	-2.019900	-0.390856
Ag	-0.556453	2.850465	0.891265
H	-2.297760	-1.538719	-0.364734
H	-4.641603	-2.014953	-0.909567
H	-6.278906	-0.168667	-1.100899
H	-5.554192	2.168860	-0.712770
H	-3.205381	2.649203	-0.133149
C	0.773916	-3.830385	-1.620093
C	0.059399	-3.274050	-2.837132
C	-0.153401	-4.293801	-0.513065
C	1.814952	-4.870742	-1.980759
H	-0.458505	-4.098636	-3.329609
H	0.410706	-4.588891	0.372211
H	2.514877	-4.484166	-2.721148
H	-0.698239	-5.169387	-0.869983
H	-0.679959	-2.520201	-2.576206
H	0.770984	-2.855451	-3.549395
H	2.367649	-5.193485	-1.098878
H	-0.882851	-3.533768	-0.241546
H	1.310572	-5.738855	-2.406574
O	1.674202	-2.700484	-1.034344

2.21. *Cartesian coordinates of TSII'b$_{exo}$ (charge +1)*

	x	y	z
C	3.347991	-0.280019	0.168407
C	3.267352	1.024034	0.717039
C	4.431501	1.666317	1.117273
C	5.635264	0.987157	0.956810
C	5.689544	-0.301055	0.412342
C	4.535320	-0.966012	0.004173
N	1.934928	1.480173	0.768162
C	1.230963	0.513235	0.278449
N	2.005980	-0.600155	-0.110813
C	1.205750	-1.543749	-0.622635
O	-0.046716	-1.170683	-0.602057
C	-0.136988	0.220310	-0.013465
C	-1.245513	0.943225	0.126957
C	-2.615919	0.611924	-0.192671
C	-3.041449	-0.703586	-0.473008
C	-4.365059	-0.969843	-0.778461
C	-5.298891	0.064054	-0.823461
C	-4.902111	1.368307	-0.546165

C	-3.581176	1.634235	-0.223344
H	4.394249	2.661503	1.538501
H	6.556206	1.466849	1.260937
H	6.647151	-0.792593	0.305653
H	4.570174	-1.961008	-0.416843
Ag	-0.613345	2.845572	0.970748
H	-2.332043	-1.515970	-0.435943
H	-4.676565	-1.986861	-0.980377
H	-6.332322	-0.149140	-1.065079
H	-5.624487	2.173718	-0.571723
H	-3.273753	2.647680	0.005098
C	0.752324	-3.778317	-1.668432
C	0.032917	-3.208794	-2.876462
C	-0.170611	-4.264531	-0.567592
C	1.799856	-4.806145	-2.045746
H	-0.480711	-4.029227	-3.380227
H	0.396535	-4.569616	0.312335
H	2.496403	-4.403813	-2.780968
H	-0.710224	-5.138038	-0.937285
H	-0.710586	-2.463145	-2.603952
H	0.741033	-2.775524	-3.583378
H	2.355688	-5.138369	-1.169399
H	-0.904613	-3.513589	-0.283197
H	1.300844	-5.671119	-2.484075
O	1.645579	-2.651231	-1.067006

2.22. Cartesian coordinates of the species Ia$_{endo}$

	x	y	z
C	2.810065	0.138288	0.489350
C	3.074663	-0.028864	-0.887678
C	4.393619	-0.054222	-1.337842
C	5.404447	0.087020	-0.396858
C	5.119184	0.251499	0.966824
C	3.810921	0.280492	1.438856
N	1.892133	-0.149698	-1.609612
C	0.922500	-0.057813	-0.736053
N	1.415095	0.119900	0.577529
C	0.622606	0.221662	1.703294
O	-0.713739	0.172072	1.446536
C	-1.264201	-0.004877	0.167281
C	-0.487755	-0.136013	-0.924616
C	-2.729782	-0.051317	0.252223
C	-3.354338	-0.562854	1.396597
C	-4.738802	-0.643350	1.466287
C	-5.523628	-0.201361	0.406137
C	-4.912440	0.334241	-0.724020

C	-3.529010	0.412282	-0.798995
O	1.033628	0.348910	2.823365
H	4.608807	-0.180701	-2.390627
H	6.437830	0.070340	-0.719815
H	5.935398	0.358430	1.669868
H	3.576435	0.406219	2.485432
Ag	-1.058523	-0.568718	-2.897419
H	-2.747860	-0.901483	2.224850
H	-5.206138	-1.052267	2.353503
H	-6.603216	-0.260829	0.464953
H	-5.516092	0.707332	-1.542265
H	-3.059206	0.865334	-1.661947

2.23. Cartesian coordinates of the species Ib_{exo}

	x	y	z
C	3.344634	-0.346807	0.199153
C	3.269086	1.016880	0.593201
C	4.435201	1.690046	0.944817
C	5.634051	0.988312	0.894009
C	5.684577	-0.356595	0.502691
C	4.533301	-1.052823	0.145714
N	1.944764	1.476251	0.557307
C	1.257001	0.440156	0.161455
N	2.020200	-0.683103	-0.073276
C	1.227747	-1.754984	-0.488823
O	-0.059648	-1.281661	-0.513588
C	-0.113711	0.099292	-0.112609
C	-1.194318	0.881754	-0.014865
C	-2.580859	0.541659	-0.287936
C	-2.987537	-0.733978	-0.732380
C	-4.324772	-1.006355	-0.978419
C	-5.295153	-0.025285	-0.791282
C	-4.915319	1.239386	-0.353414
C	-3.578723	1.514593	-0.107338
H	4.399173	2.728101	1.247547
H	6.553841	1.491782	1.163452
H	6.639167	-0.866055	0.477260
H	4.559341	-2.090115	-0.157879
Ag	-0.502328	2.815697	0.648684
H	-2.250213	-1.507540	-0.882675
H	-4.612495	-1.993501	-1.319231
H	-6.337658	-0.245292	-0.985609
H	-5.660740	2.010880	-0.204305
H	-3.280365	2.499230	0.233102
O	1.563437	-2.864889	-0.772297

2.24. Cartesian coordinates of the molecule 5b

	x	y	z
C	2.823770	0.100132	0.494606
C	3.071521	0.050439	-0.894704
C	4.383955	0.067599	-1.362614
C	5.405612	0.133170	-0.425262
C	5.137006	0.181403	0.950890
C	3.835139	0.165794	1.440739
N	1.880524	-0.011475	-1.612999
C	0.929520	0.001308	-0.722759
N	1.428608	0.068205	0.599023
C	0.651342	0.084485	1.736149
O	-0.698651	0.052295	1.489405
C	-1.261615	-0.018006	0.229251
C	-0.482942	-0.055769	-0.871850
C	-2.723576	-0.064056	0.276880
C	-3.384124	-0.377581	1.471417
C	-4.770653	-0.440931	1.509472
C	-5.517551	-0.188807	0.364111
C	-4.868402	0.133678	-0.825305
C	-3.484734	0.199330	-0.870144
O	1.062427	0.127386	2.859397
H	4.584680	0.030388	-2.424928
H	6.434849	0.147574	-0.760827
H	5.961862	0.231746	1.649978
H	3.615201	0.202237	2.497363
H	-0.900711	-0.142413	-1.860898
H	-2.806187	-0.572715	2.363274
H	-5.268290	-0.688423	2.438647
H	-6.598683	-0.236344	0.397265
H	-5.443546	0.344082	-1.718119
H	-2.996349	0.475269	-1.795177

2.25. Cartesian coordinates of the molecule 6b

	x	y	z
C	3.050333	-0.545297	0.000000
C	3.066852	0.874887	0.000000
C	4.288376	1.542784	0.000000
C	5.448188	0.778234	0.000000
C	5.406622	-0.623542	0.000000
C	4.199776	-1.316074	0.000000
N	1.767851	1.400804	0.000000
C	1.001392	0.353311	0.000000
N	1.693386	-0.853113	0.000000
C	0.835269	-1.940631	0.000000

O	-0.442756	-1.402451	0.000000
C	-0.399116	0.001020	0.000000
C	-1.477460	0.795160	0.000000
C	-2.888931	0.459064	0.000000
C	-3.380931	-0.858392	0.000000
C	-4.747225	-1.094849	0.000000
C	-5.651334	-0.035262	0.000000
C	-5.178826	1.273683	0.000000
C	-3.813979	1.516996	0.000000
O	1.077122	-3.106944	0.000000
H	4.319143	2.624023	0.000000
H	6.410222	1.274807	0.000000
H	6.334411	-1.180932	0.000000
H	4.157089	-2.396274	0.000000
H	-1.233096	1.851805	0.000000
H	-2.692318	-1.689458	0.000000
H	-5.110533	-2.114962	0.000000
H	-6.716680	-0.229345	0.000000
H	-5.874107	2.103758	0.000000
H	-3.448740	2.537191	0.000000

2.26. Cartesian coordinates of isobutylene

	x	y	z
C	0.122978	0.176824	-0.018765
C	0.885152	1.273422	-0.133786
C	0.715720	-1.201552	0.112027
C	-1.381621	0.243514	-0.010912
H	0.444060	2.267571	-0.227493
H	1.975078	1.212669	-0.138022
H	0.392222	-1.680483	1.051174
H	1.813170	-1.177772	0.097545
H	0.372778	-1.856137	-0.706437
H	-1.745464	1.274427	-0.110691
H	-1.805607	-0.354556	-0.834525
H	-1.788492	-0.178465	0.922940

2.27. Cartesian coordinates of TFA

	x	y	z
C	-0.800793	-0.026695	0.018073
C	0.591474	0.691616	0.075917
O	1.454308	0.198451	0.988300
O	0.840180	1.608467	-0.658531
F	-0.972238	-0.619115	-1.177811
F	-0.901609	-1.000643	0.985780
F	-1.794384	0.854922	0.217189
H	1.035667	-0.539001	1.479018

2.28. Cartesian coordinates of anion TFA (Charge -1)

	x	y	z
C	-0.593034	0.038451	0.000000
C	1.004796	-0.035012	0.000000

O	1.581054	1.074563	0.000000
O	1.420749	-1.215737	0.000000
F	-1.118262	1.308332	0.000000
F	-1.147652	-0.585298	1.100062
F	-1.147652	-0.585298	-1.100062

3. References:

1. Ahlrichs, R.; Bär, M.; Häser, M.; Horn, H.; Kölmel, C. Electronic Structure Calculations on Workstation Computers: The Program System Turbomole. *Chem. Phys. Lett.* **1989**, *162*, 165–169.
2. Jiménez-Hoyos, C.A.; Janesko, B.G.; Scuseria, G.E. Evaluation of range-separated hybrid density functionals for the prediction of vibrational frequencies, infrared intensities, and Raman activities. *Phys. Chem. Chem. Phys.* **2008**, *10*, 6621–6629.
3. Weigend, F.; Häser, M. RI-MP2: First Derivatives and Global Consistency. *Theor. Chem. Acc.* **1997**, *97*, 331–340.
4. Weigend, F.; Häser, M.; Patzelt, H.; Ahlrichs, R. RI-MP2: Optimized Auxiliary Basis Sets and Demonstration of Efficiency. *Chem. Phys. Lett.* **1998**, *294*, 143–152.
5. Becke, A.D. Density-Functional Exchange-Energy Approximation with Correct Asymptotic Behavior. *Phys. Rev. A.* **1988**, *38*, 3098–3100.
6. Lee, C.; Yang, W.; Parr, R.G. Development of the Colle-Salvetti Correlation-Energy Formula into a Functional of the Electron Density. *Phys. Rev. B* **1988**, *37*, 785–789.
7. Grimme, S. Semiempirical GGA-Type Density Functional Constructed with a Long-Range Dispersion Correction. *J. Comput. Chem*. **2006**, *27*, 1787–1799.
8. Grimme, S., Antony, J., Ehrlich, S., Krieg, H. A Consistent and Accurate Ab Initio Parametrization of Density Functional Dispersion Correction (DFT-D) for the 94 Elements H-Pu. *J. Chem. Phys*. **2010**, *132*, 154104.
